# Spectrum-Effect Relationship Analysis of Bioactive Compounds in *Zanthoxylum nitidum* (Roxb.) DC. by Ultra-High Performance Liquid Chromatography Mass Spectrometry Coupled With Comprehensive Filtering Approaches

**DOI:** 10.3389/fphar.2022.794277

**Published:** 2022-03-09

**Authors:** Si-wei Rao, Yuan-yuan Duan, Han-qing Pang, Shao-hua Xu, Shou-qian Hu, Ke-guang Cheng, Dong Liang, Wei Shi

**Affiliations:** ^1^ State Key Laboratory for Chemistry and Molecular Engineering of Medicinal Resources, Collaborative Innovation Center for Guangxi Ethnic Medicine, School of Chemistry and Pharmaceutical Science, Guangxi Normal University, Guilin, China; ^2^ Institute of Translational Medicine, Medical College, Jiangsu Key Laboratory of Integrated Traditional Chinese and Western Medicine for Prevention and Treatment of Senile Diseases, Yangzhou University, Yangzhou, China

**Keywords:** *Zanthoxylum nitidum* (Roxb.) DC., spectrum–effect relationship, chemical profiling, anti-inflammation, antioxidant activity

## Abstract

*Zanthoxylum nitidum* (Roxb.) DC. (ZN), with strong effects of anti-inflammation and antioxidant activities is treated as a core herb in traditional Chinese medicine (TCM) preparation for treating stomachache, toothache, and rheumatoid arthritis. However, the active ingredients of ZN are not fully clarified due to its chemical complexity. In the present study, a double spectrum–effect analysis strategy was developed and applied to explore the bioactive components in herbs, and ZN was used as an example. Here, the chemical components in ZN were rapidly and comprehensively profiled based on the mass defect filtering-based structure classification (MDFSC) and diagnostic fragment-ion-based extension approaches. Furthermore, the fingerprints of 20 batches of ZN samples were analyzed by high-performance liquid chromatography, and the anti-inflammatory and antioxidant activities of the 20 batches of ZN samples were studied. Finally, the partial least squares regression (PLSR), gray relational analysis models, and Spearman’s rank correlation coefficient (SRCC) were applied to discover the bioactive compounds in ZN. As a result, a total of 48 compounds were identified or tentatively characterized in ZN, including 35 alkaloids, seven coumarins, three phenolic acids, two flavonoids, and one lignan. The results achieved by three prediction models indicated that peaks **4**, **12**, and **17** were the potential anti-inflammatory compounds in ZN, whereas peaks **3**, **5**, **7**, **12**, and **13** were involved in the antioxidant activity. Among them, peaks **4**, **5**, **7**, and **12** were identified as nitidine, chelerythrine, hesperidin, and oxynitidine by comparison with the standards and other references. The data in the current study achieved by double spectrum–effect analysis strategy had great importance to improve the quality standardization of ZN, and the method might be an efficiency tool for the discovery of active components in a complex system, such as TCMs.

## Introduction

Generally, traditional Chinese medicines (TCMs) achieve their therapeutic effects by initial interactions via multicomponents and multitargets. To understand how it works, it is necessary for researchers to study the relationship between TCMs’ compounds and their efficacy from a holistic perspective (Jiang et al., 2010). A pharmacological study of single-compound and holistic studies of TCMs are not birds of a feather; however, their research ideas and methods cannot be generalized (Xie and Leung, 2009). Although the above studies contributed to reveal the mechanism of pharmacological efficacy of TCMs, it is also meaningful to unravel the details of TCMs’ mechanism with important implications to quality control of TCMs and treatment of complicated diseases.

*Zanthoxylum nitidum* (Roxb.) DC. belongs to the genus *Zanthoxylum* of family Rutaceae (Seidemann, 2005; Rivera et al., 2014), and its underground roots (ZN) are used as the medicinal part recorded in the Chinese Pharmacopoeia (China Pharmacopoeia Commission, 2020). ZN has excellent curative effects, such as for the treatment of toothache, stomachache, traumatism, and rheumatoid arthritis. In daily life, ZN could be used as toothpaste and hand sanitizer. In a previous study, researchers were mainly focused on the chemical isolation and activity evaluation of ZN (Yang et al., 2008; Zhao et al., 2018; Nguyen et al., 2019). ZN extracts showed good anti-inflammatory and antioxidant activities (Xie, 2000; Liu et al., 2014). Alkaloids, the major component in ZN, have earned an increasing interest (Lu et al., 2020). Nitidine, a typical single alkaloid in ZN, has been found to have antifungal and anti-inflammatory activity (Zhang et al., 2014; Zhang et al., 2017). However, the components in ZN were very complicated, and some other active ingredients could also exist. To screen the active compounds rapidly, a comprehensive qualitative strategy and spectrum–effect relationship analysis is worth to establish.

The spectrum–effect relationship analysis is a tried-and-true method in using stoichiometric methods to figure out the connection between efficacy and components (Wang et al., 2019; Chen et al., 2021; Qiao et al., 2021). Zhang et al. developed the spectrum–effect relationship analysis strategy to discover the active compounds in Lycii Fructus; the results showed that chlorogenic acid, quercetin, kaempferol, and isorhamnetin are their potential bioactive components (Zhang et al., 2018). Moreover, a strategy-contained spectrum–effect relationship analysis was proposed to discover hepatotoxic equivalent markers from Psoraleae Fructus, and the results revealed that psoralen and isopsoralen are the hepatotoxic equivalent components (Zhang et al., 2021). The above studies have further verified the effectiveness of the spectrum–effect relationship analysis method, which were confirmed to effectively predict the active compounds in the complicated matrix.

**FIGURE 1:**
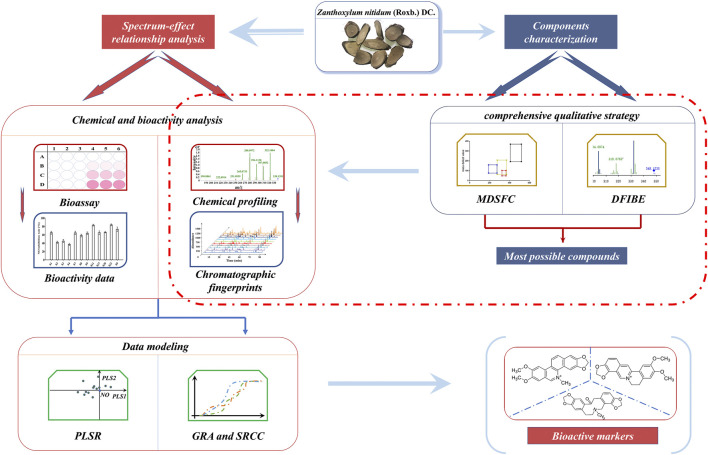
The strategy for discovering bioactive markers in *Zanthoxylum nitidum* (Roxb.) DC. (ZN) by ultrahigh-performance liquid chromatography-quadrupole time-of-flight mass spectrometry (UHPLC Q-TOF MS) coupled with comprehensive filtering approaches.

In this study, a comprehensive filtering approach and spectrum–effect relationship were applied to discover the bioactive components in ZN (Figure 1). First, based on the mass defect filtering-based structure classification (MDFSC) and diagnostic fragment-ion-based extension (DFIBE) approaches, the chemical compounds of ZN were rapidly profiled by the ultrahigh-performance liquid chromatography-quadrupole time-of-flight mass spectrometry (UHPLC Q-TOF MS). Second, the fingerprints of 20 batches of ZN samples were established by high-performance liquid chromatography (HPLC), followed by similarity analysis (SA) and hierarchical cluster analysis (HCA). Furthermore, different activity tests including 3-(4,5-dimethylthiazol-2-yl)- 2,5-diphenyltetrazolium bromide (MTT) test, NO production assay, and the 2,2-diphenyl-1-picrylhydrazyl (DPPH) assay were determined, respectively. Finally, to discover the potential active compounds, the spectrum–effect relationship was modeled by chemometrics such as the partial least squares regression (PLSR), gray relational analysis (GRA), and Spearman’s rank correlation coefficient (SRCC).

## Materials and Methods

### Materials and Reagents

Twenty batches of ZN samples were collected from different areas in China, which contain the resource areas that are shown in Supplementary Table S1). The voucher specimens were identified by Prof. Dong Liang from the Department of Chemistry and Pharmacy in Guangxi Normal University and were stored in the State Key Laboratory for the Chemistry and Molecular Engineering of Medicinal Resources, Guilin, China.

Liquid chromatography mass spectrometry (LC-MS)-grade acetonitrile and methanol were bought from MEDIA (Fairfield, CT, United States). Formic acid was HPLC grade and purchased from Aladdin (Shanghai, China). Chelerythrine, diosmin, hesperidin, nitidine chloride, sanguinarine, magnoflorine, and dihydrochelerythrine, all with purity of ≥98%, were obtained from Chengdu Push Bio-Technology (Chengdu, China). Deionized water was purified using a Milli-Q water purification system (Millipore, United States). Dulbecco’s modified Eagle’s medium (DMEM) was produced by Gibco (Grand Island, NE, United States), and DPPH was produced by Tokyo Chemical Industry (Tokyo, Japan).

### Preparation of *Zanthoxylum nitidum* (Roxb.) DC. Extracts

After being ground into powder and air-dried (filtered with a 50-mesh sieve), each ZN sample (5 g) was dissolved with 100 ml of 80% ethanol and extracted three times at 3, 2, and 1 h on a water bath at 80°C. Then a Rotavapor OSB-2200 from EYELA Co. (Tokyo, Japan) was used to remove the solvent. The extracts were powdered by an FD5-series vacuum freeze dryer (GOLD SIM, Newark, NJ, United States) and then stored for use.

### Chromatographic and Mass Spectrometric Condition

#### Chemical Profiling of *Zanthoxylum nitidum* (Roxb.) DC. Sample by UHPLC Q-TOF MS

The comprehensive characterization of ZN samples was conducted on an Agilent 6545 UHPLC Q-TOF MS (Agilent Technologies, Santa Clara, CA, United States) with the monitoring of Agilent LC-QTOF/MS Mass Hunter Workstation Acquisition Software Version B.05.01 (Agilent Technologies, Santa Clara, CA, United States). Tested samples were separated on an Agilent Zorbax clipse Plus C18 column (2.1 × 50 mm, 1.8-μm, Agilent Corp., Santa Clara, CA, United States). The mobile phase consisted of acetonitrile (Santa Clara, CA, United States). The mobile phases were 0.1% formic acid (A) and acetonitrile (B) with the following gradient elutions: 5–15% B linear 0–3 min, 15–20% B linear 3–6 min, 20–22% B linear 6–7 min, 22% B isocratic 7–9 min, 22–26% B linear 9–12 min, 26–28% B linear 12–13 min, 28% B isocratic 13–15 min, 28–34% B linear 15–16 min, 34–38% B linear 16–18 min, 38–40% B linear 18–20 min, 40–55% B linear 20–24 min, 55–65% B linear 24–25 min, 65–85% B linear 25–28 min, and 85–95% B linear 28–30 min. The injection volume and the detection wavelength were set as 0.6 μl and at 254 nm, respectively. The flow rate was 0.6 ml/min, and the column temperature was maintained at 35°C.

Both MS and MS/MS were performed in positive ion mode, and the ion source is dual AJS ESI. The MS parameters were set as follows: capillary voltage, 3,500 V; nebulizer gas (N_2_) pressure, 30 psig; drying gas flow rate, 8.0 L/min; drying gas (N_2_) temperature, 320°C; shealth gas flow rate, 12.0 L/min; shealth gas (N_2_) temperature, 350°C; OCT RF V, 750 V; skimmer, 65 V; fragmentor, 135 V. The scan ranges for product ions were *m/z* 100–3,000, and the collision energy was set at 20, 40, and 60 V. Before the analysis, mass spectrometer of the TOF was calibrated at *m/z* 121.0508 and 922.0098 in positive ion mode to ensure the mass accuracy. Data acquisition and analysis were obtained by Agilent LC-MS MassHunter Workstation Software (version B.08.00).

### Chemical Fingerprint Analysis of *Zanthoxylum nitidum* (Roxb.) DC. Sample by HPLC

The SHIMADZU LC-20AT HPLC system was employed to perform the HPLC fingerprint analysis. The system consists of quad pump, online vacuum degassing machine, DAD detector, column temperature box, and automatic sampler (SHIMADZU LabTotal, Tokyo, Japan). Chromatographic separation was conducted on a column of symmetry columns (4.6 × 250 mm, 5 μm, Waters, Milford, CT, United States). The mobile phase consisted of water with 0.1% formic acid (A) and acetonitrile (B). The gradient conditions are shown in Supplementary Table S2. The temperature was set at 35°C. The purpose of the final 10 min is re-equilibrizing the column. The flow rate was at 1 ml/min, and the injection volume and detection wavelength were set at 10 μl and 254 nm, respectively.

### Antioxidant and Anti-Inflammatory Bioactivity Assay

#### Antioxidant Activity Assay

In this study antioxidant activities were measured by the classic test DPPH assays. DPPH assay is a chemical analysis experiment, which is used in a basic *vitro* screening method for evaluating the radical scavenging activity (Heinrich et al., 2020). The radical scavenging activity (RSA) was calculated by the following formula:

$$RSA\left(\%\right)=\left[1-\frac{\left(A_{sample\;}\;-A_{blank\;}\;\right)}{\left(A{\;}_{control\;}-A_{blank\;}\;\right)}\right]\times \;100$$ (1)

where A_control_ is the absorbance of 100 μl of DPPH solution with 100 μl of ethanol, A_blank_ is the absorbance of 200 μl of ethanol, and A_sample_ is the absorbance of 100 μl of DPPH solution with 100 μl of sample or ascorbic acid solution.

The DPPH assay was performed based on the instructions described in the literature (Kang et al., 2011; Wang et al., 2017; Zhang et al., 2018). An aliquot of 100 μl of each sample in ethanol (10, 25, 50, 100, 200 μg/ml) was mixed with 100 μl of 0.2 mM DPPH ethanolic solution. Ascorbic acid was used as a positive drug. The mixture was incubated for 30 min in the darkroom, then the absorbance was measured at 517 nm. The determination was conducted in triplicates, and the antioxidant activity was expressed by the IC_50_ value.

### Anti-Inflammatory Activity Assay

Raw264.7 cells (100 μl) were seeded at 2.5 × 10^4^ cells per well into 96-Transwell insert plates and incubated in a 5% CO_2_ atmosphere at 37°C. After 24 h, the powder of ZN extracts was dissolved in the new culture medium containing 1 μg/ml of LPS, configured as a solution of 80 μg/ml to replace the old culture medium. Cells were maintained in the incubator at 37°C for 24 h. The level of nitric (an indicator of NO synthesis) was measured using nitric oxide assay kit (Beyotime, Shanghai, China) according to the manufacturer’s instructions. The OD value was measured at 540 nm with a microplate reader (BioTek, SynergyH1, United States). The calculation formula of inhibition rate of NO production is as follows:

$$\text{Rate}\left(\text{\%}\right)=\left[\frac{{\text{C}}_{\text{m}}-{\text{C}}_{\text{s}}}{{\text{C}}_{\text{m}}-{\text{C}}_{\text{n}}}\right]\times 100$$ (2)

C_m_ is the NO concentration of the model group, C_s_ is the NO concentration of the samples, and C_n_ is the NO concentration of the normal control group. Finally, the original cells in 96-well plates were used to determine the cell viability by an MTT assay (Wang et al., 2019).

### Spectrum–Effect Relationship Modeling

#### Establishing of HPLC Fingerprint and Hierarchical Cluster Analysis

Twenty batches of ZN samples were chemically profiled and matched automatically by using the “Chinese Medicine Chromatographic Fingerprint Similarity Evaluation System 2012.” Then the common peaks of 20 fingerprints were calculated by the multipoint method, and the control chromatogram was generated automatically by the average method.

The HCA of different batches of ZN samples was carried out with the SPSS statistics software (SPSS version 19.0, IBM Corp., Armonk, NY, United States). The between-group linkage method and the squared Euclidean distance were used to establish the clusters.

### Partial Least Squares Regression Model

PLSR is the statistical method, which is related to principal component regression, but is not a hyperplane that looks for the maximum variance between the response variable and the independent variable (Qiao et al., 2018). It is helpful in classification and biomarker discovery. The model is a multivariate calibration model used to find the inner relationship between an n × p data matrix X and an n × q response matrix Y (Martens and Naes, 1992). In this study, PLSR was used to model the fingerprint–activity relationship based on the SIMCA-P 14.0 Software (Umetrics AB, Umea, Sweden). The X-matrix was composed of the common peak areas in chromatographic fingerprints, and the Y-matrix was constructed with the anti-inflammatory and antioxidant activities.

$$X=TP^{T}+E$$ (3)

$$Y=UQ^{T}+F$$ (4)

where X is a prediction matrix of n×m , Y is a prediction matrix of n×p , *TP* ^*T*^ approximates to the common peak areas and *UQ* ^*T*^ to the activity values, and E and F contains the residuals of the regression model.

### Grey Relational Analysis Model

The Grey relational analysis is often used to measure the degree of correlation among factors according to the degree of similarity or dissimilarity between the development trends of factors, which is also called “grey correlation degree” (Elena Arce et al., 2015; Jiang et al., 2018). The anti-inflammatory and antioxidant activities of 20 ZN samples were selected as the reference sequence, and 18 relative common peaks were defined as comparability sequences. Then the grey relational coefficients (GRC) between common peaks and bioactivities were conducted through the Data Processing System (DPS 9.01) software. The formula for calculation relational degree is as follows:

$$r_{i}=\frac{1}{N}\overset{N}{\underset{k=1}{\sum }}\xi_{i}\left(k\right)$$ (5)

where $r_{i}$ is the correlation, $\xi_{i}$ is the grey relational coefficient, and N is the total sample number.

### Spearman’s Rank Correlation Coefficient

Spearman’s rank correlation coefficient (*ρ*) is a widely used method to measure the degree of correlation between two variables. A positive correlation coefficient value of ρ (from 0 to 1) implies that the two variables are positively correlated. On the contrary (from −1 to 0), there is a negative correlation. Besides, a correlation coefficient value of 0 implies that the two variables are not related (Zar, 2005).

In this study, Spearman’s rank correlation coefficient was used to quantify the correlation between 18 common peaks and bioactivities by using SPSS version 19.0 (IBM Corp., Armonk, NY, United States). The formula for the calculation of the relational degree is as follows:

$$\rho =\frac{\sum_{i}\left(x_{i}-\bar{x}\right)\left(y_{i}-\bar{y}\right)}{\sqrt{\sum_{i}{\left(x_{i}-\bar{x}\right)}^{2}\sum_{i}{\left(y_{i}-\bar{y}\right)}^{2}}}$$ (6)

where *xi* is the rank of the data of the random variable X, *yi* is the rank of the data of the random variable Y, and $\bar{x}$ and $\bar{y}$ are the expectations of X and Y, respectively.

## Results and Discussion

### Chemical Identification of *Zanthoxylum nitidum* (Roxb.) DC. Sample

#### Establishment of the Qualitative Strategy

**FIGURE 2:**
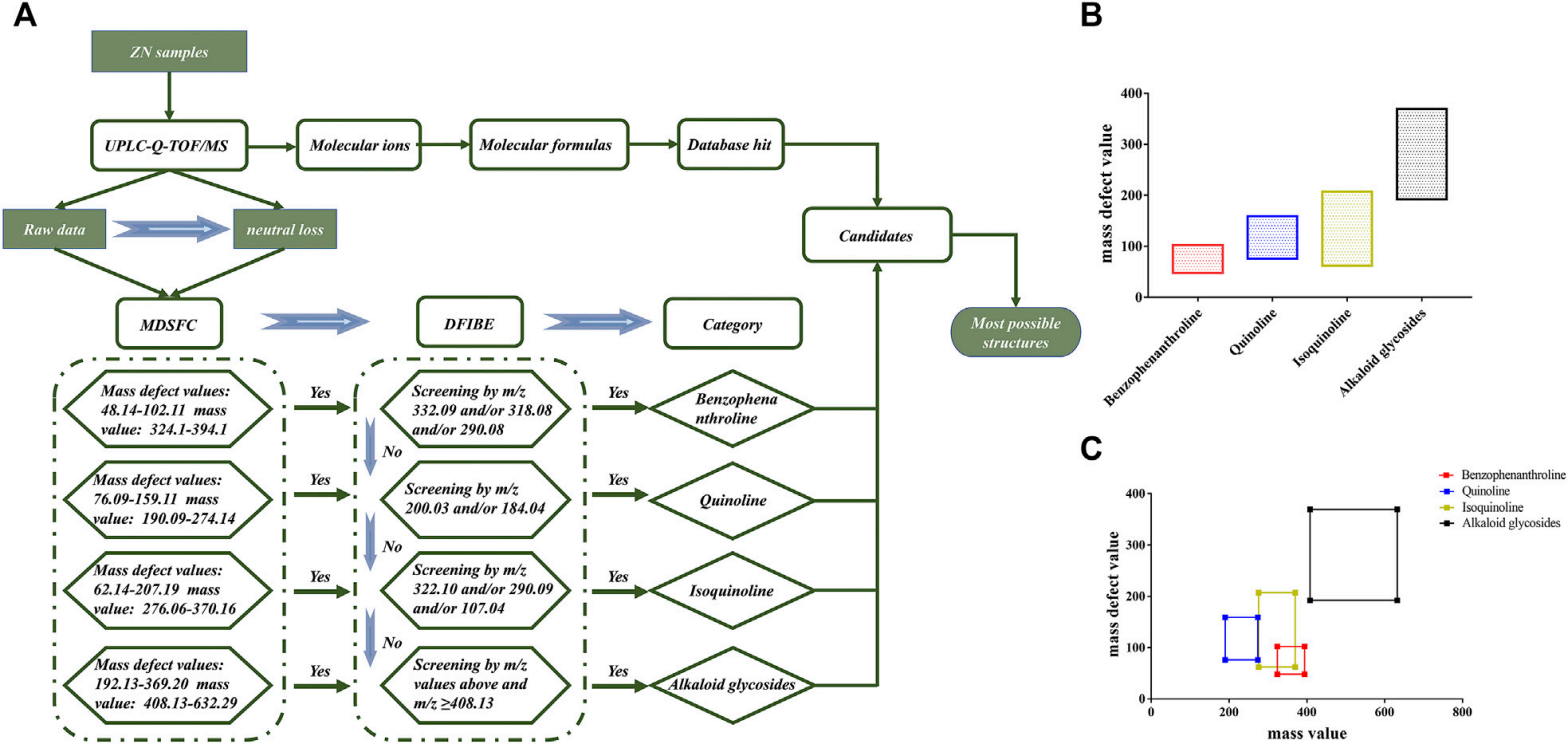
The structure-diagnostic ion-oriented network **(A)**. The mass defect ranges of different types of alkaloids in ZN **(B)**; the correlations between the mass ranges and mass shifts of the basic skeletons of ZN alkaloids **(C)**. MDFSC, mass defect filtering-based structure classification; DFIBE, diagnostic fragment-ion-based extension.

In TCMs, the same chemical structure compounds can be classified as a category with a unique mother skeleton. These compounds with similar structure group share identical diagnostic ions and mass defect value, which could make the structural identification more efficient (Zhang et al., 2008; Zhang et al., 2009). In this study, a structure-diagnostic ion-oriented network (Figure 2A) was established for the rapid characterization of alkaloids compounds in ZN: 1) The mass fragment behaviors of the compounds in ZN were summarized by referring to the existing reviews and database (Sci-finder, Google Scholar, and PubMed). 2) Total ions and product ion scan were used to create the comprehensive chemical profiling of ZN (Figures 2B,C). 3) Then the mass defect filtering-based structure classification (MDFSC) and diagnostic fragment-ion-based extension (DFIBE) were applied to process the MS data. 4) With the help of the classification method, most alkaloid compounds in ZN were rapidly identified by their mass fragmentation rules. A diagram for rapid classification and identification of the chemical compounds in ZN is displayed in Figure 2.

### MDFSC Approach for Structural Classification

With a well-preset mass defect window, the MDFSC approach can quickly find out the class of compound based on their similar mother skeleton over certain mass ranges, and can also obtain new compounds never found before (He et al., 2018; Zhou et al., 2018; Pang et al., 2019). Considering that alkaloids are just one part of compounds in ZN, the MDFSC strategy was only a preliminary structural classification for alkaloids in the whole identification of ZN.

On the basis of existing literatures (Jia et al., 2013; Yang et al., 2017; Fu et al., 2021) and authoritative database, we concluded the core substructures and corresponding substituents in ZN, and calculated their maximum and minimum mass defect values (the tolerance of mass defect is ±5 m Da). In total, the alkaloids can be divided into four categories: benzophenanthroline, quinoline, isoquinoline, and alkaloid glycosides. Respectively, mass defect values and mass values of these four categories are listed as follows: benzophenanthroline (mass defect values 48.14–102.11 m Da, mass values: 324.10–394.10 m Da), quinoline (mass defect values: 76.09–159.11 m Da, mass values: 190.09–274.14 m Da), isoquinoline (mass defect values: 62.14–207.19 m Da, mass values: 276.06–370.16 m Da), and alkaloid glycosides (mass defect values: 192.13–369.20 m Da, mass values: 408.13–632.29 m Da). Using the MDFSC approach, we could preliminarily sort part of the detected ions as the certain chemical homologs and discovered that some alkaloid glycosides have mother skeletons, which are compounds classified in the former three categories. For example, the mother skeleton of compound 39 was similar to jatrorrhizine; its MS/MS spectra had fragment peaks of 338.1390, 322.1077, and 294.1123 m*/z*, which correspond to jatrorrhizine.

### Structure Classification and Identification of Alkaloids in *Zanthoxylum nitidum* (Roxb.) DC. Sample

**FIGURE 3:**
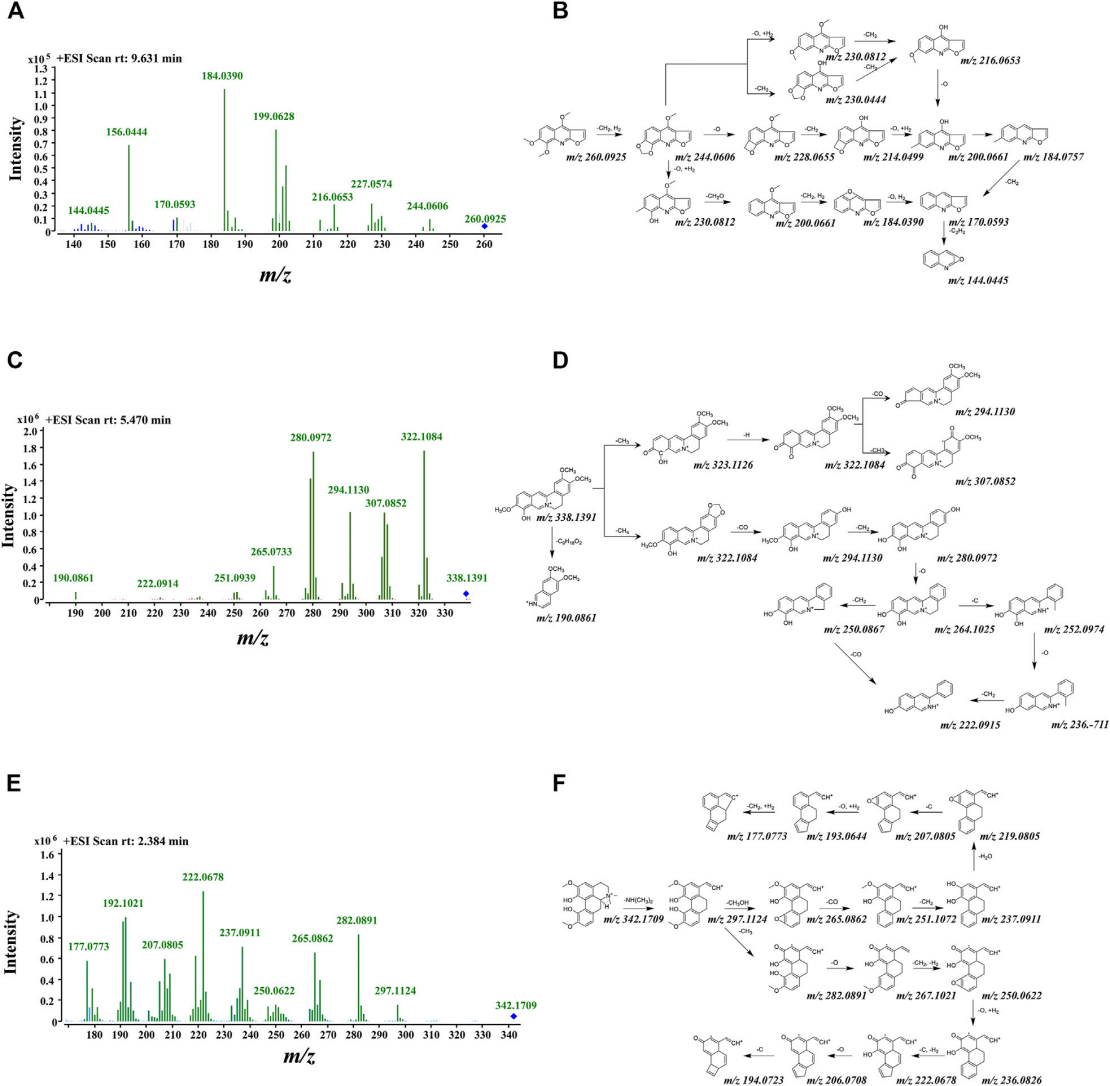
Tentative fragmentation pathway and mass spectrogram of several typical alkaloids in ZN. **(A,B)** Skimmianine; **(C,D)** jatrorrhizine; **(E,F)** magnoflorine.

Combined with the MDFSC approach and DFIBE, the structure-diagnostic ion-oriented strategy was employed to rapidly speculate the chemical constituents, and in total, 48 compounds were inferred from ZN. Among them, alkaloids accounted for 35 of the 48, which indicate the significance of alkaloids. All the detailed information is listed in Table 1, and the fragmentation pattern together with mass spectrogram of representative compounds are shown in Figure 3.

**TABLE 1:** Characterization of chemical constituents of *Zanthoxylum nitidum* (Roxb.) DC. (ZN) sample.

No.	T_R_ (min)	Identification	Formula	Theoretical mass (*m/z*)	Measured mass (*m/z*)	Error (ppm)	In mode	MS/MS (*m/z*)
1	0.32	L-Arginine	C_6_H_14_N_4_O_2_	175.1190	175.1194	−2.28	[M+H]^+^	158.0901, 144.1377, 130.0963, 116.0705, 100.0756
2	1.32	(E)-3-(3,4,5-trihydroxyphenyl) acrylaldehyde	C_9_H_8_O_4_	181.0495	181.0492	1.66	[M+H]^+^	153.0539, 140.0461, 125.0589, 110.0353
3	1.90	Chlorogenic acid	C_16_H_18_O_9_	355.1024	355.1036	−3.38	[M+H]^+^	266.0805, 193.0491, 163.0387, 135.0439, 117.0331
4	2.13	Magnocurarine A	C_19_H_24_NO_3_^+^	314.1756	314.1754	0.64	[M]^+^	269.1164, 237.0899, 175.0747, 107.0482
5	2.38	Magnoflorine A	C_20_H_24_NO_4_^+^	342.1705	342.1709	−1.46	[M]^+^	297.1124, 282.0891, 237.0911, 222.0678, 192.1021
6	3.14	Magnocurarine B	C_19_H_24_NO_3_^+^	314.1756	314.1771	−4.77	[M]^+^	314.1760, 269.1174, 237.0909, 175.0751, 107.0489
7	3.20	3′-hydroxy-4′,6,7-trimethoxyl-N,N-dimethyltetrahydroisoquinoline	C_21_H_28_NO_4_^+^	358.2018	358.2024	−1.68	[M]^+^	298.1196, 267.1011, 206.1171, 174.0617, 137.0594
8	3.42	D-Tertrahydropaimatine	C_21_H_25_NO_4_	356.1869	356.1856	−3.65	[M+H]^+^	192.1022, 177.0785, 137.0594
9	3.59	(Z)-3-(2-(2-hydroxypropan-2-yl)-2,3-dihydrobenzofuran-5-yl) acrylic acid	C_14_H_16_O_4_	249.1130	249.1121	3.61	[M+H]^+^	203.0703, 189.0548, 171.0437, 161.0590, 141.0694, 129.0695, 115.0538
10	4.00	Dimethyl 5-[(6-phenethylpyridin-3-yl) methyl] isophthalate	C_24_H_23_NO_4_	390.1574	390.1559	3.84	[M+H]^+^	330.0732, 295.0600, 254.0576, 209.0593, 167.0488
11	4.18	Protopine	C_20_H_19_NO_5_	354.1336	354.1349	−3.67	[M+H]^+^	320.0918, 247.0750, 192.1021, 149.0594, 107.0485
12	4.34	Analog of liriodenine	C_19_H_17_NO_4_	324.1241	324.1230	3.39	[M+H]^+^	309.0998, 281.1047, 266.0812, 192.1021, 177.0786, 145.0282, 117.0333
13	4.40	Tertrahydropaimatine	C_21_H_25_NO_4_	356.1869	356.1856	3.65	[M+H]^+^	340.1544, 192.1021, 177.0786, 145.0282
14	4.49	Analog of oxyavicine	C_21_H_21_NO_5_	368.1492	368.1498	−1.63	[M+H]^+^	352.1191, 324.1240, 310.1082, 292.0970, 264.0662, 204.0654
15	4.62	Magnoflorine B	C_20_H_24_NO_4_^+^	342.1705	342.1710	−1.17	[M]^+^	297.1123, 282.0886, 237.0906, 222.0672, 207.0801
16	5.16	Palmatrubin	C_20_H_19_NO_4_	338.1387	338.1401	−4.14	[M+H]^+^	307.0841, 265.0735, 237.0783, 190.0863, 175.0628
17	5.19	Allocryptopine	C_21_H_23_NO_5_	370.1649	370.1657	−2.16	[M+H]^+^	336.1223, 290.0932, 252.0774, 206.0802, 188.0698, 149.0591
18	5.21	10-Methoxy-2,3-dihydro-7H-[1,4] dioxino[2,3-g] chromen-7-one	C_12_H_10_O_5_	235.0601	235.0607	−2.55	[M+H]^+^	205.0497, 191.0341, 177.0547, 163.0389, 149.0230, 135.0441, 121.0283, 107.0488
19	5.47	Jatrorrhizine	C_20_H_19_NO_4_	338.1400	338.1391	2.66	[M+H]^+^	322.1091, 294.1130, 280.0972, 265.0733, 222.0914
20	5.62	Diosmin	C_28_H_32_O_15_	609.1814	609.1815	−0.16	[M+H]^+^	463.1234, 301.0706, 285.0753, 263.0551, 245.0445, 177.0551
21	5.86	Hesperidin	C_28_H_34_O_15_	611.1970	611.1995	−4.09	[M+H]^+^	465.1351, 303.0872, 285.0754, 263.0553, 245.0448, 177.0550
22	6.35	Haplopine	C_13_H_11_NO_4_	246.0761	246.0768	−2.84	[M+H]^+^	231.0526, 216.0289, 188.0343, 160.0390
23	6.37	Isofagaridine	C_20_H_16_NO_4_^+^	344.1079	344.1069	2.91	[M]^+^	319.0849, 291.0897, 276.0663, 262.0865
24	6.60	Palmatine	C_21_H_22_NO_4_	352.1543	352.1555	−3.41	[M+H]^+^	336.1236, 320.1285, 292.0972, 190.0859
25	6.71	Sanguinarine A	C_20_H_14_NO_4_^+^	332.0917	332.0932	−4.52	[M]^+^	319.0842, 291.0892, 274.0870, 246.0915, 216.0808
26	6.77	Epiberberine	C_20_H_17_NO_4_	336.1230	336.1242	−3.57	[M+H]^+^	320.0919. 304.0968, 292.0970, 278.0814, 263.0936
27	7.16	Sanguinarine B	C_20_H_14_NO_4_^+^	332.0917	332.0934	−5.12	[M]^+^	319.0843, 291.0890, 274.0866, 246.0914, 216.0806
28	8.06	Marmesin	C_14_H_12_O_4_	245.0808	245.0813	−2.04	[M+H]^+^	212.0460, 191.0337, 163.0387, 147.0436, 128.0616
29	8.32	Nitidine A	C_21_H_18_NO_4_^+^	348.1230	348.1246	−4.60	[M]^+^	332.0926, 318.0763, 304.0974, 290.0818, 275.0939
30	8.71	Chelerythrine	C_21_H_17_NO_4_	348.1230	348.1240	−2.87	[M+H]^+^	332.0921, 318.0759, 304.0968, 290.0812
31	9.31	γ-Fagarine	C_13_H_11_NO_3_	230.0812	230.0821	−3.91	[M+H]^+^	215.0580, 200.0340, 186.0547, 172.0394, 158.0600
32	9.63	Skimmianine	C_14_H_13_NO_4_	260.0917	260.0925	−3.08	[M+H]^+^	227.0574, 200.0661, 199.0629, 184.0390, 170.0593
33	9.83	5-Methoxydictamnine	C_13_H_11_NO_3_	230.0812	230.0819	−3.04	[M+H]^+^	215.0579, 200.0340, 186.0547, 172.0398, 158.0594
34	9.86	Dictamnine	C_12_H_9_NO_2_	200.0706	200.0715	−4.50	[M+H]^+^	185.0474, 156.0446, 129.0576, 102.0466
35	10.16	Nitidine B	C_21_H_18_NO_4_^+^	348.1230	348.1240	−2.87	[M]^+^	332.0922, 318.0763, 304.0969, 290.0813, 275.0937
36	10.90	Unknown	C_15_H_14_O_5_	275.0914	275.0918	−1.45	[M+H]^+^	229.0493, 217.0497, 203.0338, 175.0390, 161.0596
37	12.03	Analog of magnoflorine	C_36_H_42_NO_9_^+^	632.2875	632.2870	0.79	[M]^+^	342.1700, 297.1124, 282.0890, 265.0862, 237.0912, 222.0673, 191.0852
38	15.13	Toddanone	C_16_H_18_O_5_	291.1227	291.1236	−3.09	[M+H]^+^	219.0652, 205.0496, 191.0702, 161.0597, 131.0488
39	16.42	Analog of jatrorrhizine	C_27_H_41_NO_13_	610.2470	610.2448	3.61	[M+Na]^+^	338.1390, 322.1077, 294.1123, 280.0973, 190.0862, 161.0595, 105.0700
40	16.79	Analog of nitidine	C_28_H_31_NO_6_^+^	477.2151	477.2150	0.21	[M]^+^	348.1234, 332.0927, 318.0769, 304.0975, 290.0818, 275.0936
41	18.46	Oxynitidine	C_21_H_17_NO_5_	364.1179	364.1192	−3.57	[M+H]^+^	348.0867, 334.0716, 320.0919, 306.0766, 290.0455, 278.0809
42	18.77	5,7-Dimethoxy-8-(3-methyl-2-butenoxy) coumarin	C_16_H_18_O_5_	291.1227	291.1233	−2.06	[M+H]^+^	220.0366, 193.0134, 178.0259, 165.0184, 133.0284, 107.0486
43	19.49	6-Ethoxychelerythrine	C_23_H_23_NO_5_	394.1654	394.1666	−3.04	[M+H]^+^	376.1548, 361.1313, 346.1093, 318.1125, 330.1127, 288.0795, 260.0829
44	20.16	Phellopterin	C_17_H_16_O_5_	301.1071	301.1081	−3.32	[M+H]^+^	218.0210, 190.0255, 162.0309, 134.0361, 106.0409
45	21.54	Suberosin	C_15_H_16_O_3_	245.1172	245.1181	−3.67	[M+H]^+^	215.0698, 187.0389, 175.0389, 143.0488, 131.0488
46	23.25	Dihydronitidine	C_21_H_19_NO_4_	350.1387	350.1399	−3.43	[M+H]^+^	334.1078, 319.0838, 290.0816, 247.0759, 219.0805
47	23.64	Dihydrochelerythrine	C_21_H_19_NO_4_	350.1387	350.1385	0.53	[M+H]^+^	334.1085, 319.0848, 290.0821, 247.0756, 219.0804
48	25.67	Analog of nitidine	C_36_H_32_NO_9_^+^	622.2072	622.2090	−2.89	[M]^+^	348.1230, 332.0920, 318.0760, 304.0969, 290.0814, 246.0912

### Characterization of Typical Benzophenanthroline

For benzophenanthrolines, their basic carbon framework consists of a benzene and a phenanthridine. Benzophenanthrolines are the most studied category of compounds in ZN, such as NC and chelerythrine (Li et al., 2017; Lin et al., 2020). In the MS/MS spectra of benzophenanthrolines, some certain product ions at *m/z* 332.09, 318.08, and 290.08 were presented, and consecutive losses of CH_2_ (14 Da), CO (28 Da), and O (16 Da) could be easily found. Nitidine ([M]^+^ at *m/z* 348.1246) is the typical compound, eluted at 8.71 min, and its molecular formula was determined as C_21_H_18_NO_4_^+^. In the positive MS/MS spectra, its MS/MS spectra had fragment peaks of 332.0926 [M-O]^+^ and 318.0763 [M-O-CH_2_]^+^, which correspond to the core substructures of benzophenanthrolines. Its tentative fragmentation pathway is shown in Supplementary Figures S1A,B. By integrating data inquiries, MDFSC and DFIBE approaches, nitidine, oxynitidine, and dihydronitidine were confirmed (Cesar et al., 2015).

### Characterization of Typical Quinoline

Quinoline consists of a benzene connecting to pyridine and other groups. The product ions of [M+H−14]^+^, [M+H−30]^+^, and [M+H−16]^+^ were available in the MS/MS spectra of quinoline, and certain product ions at *m/z* 200.03, 184.04 were the basis of DFIBE judgment. According to the patterns above, skimmianine ([M+H]^+^ at *m/z* 260.0925) was identified rapidly. It was eluted at 9.63 min, and its molecular formula was determined as C_14_H_13_NO_4_. It had fragment peaks of 200.0661[M+H-CH_2_+H_2_-O+H_2_-CH_2_O]^+^ and 184.0390 [M+H-CH_2_+H_2_-O+H_2_-CH_2_O-CH_2_+H_2_]^+^, which were equal to the core substructures. Then the skimmianine was identified with the support of integrating data bases (Wang, 1980), and its tentative fragmentation pathway is shown in Figures 3A,B. Besides, γ-fagarine and dictamnine are inferred as quinoline alkaloids.

### Characterization of Typical Isoquinoline

For isoquinoline, the basic carbon framework consists of a phenanthridine connecting to pyridine. Its MS/MS spectra provided several certain product ions at *m/z* 322.10, 290.09, and 107.04, and consecutive losses of CH_2_ (14 Da), C (12 Da), CO (28 Da), and O (16 Da) could be easily found (Xiao et al., 2018). The typical compound is jatrorrhizine ([M+H]^+^ at *m/z* 338.1400), which was eluted at 5.47 min, and its main molecular formula was determined as C_20_H_20_NO_4_^+^. Its fragment peaks contain 322.1091 [M+H-CH_4_]^+^, 294.1130 [M+H-CH_4_-CO]^+^, and 280.0972 [M+H-CH_4_-CO-CH_2_]^+^. After integrating data bases, jatrorrhizine was confirmed. During the whole characterization of isoquinoline, we found that the overall structure and fragmentation pathway of magnoflorine is very interesting. Tentative fragmentation pathways of jatrorrhizine and magnoflorine are shown in Figures 3C–F.

### Characterization of Typical Alkaloid Glycosides

With the assistance of MDFSC and DFIBE approaches, several alkaloid glycosides and alkaloids of larger relative molecular weights were found in ZN. It is attractive that the mother nucleuses of these compounds are similar to the known alkaloids we discovered. Take compound 40, for example; its mother nucleus is nitidine, which is described in the previous section, and its tentative fragmentation pathway is shown in Supplementary Figures S1A,B, and the tentative fragmentation pathways of oxynitidine and dihydronitidine are shown in Supplementary Figures S1C–F. Unfortunately, we could not suspect the accurate structure of these compounds and the attached groups.

### Characterization of Other Types of Compounds

Several alkaloids do not belong to the above four categories or cannot be found in medium to low polarity, such as magnocurarine B ([M]^+^ at *m/z* 314.1760). It is a benzylisoquinoline alkaloid, and is eluted at 3.14 min in high polarity. Its fragment peaks contain 269.1174 [M+H-C_2_H_8_N]^+^, 237.0909 [M+H-C_3_H_12_NO]^+^, 107.0489 [M+H-C_12_H_18_NO_2_]^+^, etc. After integrating the databases and DFIBE approach, magnocurarine B was identified. Its tentative fragmentation pathway is shown in Supplementary Figures S2A,B. This result indicates that the MDFSC and DFIBE approaches cannot only be applied in the characterization of alkaloids but also can be used in other types of compounds. Finally, the tentative fragmentation pathways of all remaining identified compounds are sorted out in Supplementary Figures S4–S45.

### Chromatographic Fingerprint Analysis of *Zanthoxylum nitidum* (Roxb.) DC. Samples

**FIGURE 4:**
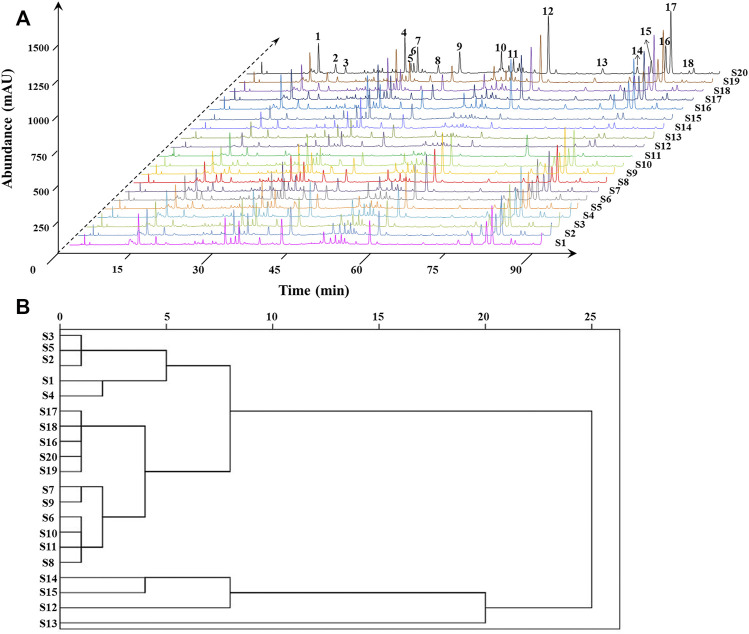
HPLC fingerprints and common peaks of 20 batches of ZN samples **(A)**. Cluster analysis of 20 batches of ZN samples **(B)**.

Under the optimized experimental conditions, the chromatographic fingerprints of ZN samples are neatly lined up in Figure 4. To evaluate the validation of the HPLC method, we selected and analyzed a random sample (S18) for its validation tests in terms of precision, stability, and repeatability. The RSDs of precision, stability, and repeatability were all less than 5% (shown in Supplementary Table S3), which indicated that the established method was reliable and repeatable.

Then the fingerprint of 20 batches of ZN samples was generated automatically by the software Similarity Evaluation System for Chromatographic Fingerprint of Traditional Chinese Medicine (2012A Version, Committee of Chinese Pharmacopeia). HPLC fingerprints were provided in Figure 4A, and reference spectra are shown in Supplementary Figure S3, respectively. Eighteen common characteristic peaks were chosen as research object, which covered more than 80% of the whole peak areas in the fingerprint. Subsequently, we performed the similarity analysis to evaluate the similarity of all the chromatographic profile of the samples, which was based on vector cosine calculations. It could be noted that most of similarity values were higher than 0.90, while similarity values of several batches from Guangdong province were 0.81–0.89 (Supplementary Table S1). The results showed that the chemical fluctuation among the random samples is pretty small in major producing areas (Sun and Liu, 2007), and it proved that the established fingerprint was reliable.

### Hierarchical Cluster Analysis of *Zanthoxylum nitidum* (Roxb.) DC. Samples

Based on the SPSS 19.0 software, we took the relative area of each common peak as the index for HCA (Liu et al., 2016). The sample similarity was measured by correlation coefficient as distance. The results are shown in Figure 4B, and 20 batches of ZN samples could be grouped into six categories: S1–S5, S6–S11 and S16–S20, S14–S15, S12, and S13 were, respectively, classified to one group. More importantly, the six groups belonged to the four resource provinces, and the group contains S16–S20 was from Guangxi with good similarity and intragroup similarity. The results indicated that ZN from different areas have good stability.

### Bioactivities of *Zanthoxylum nitidum* (Roxb.) DC. Samples

#### Anti-Inflammatory Activity of *Zanthoxylum nitidum* (Roxb.) DC. Samples

**FIGURE 5:**
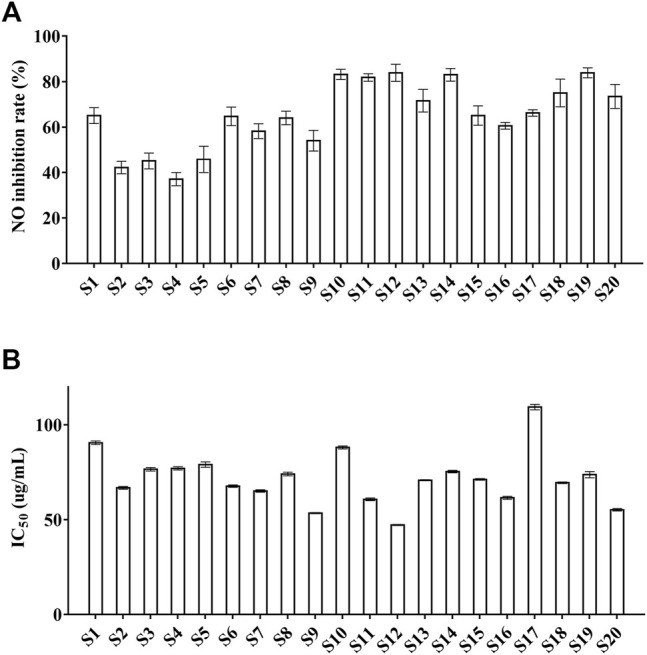
The NO inhibition and the antioxidant activities of 20 batches of ZN samples. **(A)** NO inhibition; **(B)** DPPH. Values represent means ± SD, *n* = 5 **(A)**; *n* = 3 **(B)**.

The inhibition of NO production is positively correlated with the anti-inflammatory activity (Chen et al., 2019). Thus, the inhibition of NO production was set as the index to assess the anti-inflammatory property of ZN samples in the present study. The results showed that cell viability (Supplementary Table S4) was from 77.4 ± 2.4 to 107 ± 1.3%, which means ZN inhibits the production of NO while not producing a toxic effect *in vitro*. Moreover, as is shown in Supplementary Table S4, the range of NO inhibition rate was 37.1 ± 2.9 to 83.9 ± 2.2%, and S10 (83.2 ± 2.3%), S11 (81.8 ± 1.7%), S12 (83.9 ± 3.8%), and S19 (83.9 ± 2.2%) were higher than 80% (Figure 5A). Among them, the sample S19 from Guangxi province showed best inhibition of NO production (83.9 ± 2.2%). The data indicated that most batches of ZN samples from different areas showed better anti-inflammatory activity.

### Antioxidant Activity of *Zanthoxylum nitidum* (Roxb.) DC. Samples

The IC_50_ value is the indicator to evaluate the antioxidant property of ZN samples, and it is negatively correlated with the antioxidant activity (Wang et al., 2017). As was shown in Supplementary Table S5, the IC_50_ values were from 47.21 ± 0.33 to 109.34 ± 1.43 μg/ml, and S10 (53.48 ± 0.29 μg/ml), S12 (47.21 ± 0.33 μg/ml), and S20 (55.22 ± 0.64 μg/ml) were lower than 60 μg/ml (Figure 5B). Besides, the sample S12, S20 showed strong antioxidant activity (47.21 ± 0.33 μg/ml; 55.22 ± 0.64 μg/ml), whereas the antioxidant activity of S17 (109.36 ± 1.43 μg/ml) was weak. The results revealed that most batches of ZN samples have better antioxidant activity.

### Discovery of Principal Bioactive Components by Partial Least Squares Regression, Gray Relational Analysis, and Spearman’s Rank Correlation Coefficient

In the past decades, artemisinin and ephedrine were regarded as gifts that the TCMs bring to the world (Tu, 2016; Leung and Xu, 2020). They were both isolated substances from TCMs and have undergone the modernization of traditional medicines. It proved that we can clarify the effectiveness of TCMs by finding out the bioactive components, but this is desperately not enough; the efficacy of TCMs does not come from a single compound, and TCMs usually consists of hundreds of components (Ma et al., 2016; Zhang et al., 2019). Thus, the search of the biological constituents of TCMs are a complex task. To solve this problem, the spectrum-effect relationship analysis might be a suitable choice. In this study, stoichiometric methods (PLSR, GRA, and SRCC) were applied to rapidly discover bioactive compounds from ZN samples.

**FIGURE 6:**
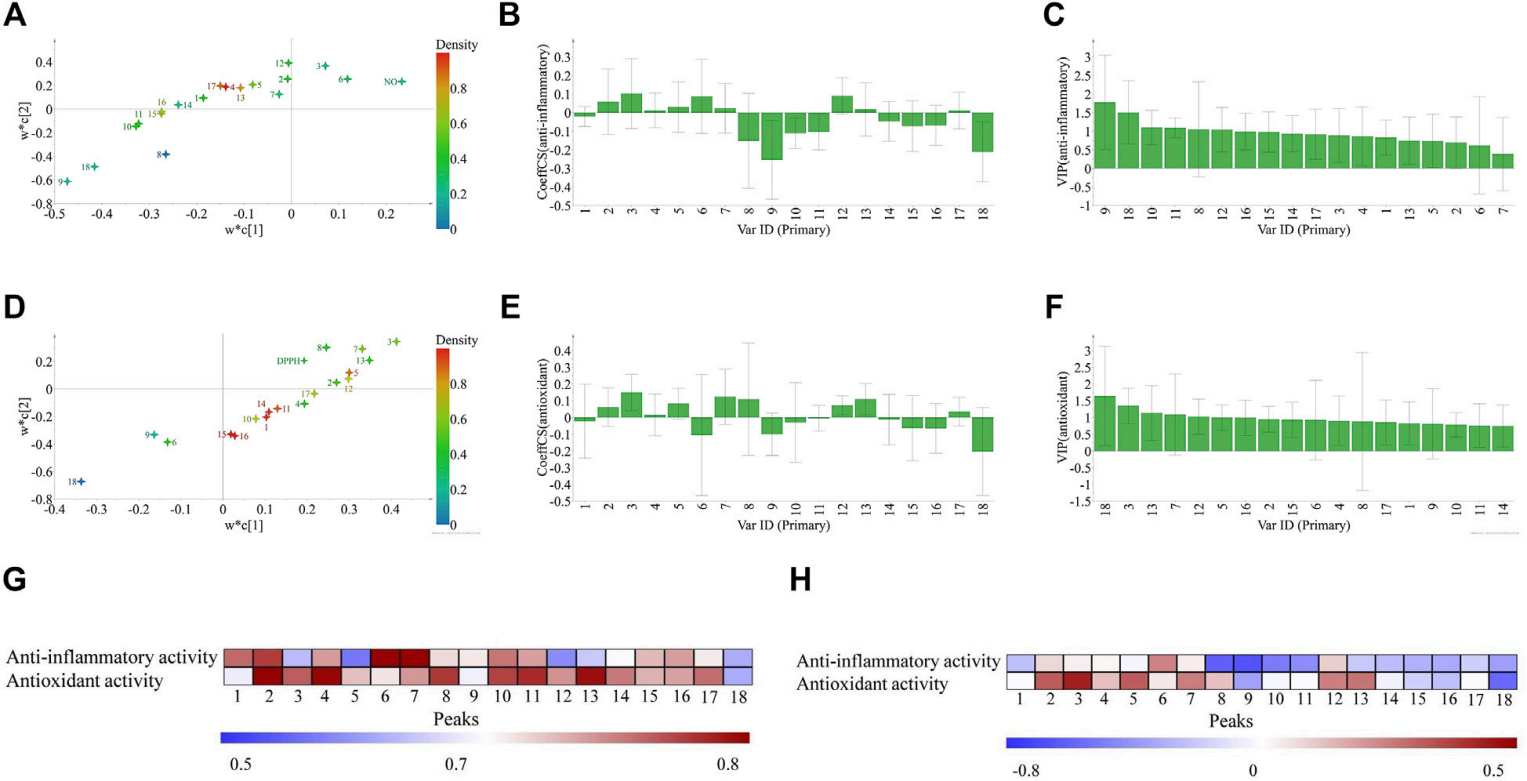
The three pharmacodynamic models to discover principal bioactive components. The results of partial least squares regression (PLSR, **A–F**): the loading scatters of PLSR **(A**: anti-inflammatory; **D**: antioxidant, colored according to density**)**; regression coefficient between the 18 common peaks and the anti-inflammatory, antioxidant activities, respectively **(B**: anti-inflammatory; **E**: antioxidant**)**, and VIP values **(C**: anti-inflammatory; **F**: antioxidant**)**. The heat map of bioactivities of 18 compounds by GRA analysis **(G)** and Spearman's rank correlation coefficient (SRCC) **(H)** in ZN samples. Peaks 1–18 represent the common peaks.

First, the results of GRA showed that the GRC between the relative contents of 18 common peaks and anti-inflammatory activity were in the range of 0.5691–0.8139 (Figure 6G). Peaks 1, 2, 4, 6, 7, and 10 showed significant influence to anti-inflammatory activity of ZN, and peaks 16, 11, 15, 8, 9, 17 showed influence to anti-inflammatory activity of ZN. The contribution order of chromatographic peaks to the anti-inflammatory activity was P6 > P7 > P2 > P1 > P10 > P4 > P16 > P11 > P15 > P8 > P9 > P17. Similarly, GRC between the relative contents of 18 common peaks and antioxidant activity were in the range of 0.5908–0.8520 (Figure 6G). Peaks 2, 3, 4, 7, 11, 12, 13, 14, and 17 showed an important effect to antioxidant activity of ZN, and peaks 5, 10, 15, and 16 showed influence to antioxidant activity of ZN. The contribution order of chromatographic peaks to the antioxidant activity was P2 > P4 > P13 > P11 > P3 > P17 > P14 > P12 > P7 > P16 > P15 > P10 > P5.

Second, the PLSR model was used to discover the potential active compounds by correlating the fingerprint chromatographic data, antioxidant or anti-inflammatory activities. The X matrix of dimensions (20 × 18) from common peak areas and the Y matrix of DPPH and the NO inhibition activities were used in this model (Figure 5 and Supplementary Tables S4, S5. The *R* ^2^ of NO inhibition is 0.764, and *R* ^2^ of DPPH is 0.762, respectively). Then the PLSR loading scatters were performed (Figures 6A,D), where Y-variables are situated near the X-variables and positively correlated to them.

For the anti-inflammatory activity of ZN samples, we found that peaks 2, 3, 4, 5, 6, 7, 12, 13, and 17 were positively correlated to the anti-inflammatory activity (Figure 6B). Furthermore, we employed the parameter VIP to screen the variables responsible for the anti-inflammatory activity. Variables above the VIP value threshold of 1.0 were filtered out as candidate bioactive compounds. Among them, peaks 4 (nitidine), 12, and 17 were screened out and considered as the potential anti-inflammatory compounds of ZN (Figure 6C). For the antioxidant activity of ZN samples, similarly, peaks 3, 5 (chelerythrine), 7 (hesperidin), 12 (oxynitidine), and 13 appeared in a cluster trend. These compounds were positively correlated to the antioxidant activity, and the VIP value was above 1.0 (Figures 6E,F). According to the above results, we preliminarily inferred that the peaks 3, 4, 5, 7, 12, 13, and 17 were more relevant to the measured activities.

Finally, the results of SRCC (shown in Figure 6H) indicated that peaks 2, 3, 4, 6, 7, and 12 showed an influence to the anti-inflammatory activity of ZN, and peaks 2, 3, 5, 7, 12, and 13 have an important influence on the antioxidant activity of ZN.

By combining the results of PLSR, GRA, and SRCC, it can be concluded that peaks 4, 12, and 17 are tentatively assigned as candidate ingredients accounting for the anti-inflammatory activity, and peaks 3, 5, 7, 12, and 13 are the candidate ingredients for antioxidant activity. Among them, owning to the high content and bioactive activities in ZN, nitidine has attracted much attention. It has been found that nitidine could inhibit LPS-induced TNF alpha, IL-1beta, and IL-6 production by the inhibition of phosphorylation of MAPK in the RAW264.7 cell test (Wang et al., 2012). Taken together, nitidine might be the main anti-inflammatory component in ZN. What is more, peaks 12 and 17 might also be the potential anti-inflammatory compounds of ZN, which warrant further investigation. Among the antioxidant candidate compounds, hesperidin was identified to have an oxidative damage protective effect against γ-radiation-induced tissue damage in Sprague–Dawley rats (Pradeep et al., 2012). Additionally, peaks 3, 12, and 13 might also be the potential antioxidant compounds of ZN. As a complicated system, the potential synergistic and/or antagonism effects arising from multicomponents in ZN must be taken into consideration. Thus, it could be found that peaks 3, 4, 5, 7, 12, 13, and 17 might be the main bioactive compounds in ZN samples, and among them, peaks 4, 12, and 17 were the principal anti-inflammatory components and peaks 3, 5, 7, 12, and 13 were regarded as the principal antioxidant components, respectively.

### Verification of Bioactive Compound Activities

In order to confirm the reliability of the correlation analysis, the anti-inflammatory activity of nitidine and the antioxidant activity of chelerythrine and hesperidin were determined by the antioxidant and anti-inflammatory bioactivity assays. The result of nitidine (Supplementary Figure S46A) showed that the IC_50_ value of nitidine is 87.241 ± 1.752 μM, which indicated that nitidine might be the main anti-inflammatory component of ZN. The results of chelerythrine and hesperidin (Supplementary Figures S46B,C) showed that chelerythrine has an antioxidant activity, and hesperidin showed a weaker antioxidant activity, which indicated that chelerythrine and hesperidin might be the main antioxidant components of ZN. Besides, according to the results of the correlation analysis, it could be noticed that diosmin has no antioxidant activity, which is in accordance with the results of these previous studies (Khlebnikov et al., 2007; Naso et al., 2016), and all these results verified the reliability of the correlation analysis.

## Conclusion

In order to solve the challenge of discovering bioactive compounds in ZN, a comprehensive filtering approach and a spectrum–effect relationship were performed in our work. A total of 48 compounds were identified from ZN by the structure-diagnostic ion-oriented network, and 35 of them were alkaloids. During the identification of chemical components in ZN by integrating the MDFSC and DFIBE approaches, some new alkaloid analogs were found, such as compounds **37**, **39**, **40**, and **48**. Meanwhile, we discovered the principal bioactive components of ZN by using the GRA, PLSR, and SRCC models. Among the bioactive compounds, peaks **4** (nitidine), **12** (oxynitidine), and **17** might be the potential anti-inflammatory compounds in ZN, and peaks **3**, **5** (chelerythrine), **7** (hesperidin), **12**, and **13** might contribute to the antioxidant activity of ZN. Our study not only revealed the plant metabolites of anti-inflammatory and antioxidant activities of ZN, but it could also be an applicable data support for the discovery of active components of other TCMs. Our future studies will perform more comprehensive and reliable methods to verify the anti-inflammatory and antioxidant activities of ZN *in vitro* and *in vivo*.

## Data Availability

The original contributions presented in the study are included in the article/Supplementary Material, further inquiries can be directed to the corresponding authors.
