# Supplementary material for: Spectrum-Effect Relationship Analysis of Bioactive Compounds in Zanthoxylum nitidum (Roxb.) DC. by Ultra-High Performance Liquid Chromatography Mass Spectrometry Coupled With Comprehensive Filtering Approaches
Source: Front Pharmacol. 2022 Mar 9;13:794277. doi: 10.3389/fphar.2022.794277 (PMC8959880; doi:10.3389/fphar.2022.794277)
Supplement: Supplementary file 1 [file DataSheet1.DOCX]

**Supplementary Data**

**Spectrum-effect Relationship Analysis of Bioactive Activities with** ***Zanthoxylum nitidum* (Roxb.) DC. by UPLC-MS/MS and Comprehensive Filtering Approaches**

**Si-wei Rao^a,1^, Yuan-yuan Duan^a,1^, Han-qing Pang^b^,** **Shao-hua Xu^a^, Shou-qian Hu^a^, Ke-guang Cheng^a^, Dong Liang^a*^, Wei Shi^a*^**

**^a^** State Key Laboratory for Chemistry and Molecular Engineering of Medicinal Resources, Collaborative Innovation Center for Guangxi Ethnic Medicine, School of Chemistry and Pharmaceutical Science, Guangxi Normal University, Guilin 541004, PR China

**^b^** Yangzhou University, 180 Siwangting Road, Yangzhou, Jiangsu 225002, PR China

***Corresponding Authors:** State Key Laboratory for the Chemistry and Molecular Engineering of Medicinal Resources, Guangxi Normal University, No. 15 Yucai Road, Guilin 541004, China.

E-mail: liangdonggxnu@163.com.

E-mail: swv2012@163.com.

**TABLE S1.** The source and similarities of 20 ZN samples.

| **NO.** | **Source** | **Similarity** | **NO.** | **Source** | **Similarity** |
| --- | --- | --- | --- | --- | --- |
| **S1** | Yunnan | 0.988 | **S11** | Hunan | 0.989 |
| **S2** | Yunnan | 0.954 | **S12** | Guangdong | 0.885 |
| **S3** | Yunnan | 0.959 | **S13** | Guangdong | 0.831 |
| **S4** | Yunnan | 0.982 | **S14** | Guangdong | 0.889 |
| **S5** | Yunnan | 0.956 | **S15** | Guangdong | 0.810 |
| **S6** | Hunan | 0.994 | **S16** | Guangxi | 0.976 |
| **S7** | Hunan | 0.988 | **S17** | Guangxi | 0.976 |
| **S8** | Hunan | 0.985 | **S18** | Guangxi | 0.975 |
| **S9** | Hunan | 0.982 | **S19** | Guangxi | 0.960 |
| **S10** | Hunan | 0.987 | **S20** | Guangxi | 0.963 |

| **Time (min)** | **Acetonitrile (%)** | **0.1% Formic acid (%)** | **Time (min)** | **Acetonitrile (%)** | **0.1% Formic acid (%)** |
| --- | --- | --- | --- | --- | --- |
| **0** | 5 | 95 | **50** | 44 | 56 |
| **5** | 9 | 91 | **60** | 44 | 56 |
| **13** | 13 | 87 | **75** | 60 | 40 |
| **20** | 20 | 80 | **77** | 64 | 36 |
| **25** | 23 | 77 | **85** | 75 | 25 |
| **35** | 26 | 74 | **87** | 85 | 15 |
| **45** | 42 | 58 | **90** | 92 | 8 |

**TABLE S2.** The mobile phase system of HPLC fingerprint analysis.

**TABLE S3.** The precision, repeatability and stability of the 18 common peaks (P1-P18) in ZN samples.

| **Peak** | **Precision**  **RSD (%) (n=6)** | **Repeatability RSD (%) (n=6)** | **Stability**  **RSD (%)** |
| --- | --- | --- | --- |
| **P1** | 1.73 | 4.58 | 3.13 |
| **P2** | 2.31 | 3.58 | 0.98 |
| **P3** | 4.14 | 2.08 | 2.28 |
| **P4** | 1.39 | 3.43 | 1.21 |
| **P5** | 2.44 | 3.05 | 5.16 |
| **P6** | 1.79 | 2.46 | 1.66 |
| **P7** | 1.54 | 2.06 | 1.12 |
| **P8** | 3.17 | 3.08 | 2.67 |
| **P9** | 1.85 | 3.17 | 5.22 |
| **P10** | 1.23 | 2.10 | 1.07 |
| **P11** | 1.60 | 0.93 | 2.74 |
| **P12** | 0.00 | 1.58 | 0.00 |
| **P13** | 1.12 | 0.00 | 3.78 |
| **P14** | 1.01 | 1.91 | 2.16 |
| **P15** | 0.95 | 2.38 | 3.81 |
| **P16** | 1.06 | 1.41 | 2.11 |
| **P17** | 4.08 | 1.35 | 1.33 |
| **P18** | 0.96 | 1.79 | 4.96 |


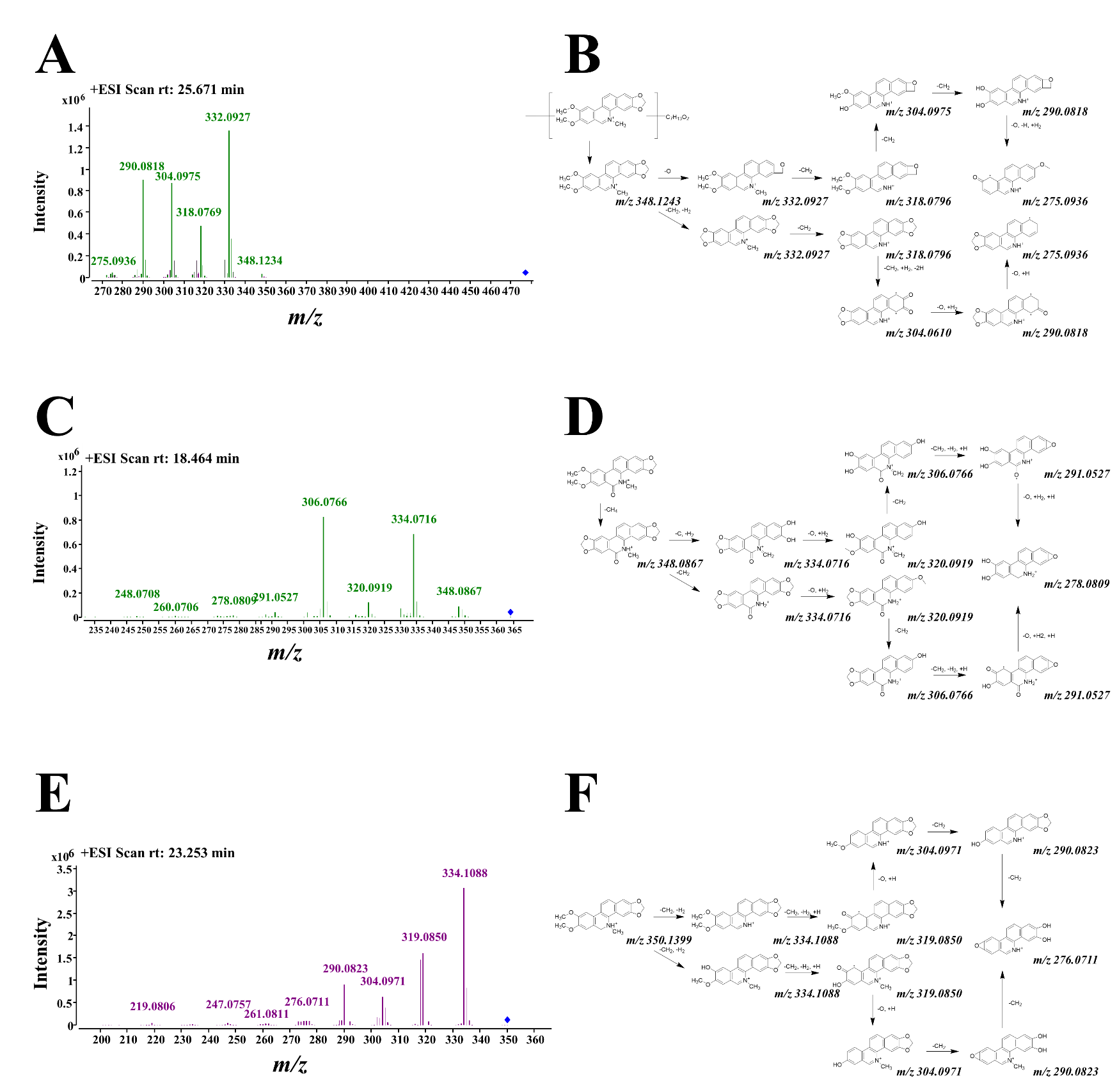


6

x10

0

0.2

0.4

0.6

0.8

1

1.2

1.4

332.0927

290.0818

318.0769

275.0936

348.1234

270

280

290

300

310

320

330

340

350

360

370

380

390

400

410

420

430

440

450

460

470

***m/z***

304.0975

**Intensity**

***m/z 348.1243***

***m/z 332.0927***

***m/z 332.0927***

***m/z 318.0796***

***m/z 318.0796***

***m/z 304.0975***

***m/z 290.0818***

***m/z 275.0936***

***m/z 275.0936***

***m/z 290.0818***

***m/z 304.0610***

A

B

**FIGURE S1** Tentative fragmentation pathway and mass spectrogram of alkaloid glycoside whose mother nucleus is nitidine in ZN sample (A-B). Tentative fragmentation pathway and mass spectrogram of oxynitidine and dihydronitidine (C-F).


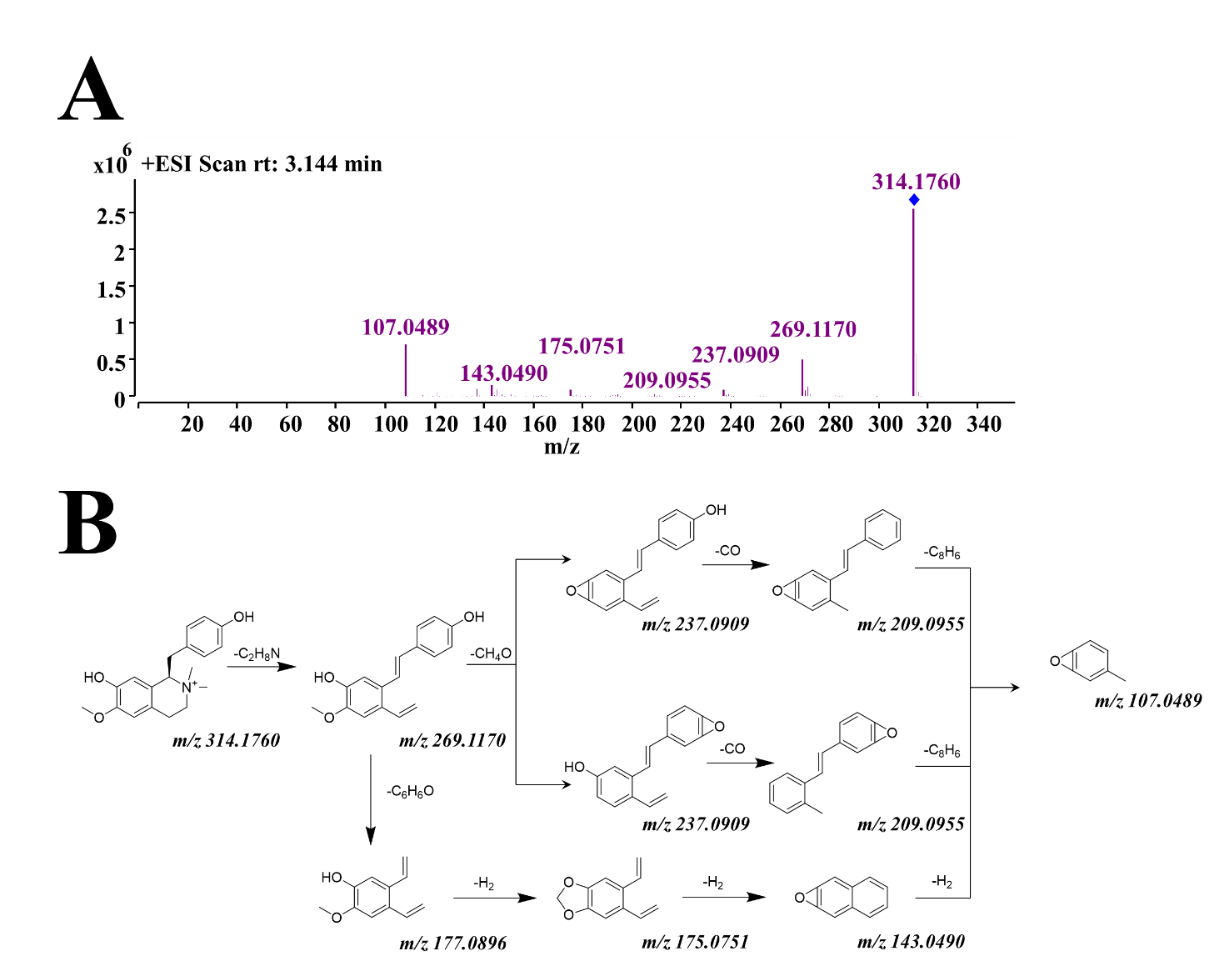


FIGURE S2 Tentative fragmentation pathway and mass spectrogram of magnocurarine B. A: the mass spectrogram; B: the tentative fragmentation pathway.

**TABLE S4.** The inhibition rates of NO production of ZN samples.

| **Sample number** | **Cell viability (%)** | **NO inhibition rate (%)** | **Sample number** | **Cell viability (%)** | **NO inhibition rate (%)** |
| --- | --- | --- | --- | --- | --- |
| **S1** | 94.0±3.3 | 65.1±3.5 | **S11** | 82.4±2.5 | 81.8±1.7 |
| **S2** | 102.7±4.0 | 42.2±2.7 | **S12** | 107.0±1.3 | 83.9±3.8 |
| **S3** | 100.2±2.5 | 45.1±3.5 | **S13** | 87.5±0.5 | 71.6±5.0 |
| **S4** | 97.4±2.9 | 37.1±2.9 | **S14** | 82.2±2.7 | 83.0±2.8 |
| **S5** | 85.9±1.4 | 45.8±5.8 | **S15** | 90.2±1.5 | 65.1±4.3 |
| **S6** | 96.9±1.8 | 64.7±4.1 | **S16** | 91.5±1.5 | 60.6±1.5 |
| **S7** | 94.7±2.3 | 58.2±3.3 | **S17** | 77.4±2.4 | 66.2±1.4 |
| **S8** | 96.8±3.0 | 64.0±3.0 | **S18** | 81.8±2.3 | 75.0±6.1 |
| **S9** | 94.8±1.8 | 54.0±4.6 | **S19** | 81.2±2.0 | 83.9±2.2 |
| **S10** | 92.6±3.2 | 83.2±2.3 | **S20** | 101.5±1.6 | 73.5±5.3 |

**TABLE S5.** The antioxidant activity (IC_50_ of DPPH) of ZN samples.

| **Sample number** | **IC_50_ (ug/mL)** | **Sample number** | **IC_50_ (ug/mL)** |
| --- | --- | --- | --- |
| **S1** | 55.219±0.64 | **S11** | 60.809±0.73 |
| **S2** | 90.591±0.90 | **S12** | 47.211±0.33 |
| **S3** | 66.801±0.75 | **S13** | 70.822±0.32 |
| **S4** | 76.598±0.92 | **S14** | 75.375±0.65 |
| **S5** | 77.099±0.85 | **S15** | 71.269±0.42 |
| **S6** | 79.116±1.44 | **S16** | 61.565±0.78 |
| **S7** | 67.725±0.63 | **S17** | 109.364±1.43 |
| **S8** | 65.149±0.65 | **S18** | 69.54±0.43 |
| **S9** | 74.098±0.93 | **S19** | 73.774±1.67 |
| **S10** | 53.476±0.29 | **S20** | 55.219±0.64 |


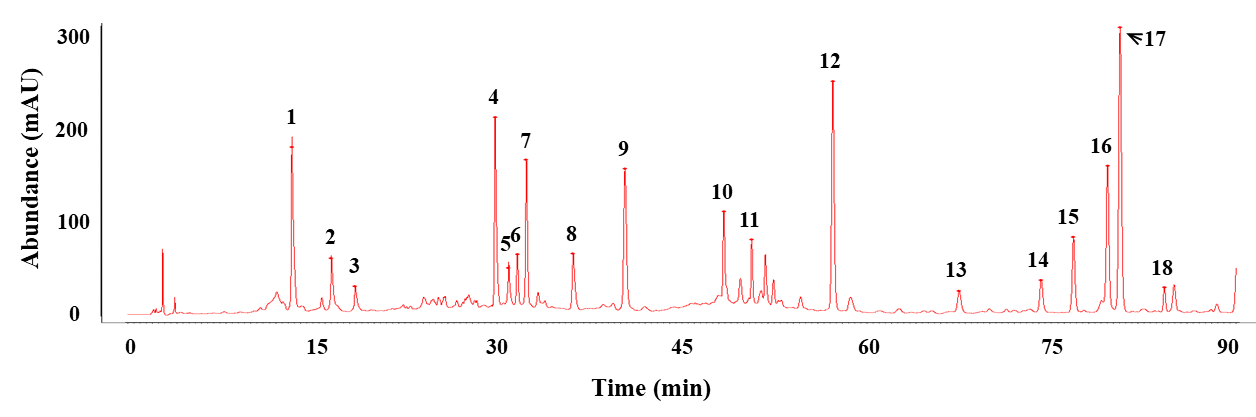


FIGURE S3 18 common characteristic peaks in reference spectra.


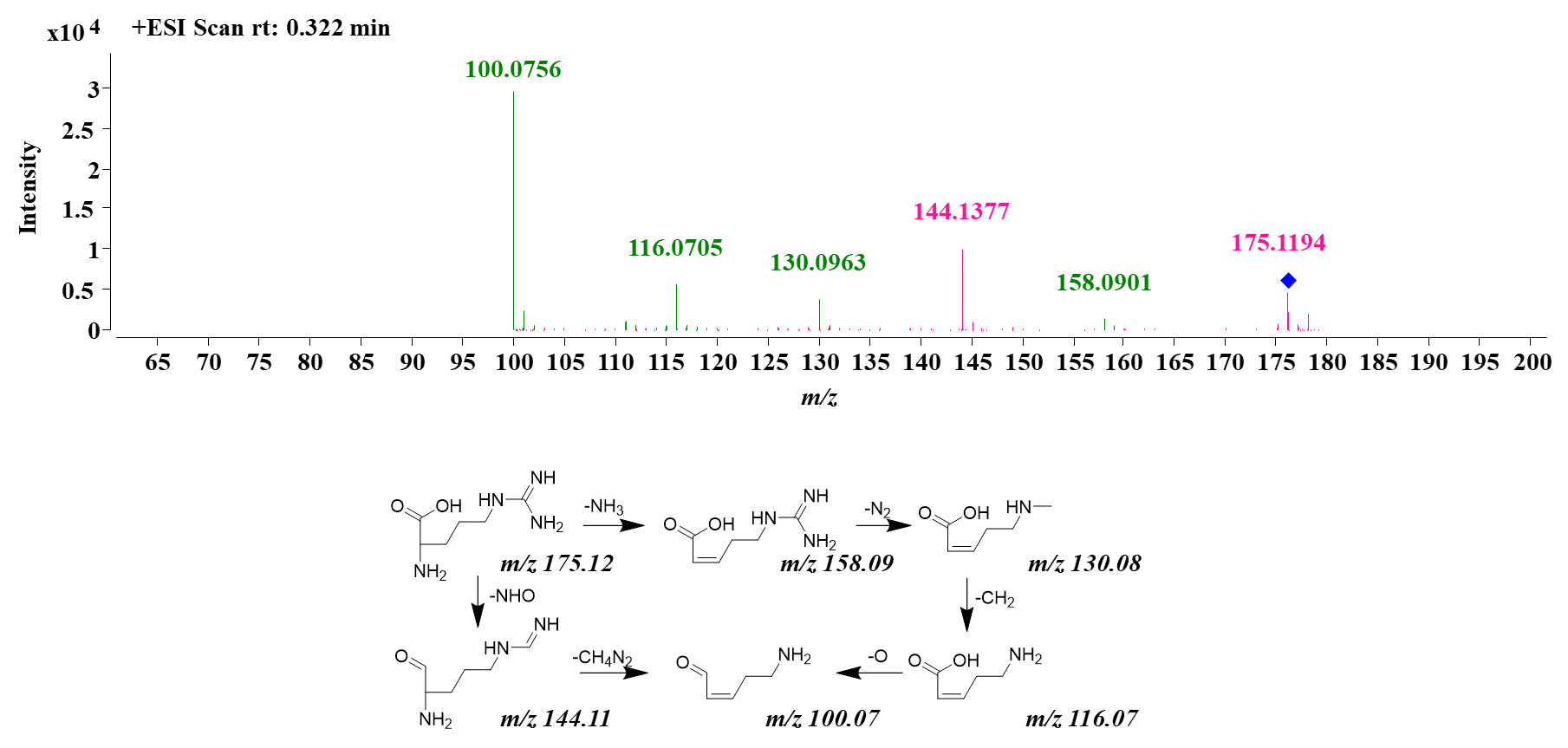


FIGURE S4 Tentative fragmentation pathway and mass spectrogram of compound 1 (L -Arginine).


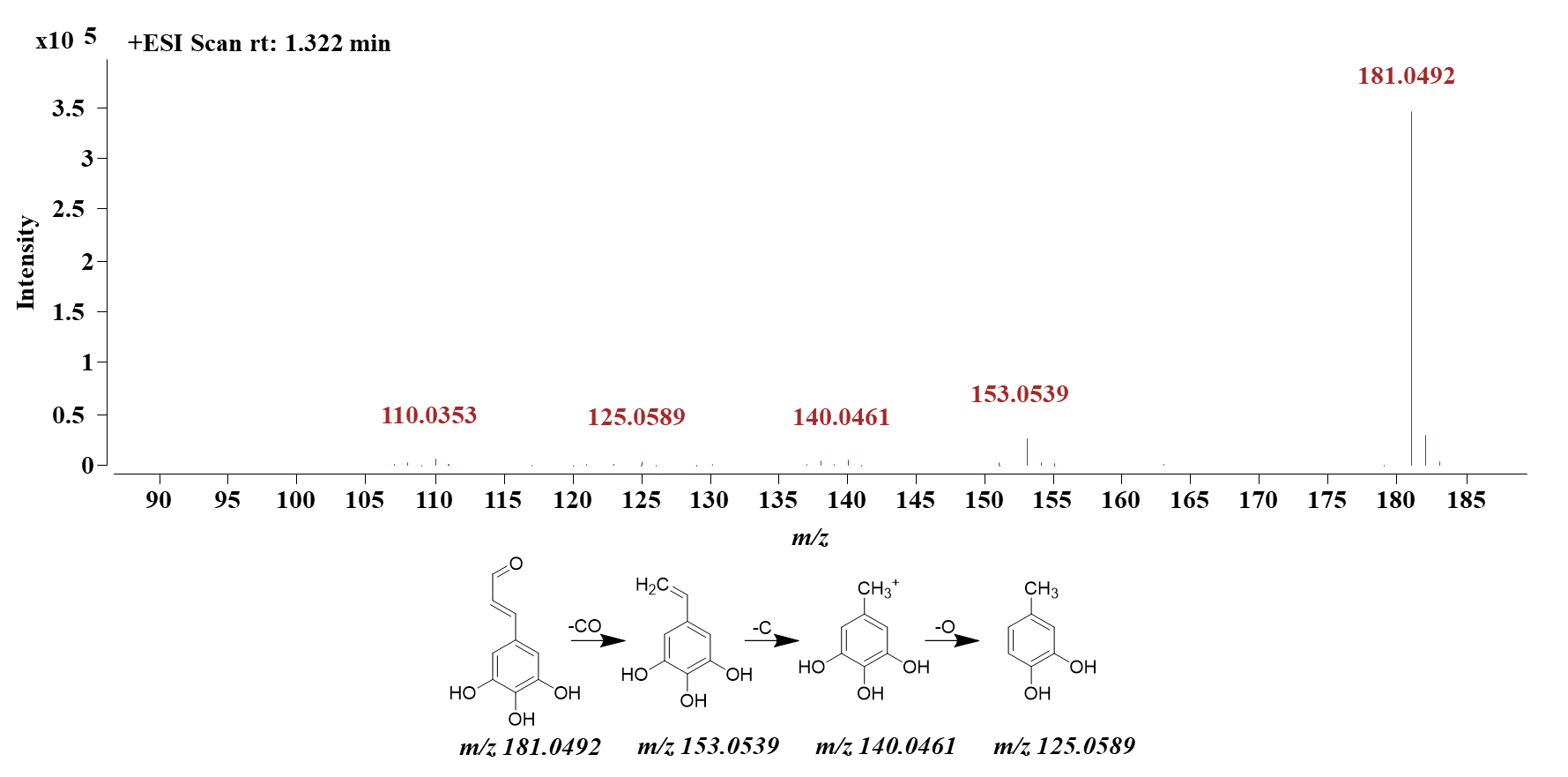


FIGURE S5 Tentative fragmentation pathway and mass spectrogram of compound 2 ((E)-3-(3,4,5-trihydroxyphenyl) acrylaldehyde).


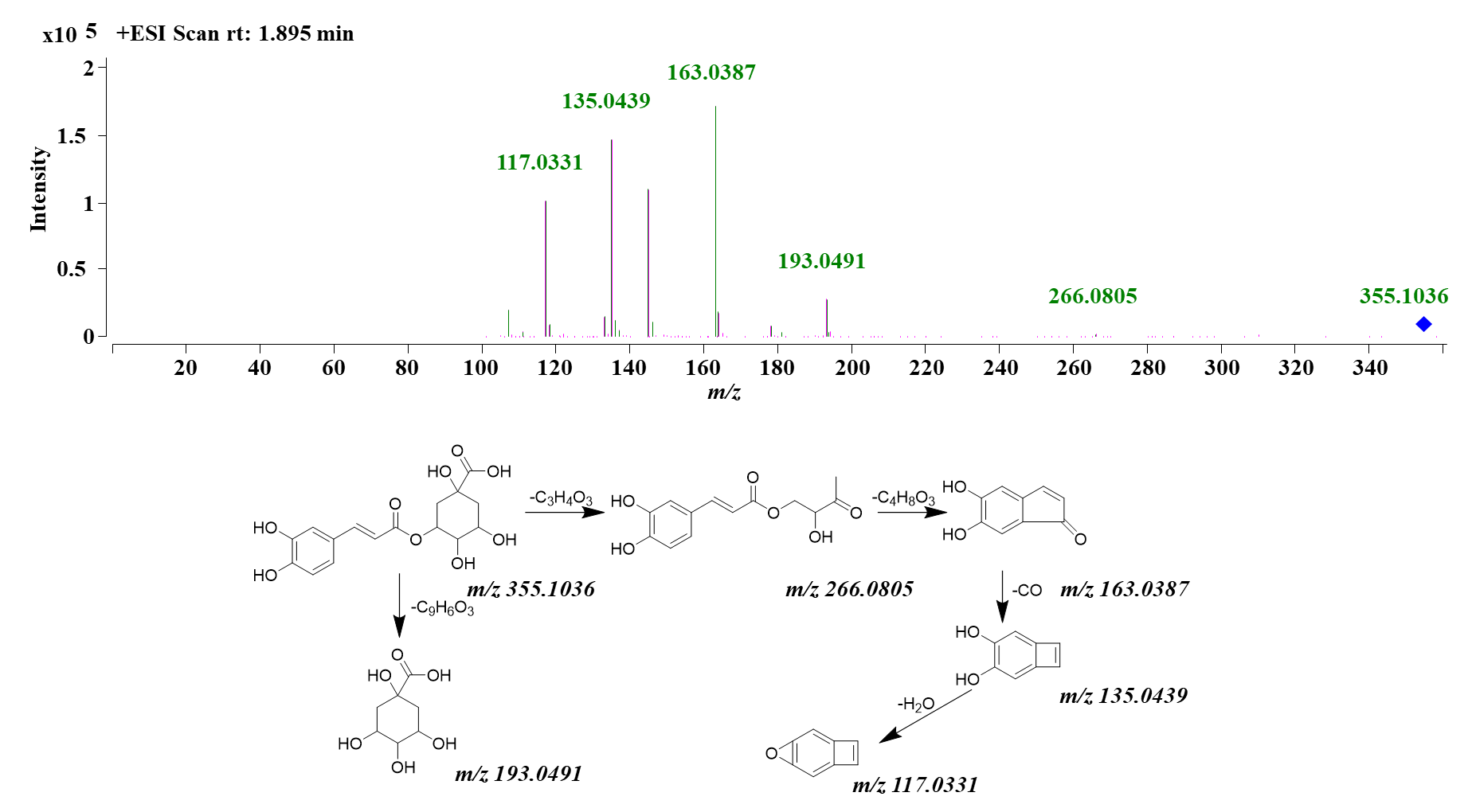


FIGURE S6 Tentative fragmentation pathway and mass spectrogram of compound 3 (Chlorogenic Acid).


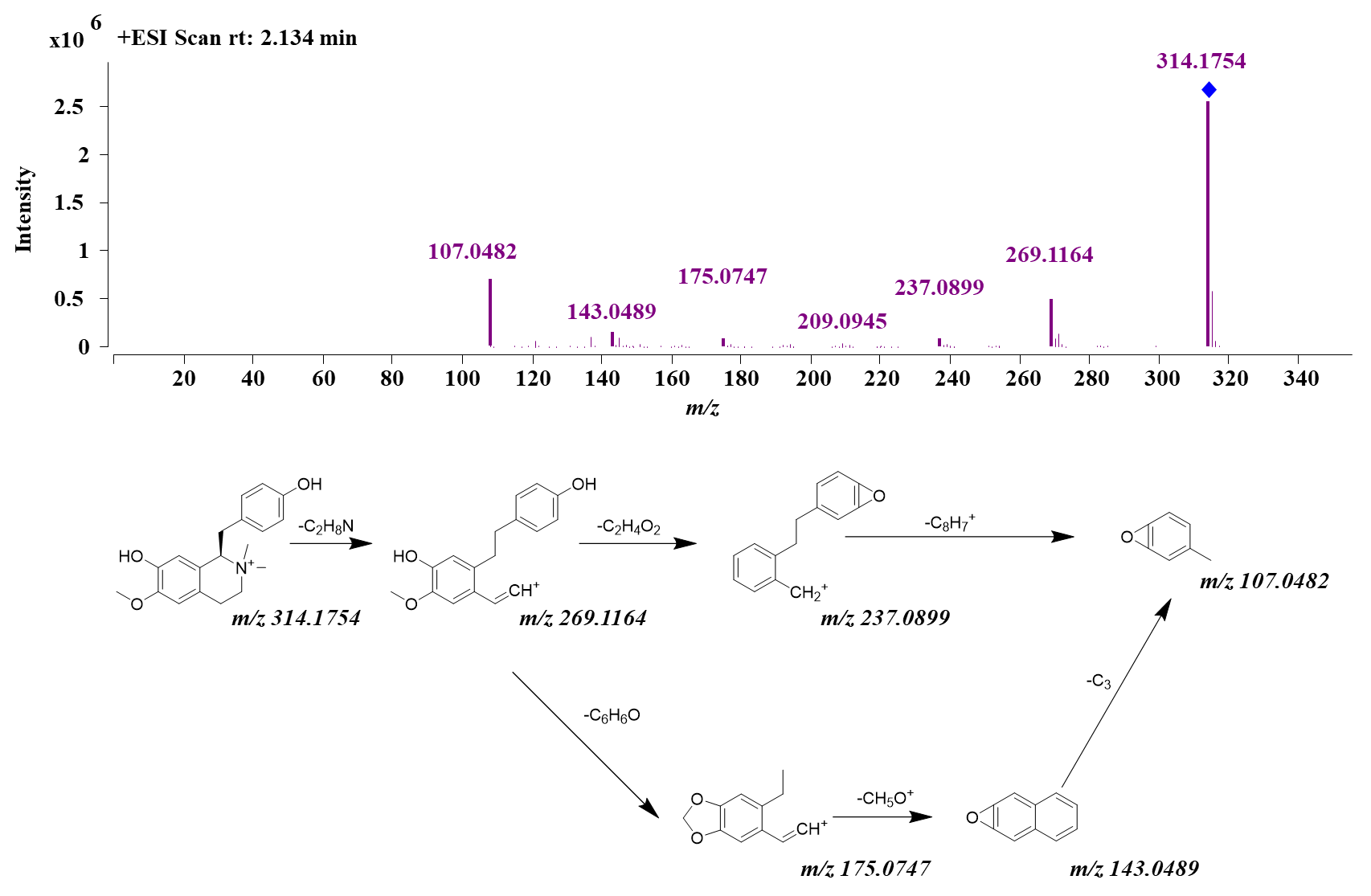
 FIGURE S7 Tentative fragmentation pathway and mass spectrogram of compound 4 (Magnocurarine A).


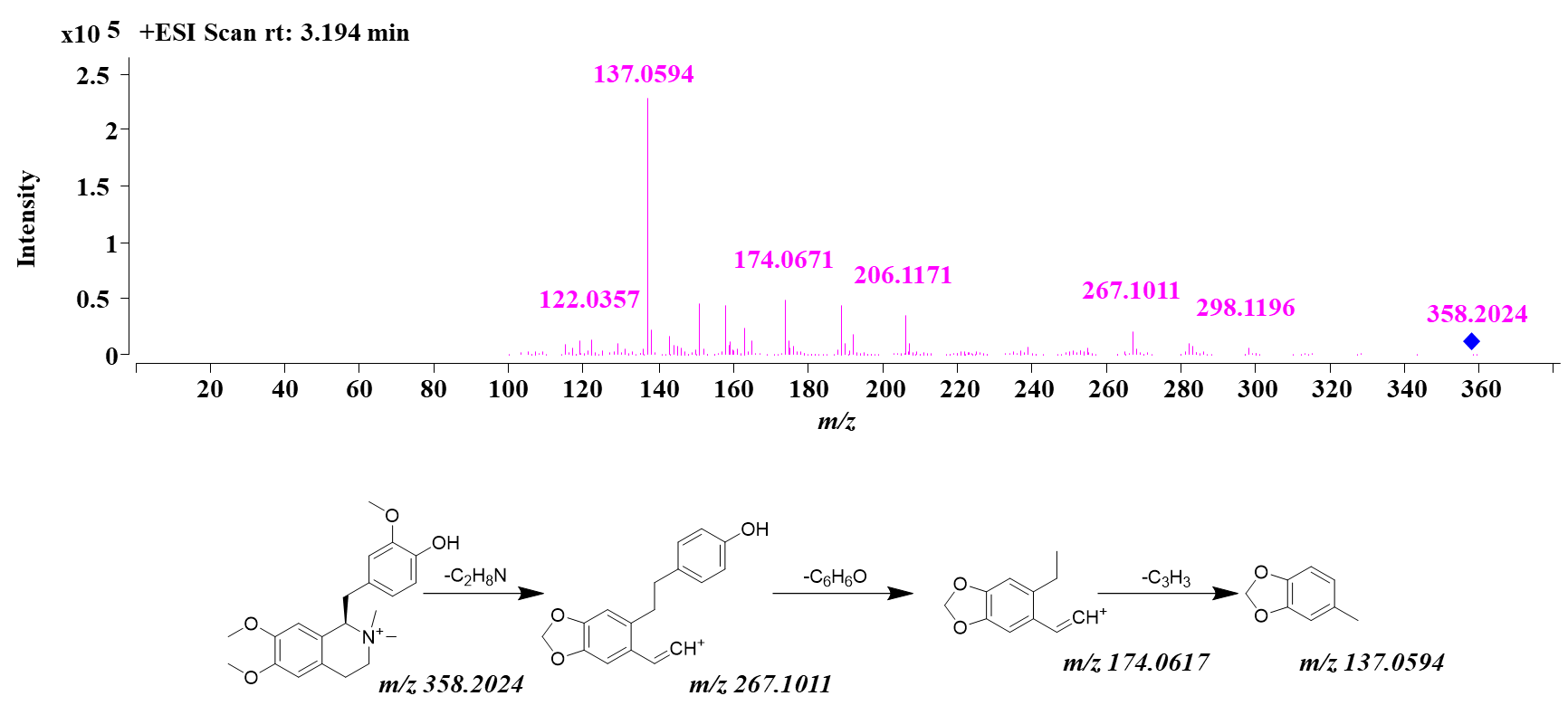
 FIGURE S8 Tentative fragmentation pathway and mass spectrogram of compound 7 (3'-hydroxy-4', 6, 7-trimethoxyl-N, N-dimethyltetrahydroisoquinoline).


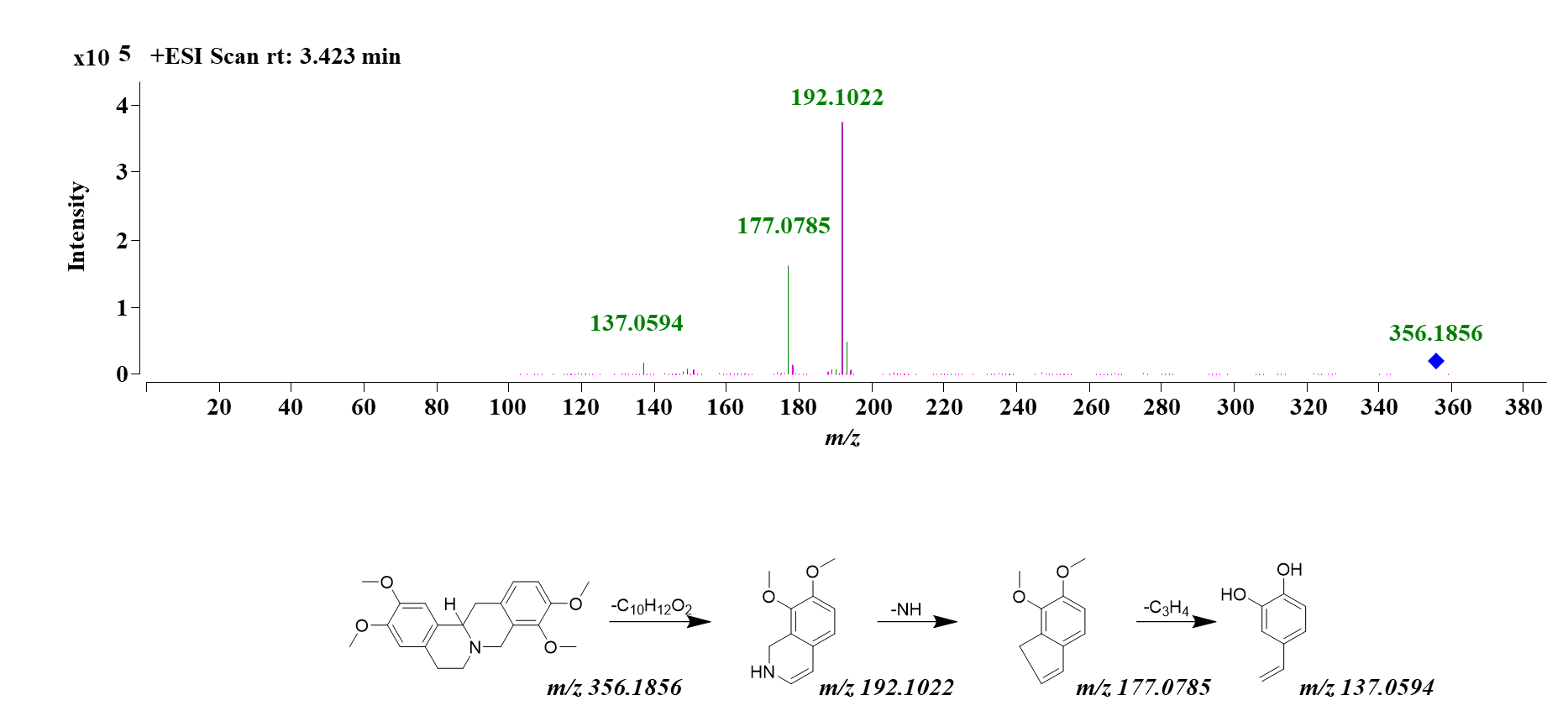
 FIGURE S9 Tentative fragmentation pathway and mass spectrogram of compound 8 (D-Tertrahydropaimatine).


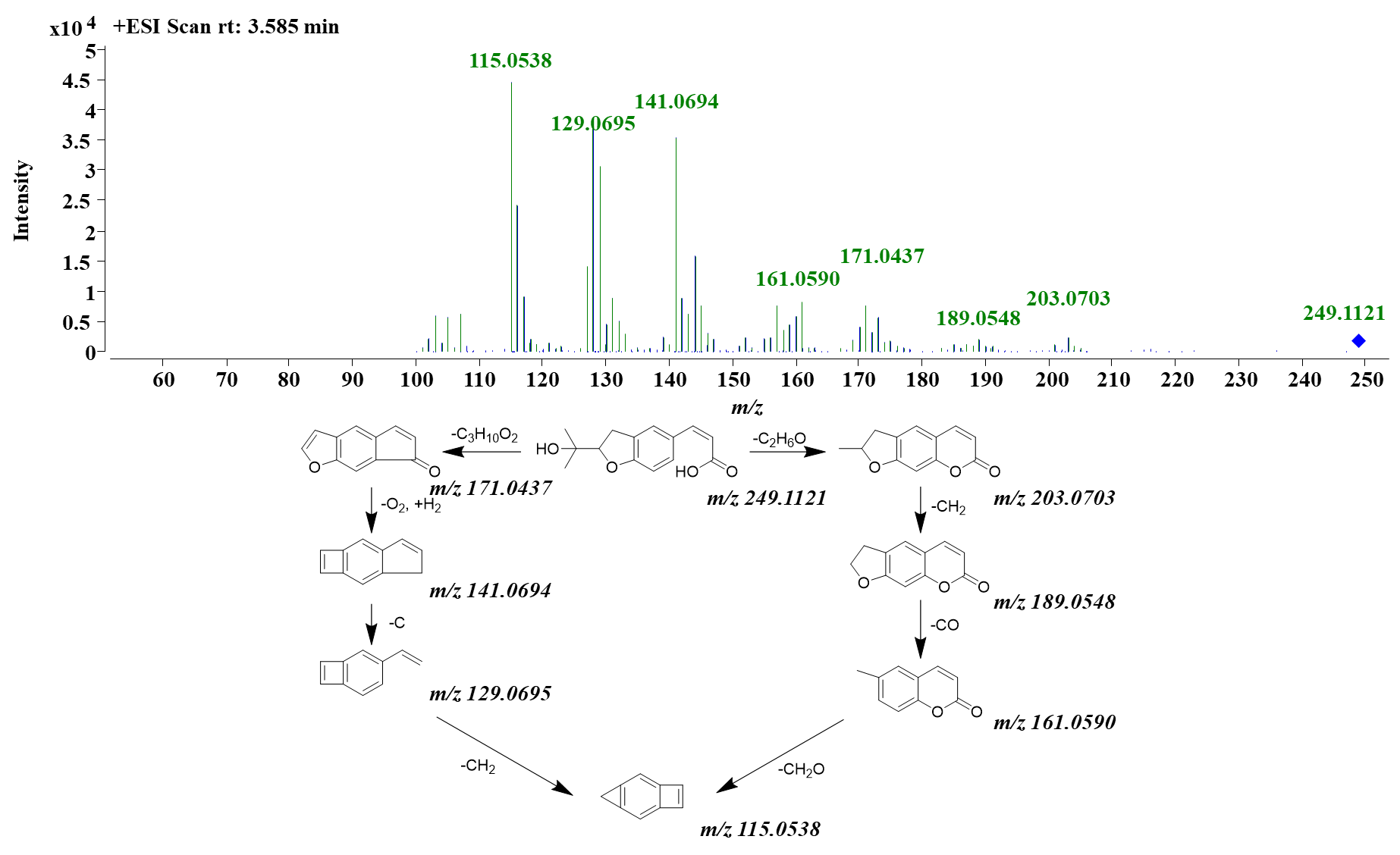
 FIGURE S10 Tentative fragmentation pathway and mass spectrogram of compound 9 ((Z)-3-(2-(2-hydroxypropan-2-yl)-2,3-dihydrobenzofuran-5-yl) acrylic acid).


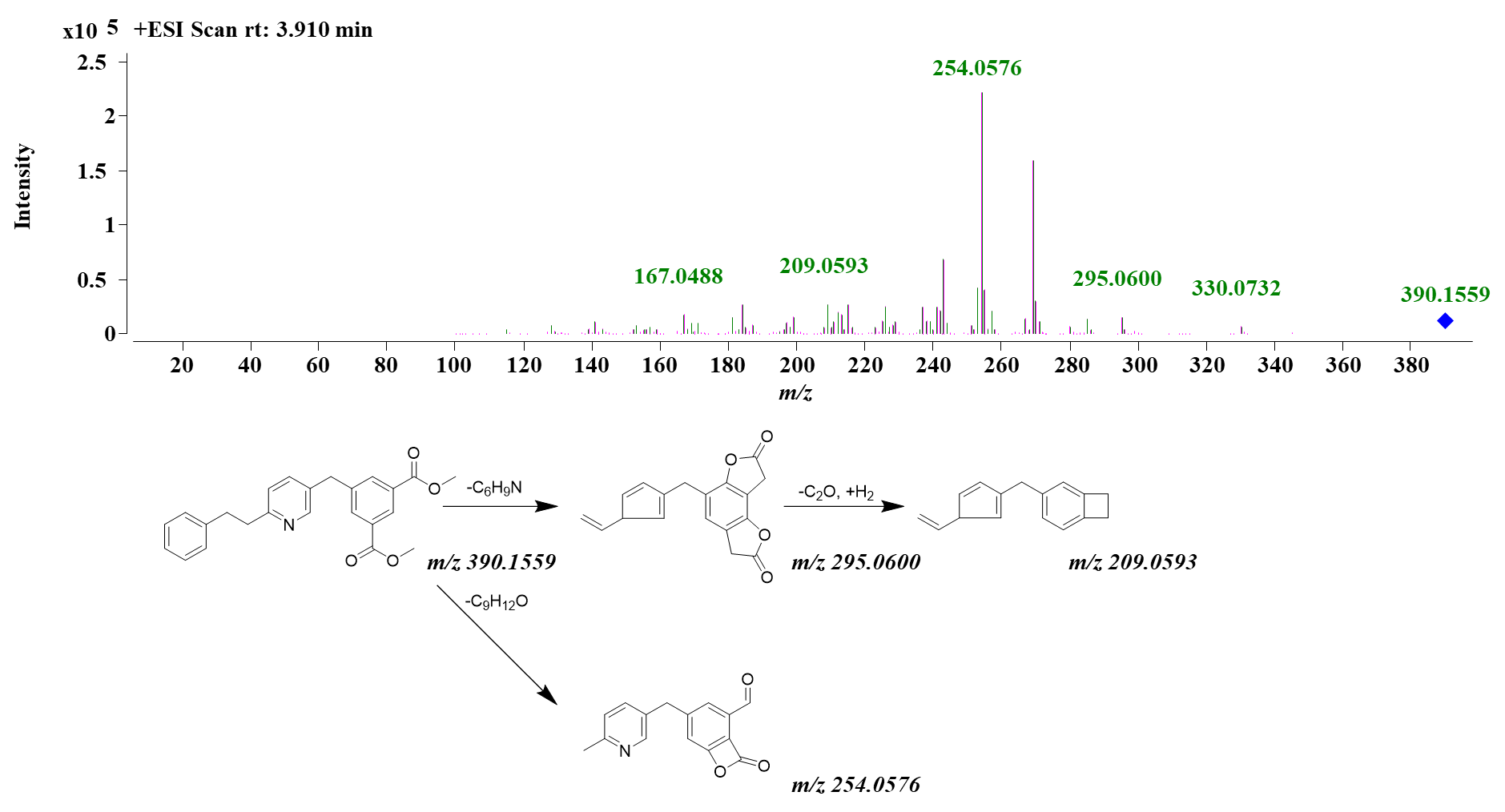
 FIGURE S11 Tentative fragmentation pathway and mass spectrogram of compound 10 (Dimethyl 5-((6-phenethylpyridin-3-yl) methyl) isophthalate).


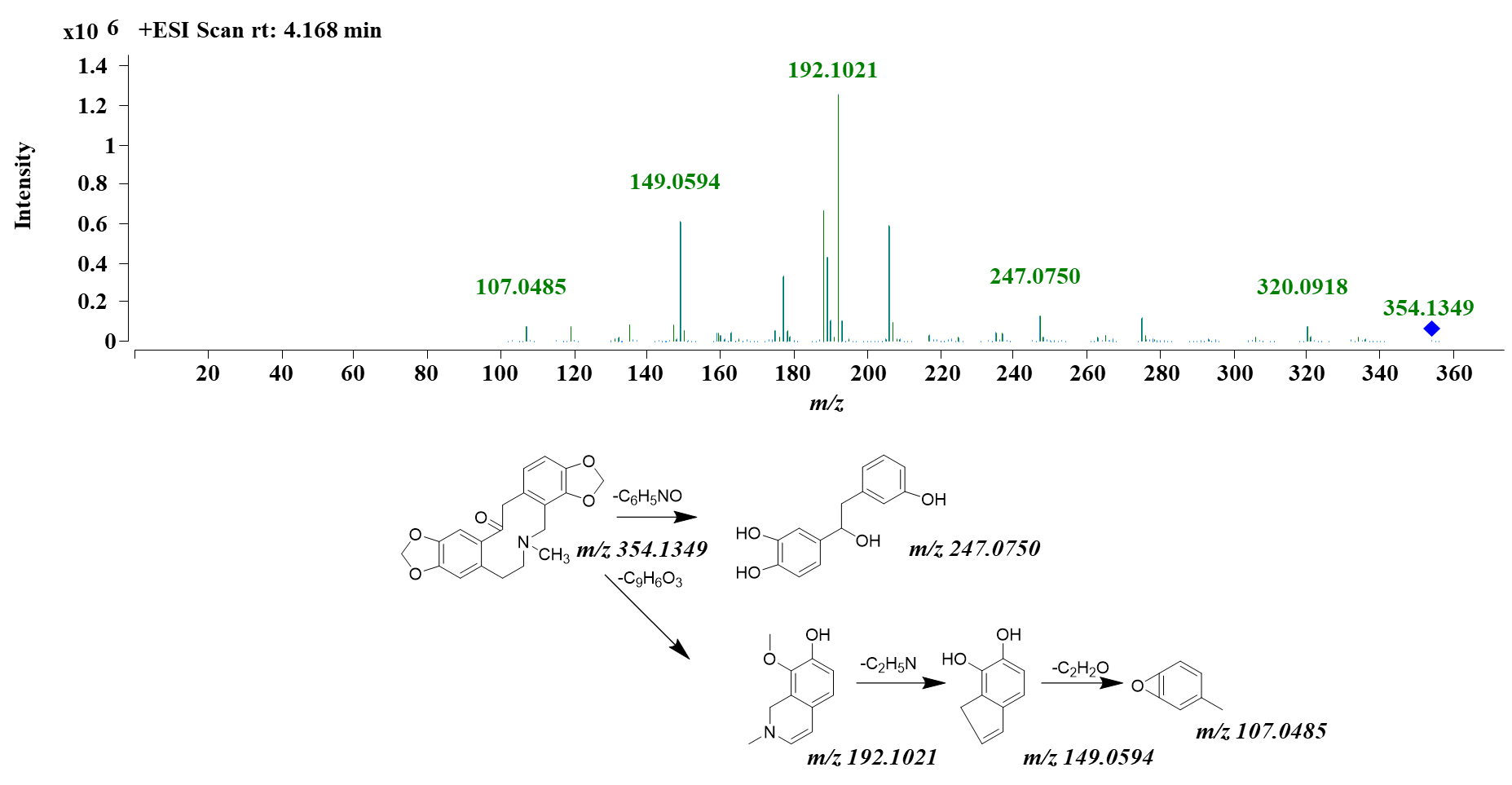
 FIGURE S12 Tentative fragmentation pathway and mass spectrogram of compound 11 (Protopine).


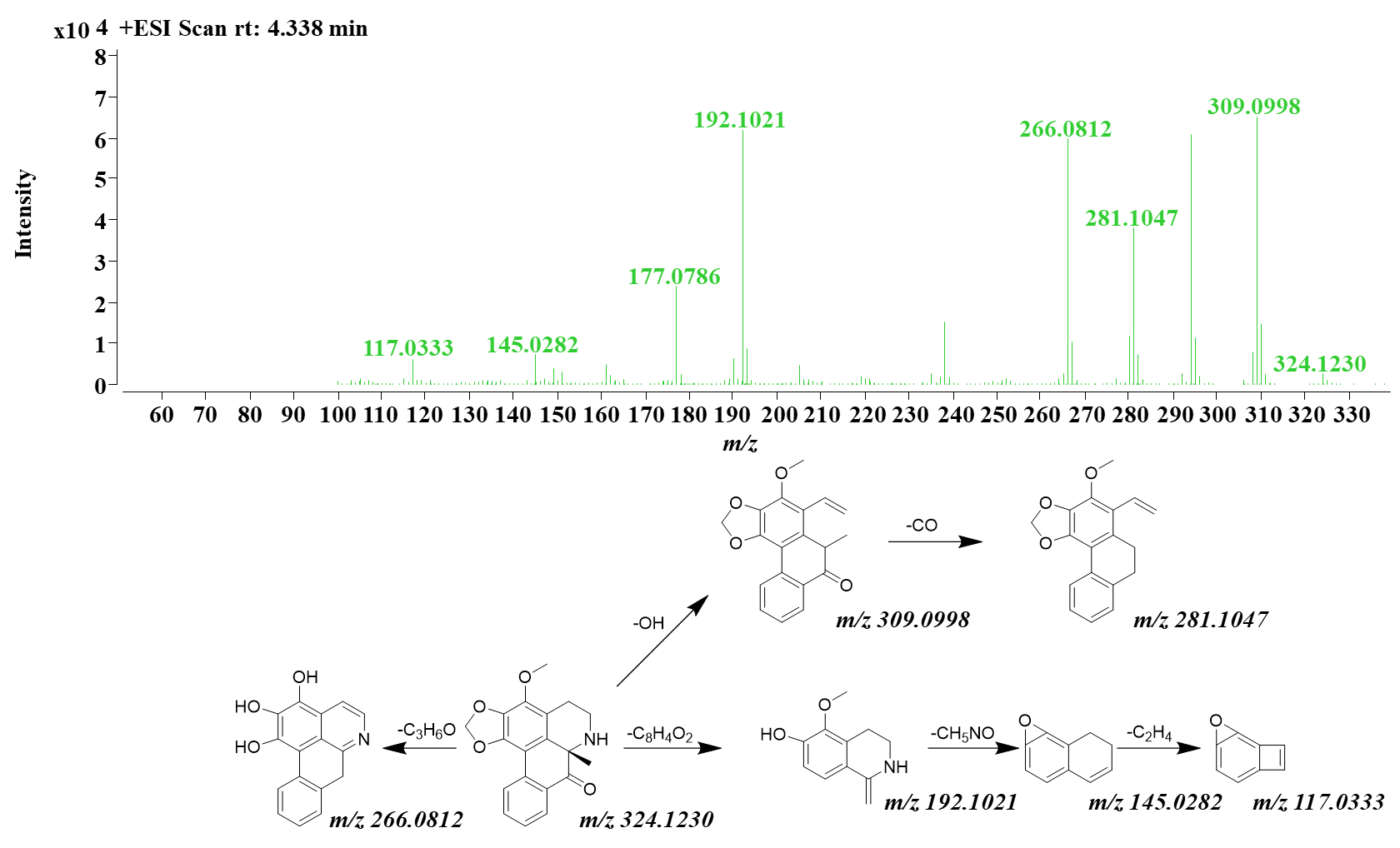
 FIGURE S13 Tentative fragmentation pathway and mass spectrogram of compound 12 (Analogue of liriodenine).


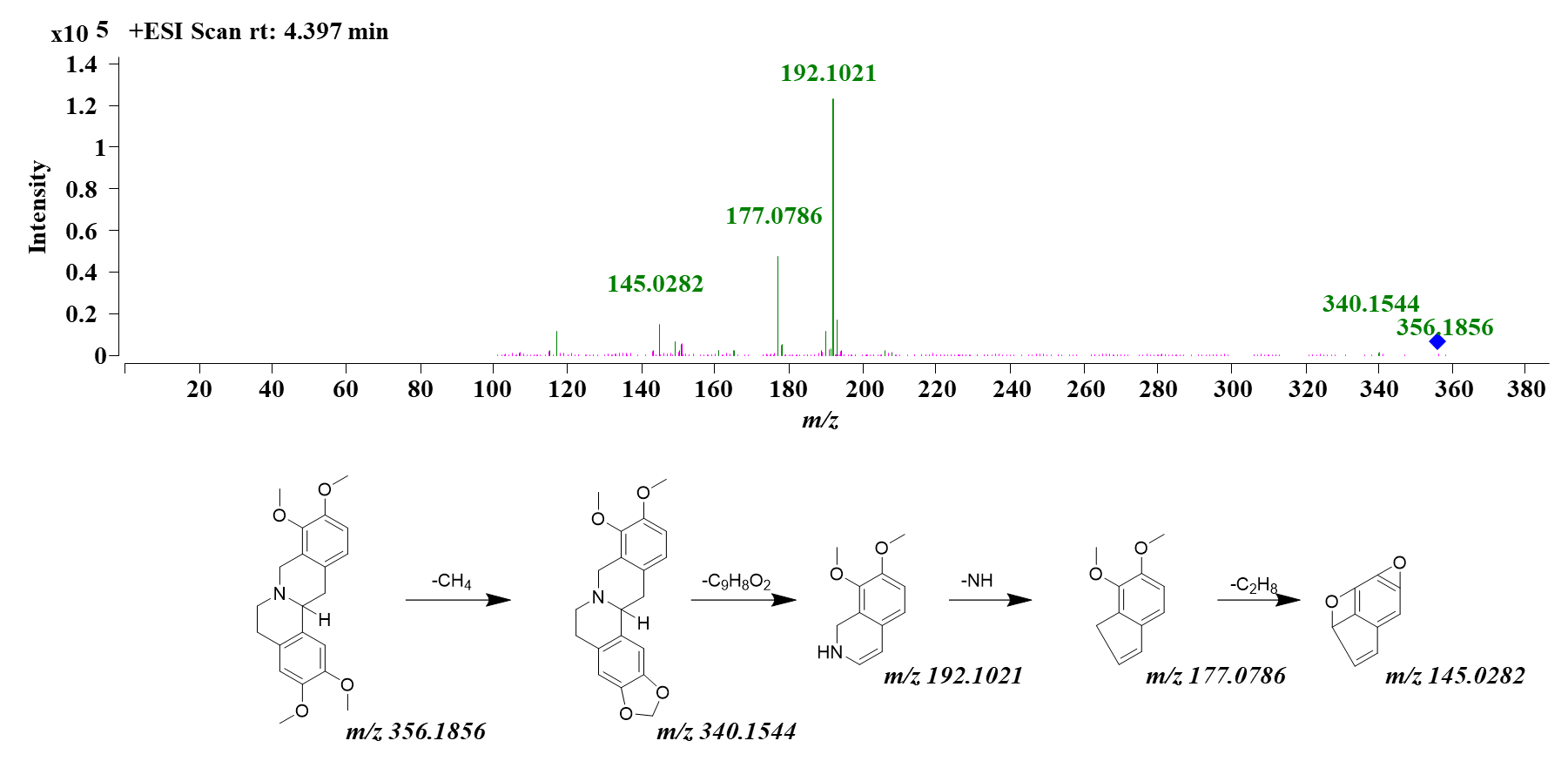
 FIGURE S14 Tentative fragmentation pathway and mass spectrogram of compound 13 (Tertrahydropaimatine).


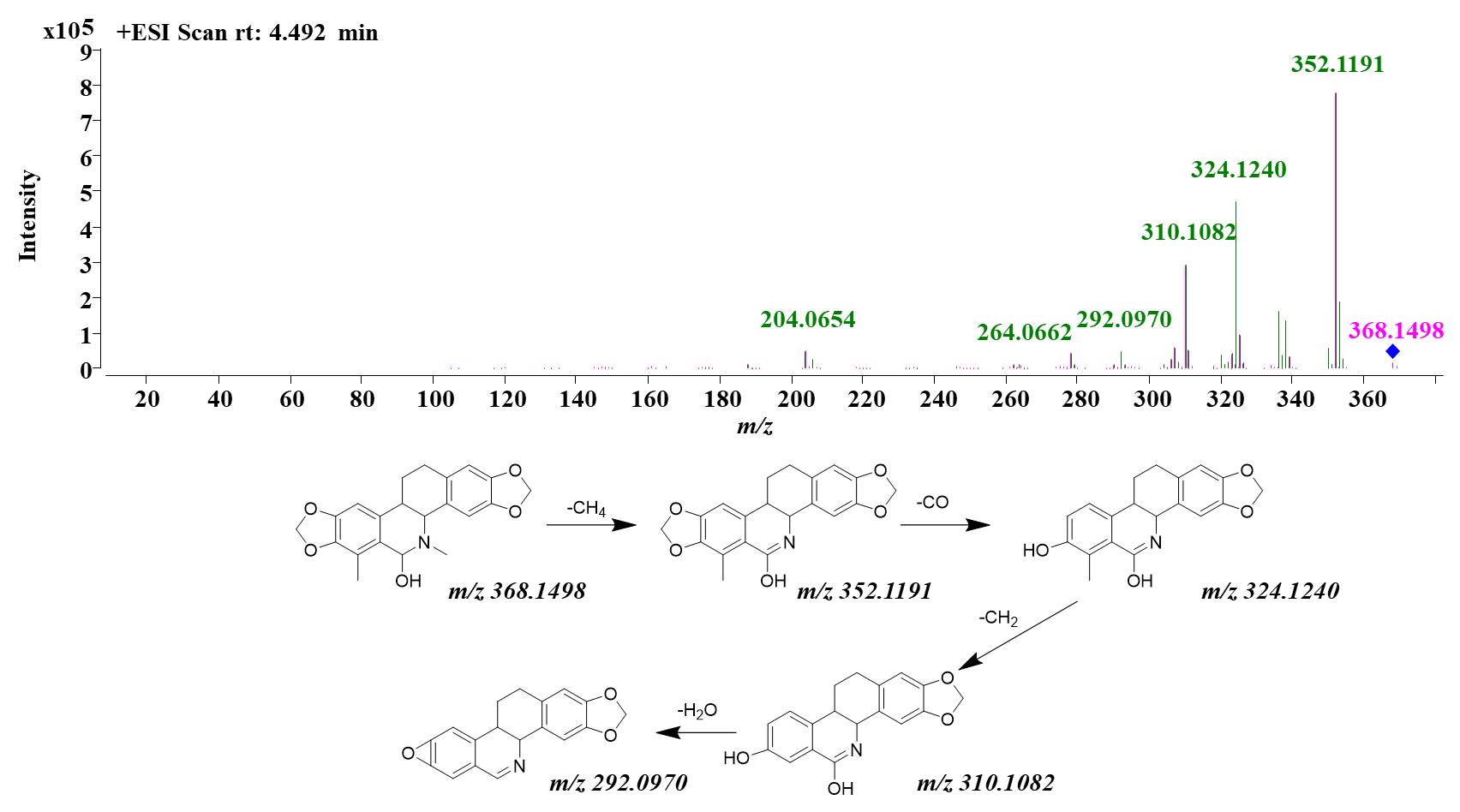
 FIGURE S15 Tentative fragmentation pathway and mass spectrogram of compound 14 (Analogue of oxyavicine).


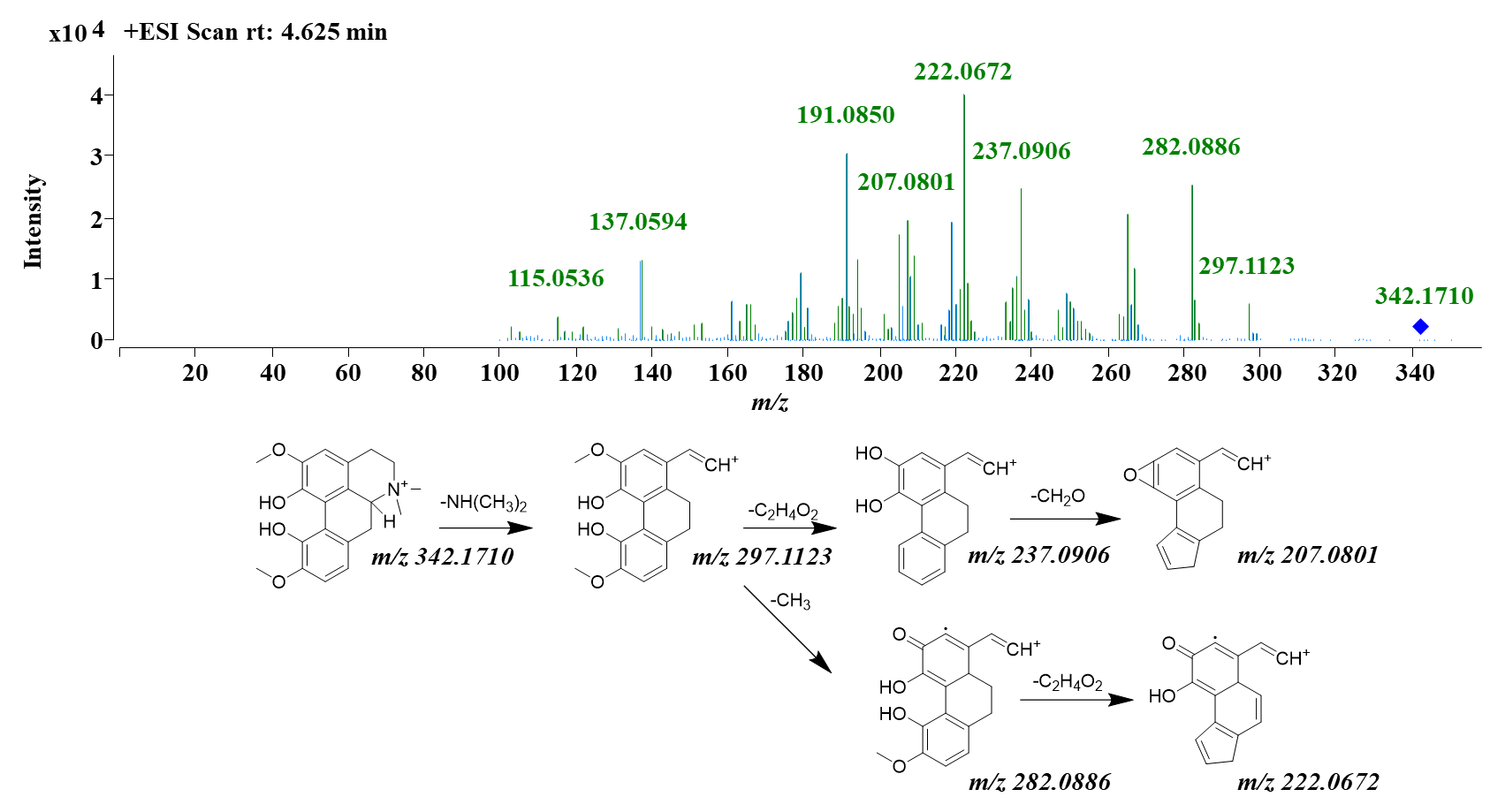
 FIGURE S16 Tentative fragmentation pathway and mass spectrogram of compound 15 (Magnoflorine B).


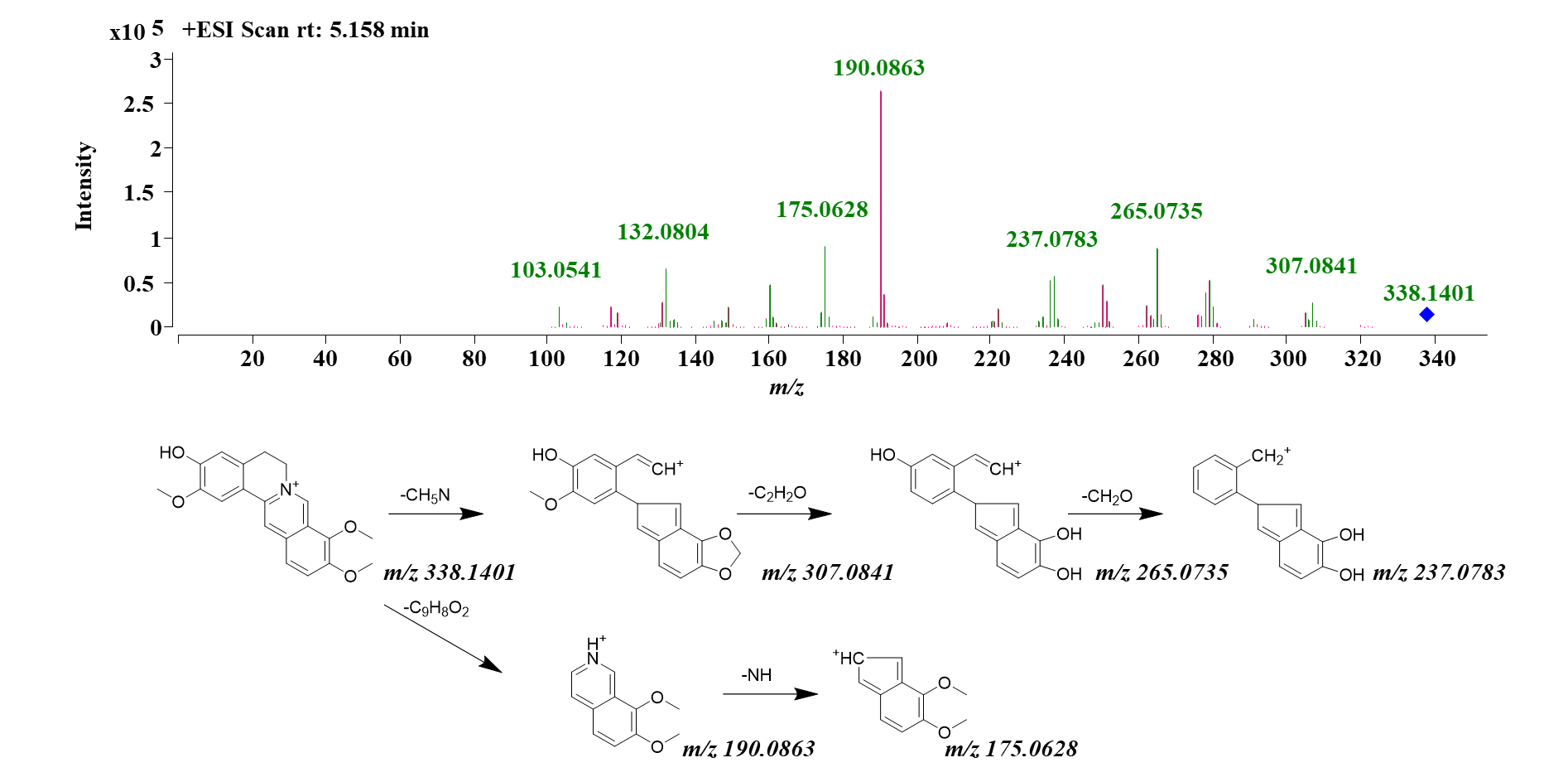
 FIGURE S17 Tentative fragmentation pathway and mass spectrogram of compound 16 (Palmatrubin).


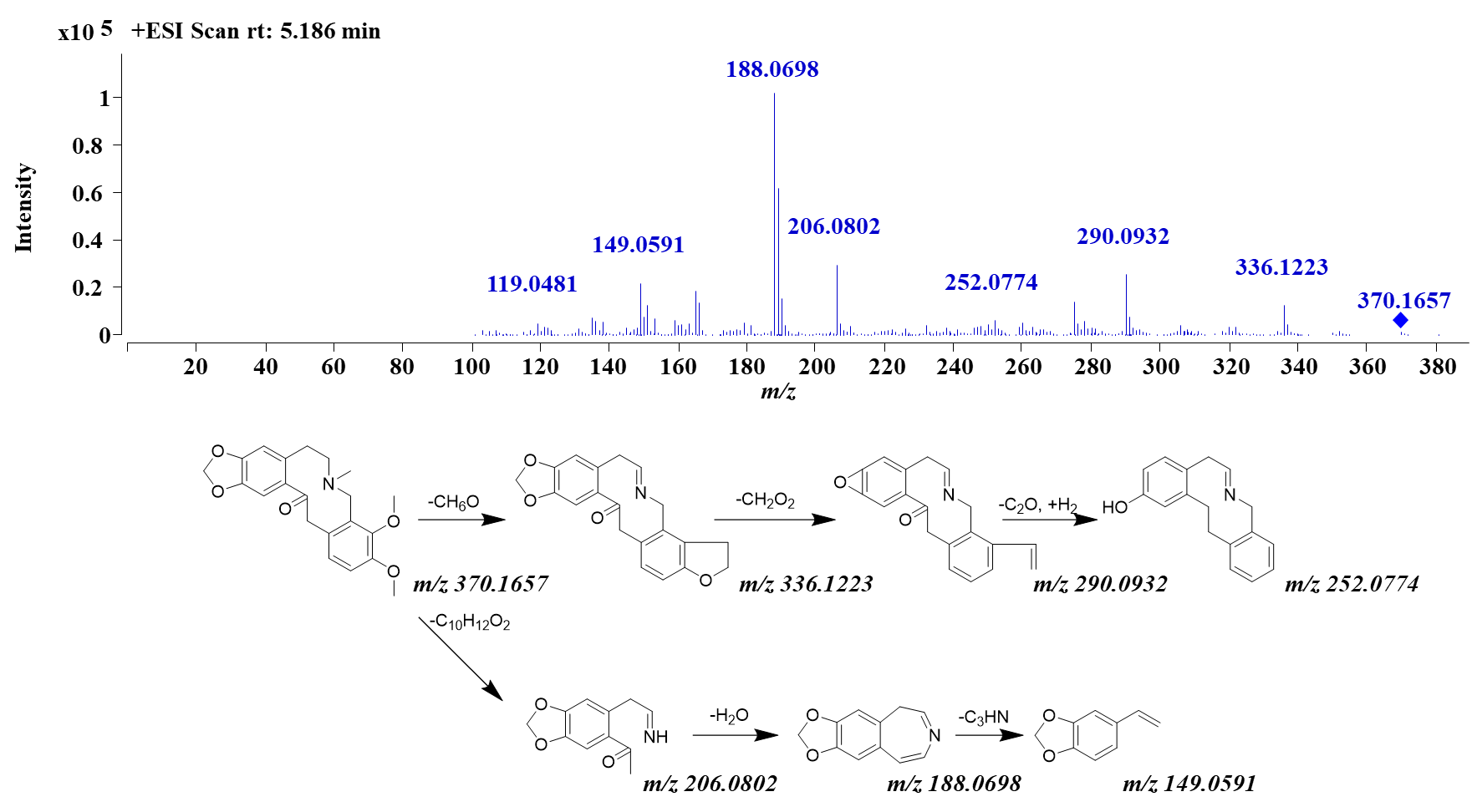
 FIGURE S18 Tentative fragmentation pathway and mass spectrogram of compound 17 (Allocryptopine).


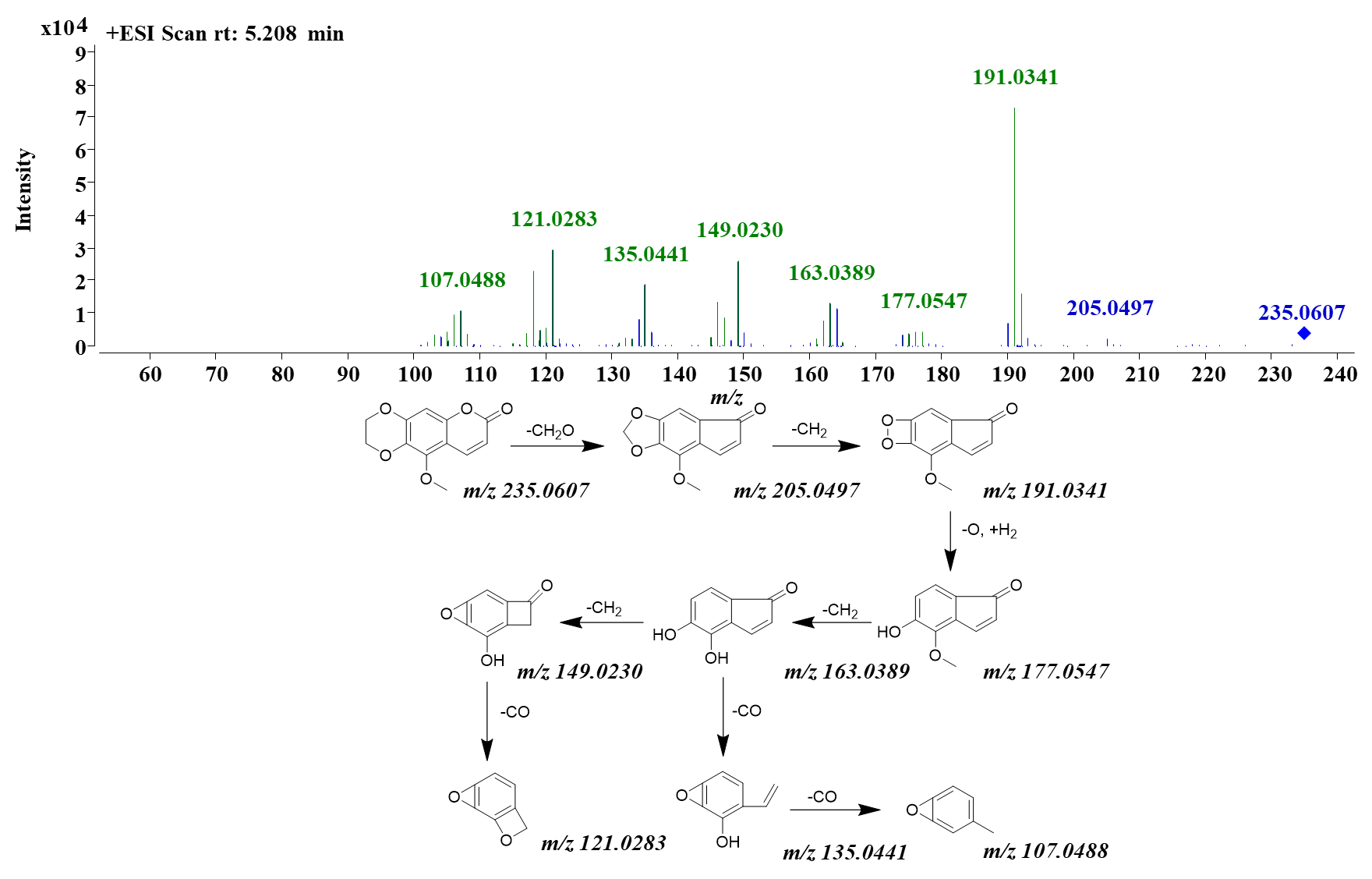
 FIGURE S19 Tentative fragmentation pathway and mass spectrogram of compound 18 (10-methoxy-2,3-dihydro-7H - [1,4] dioxino[2,3-g] chromen-7-one).


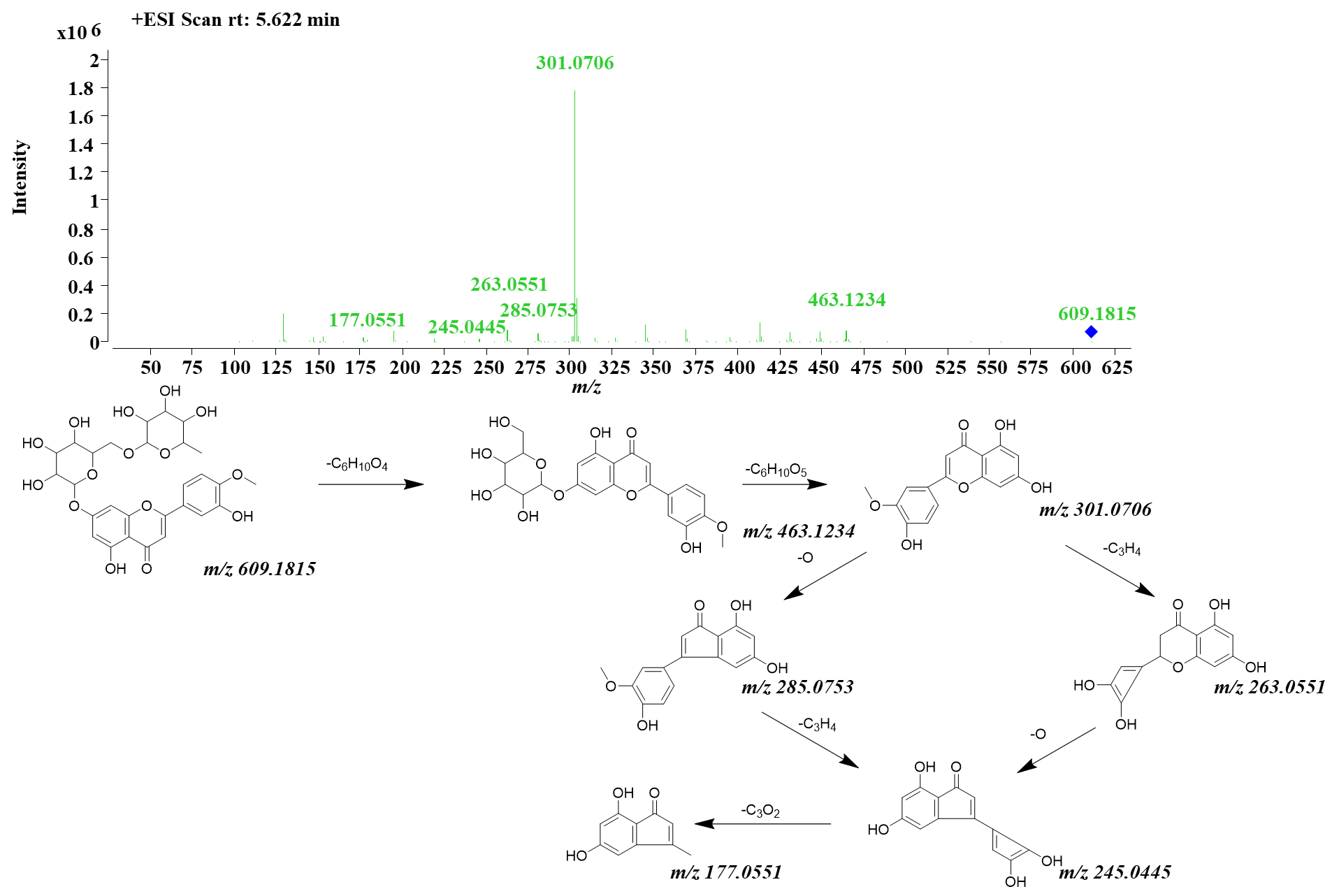
 FIGURE S20 Tentative fragmentation pathway and mass spectrogram of compound 20 (Diosmin).


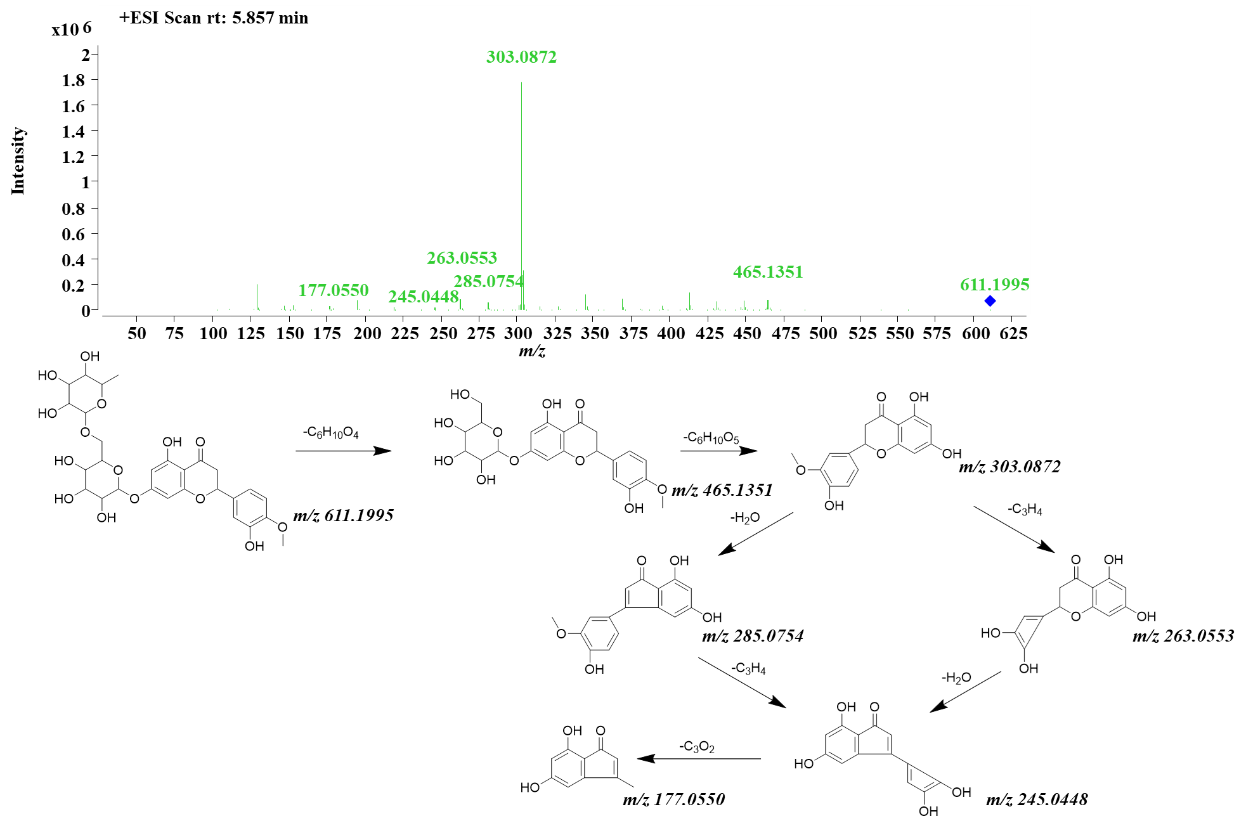
 FIGURE S21 Tentative fragmentation pathway and mass spectrogram of compound 21 (Hesperidin).


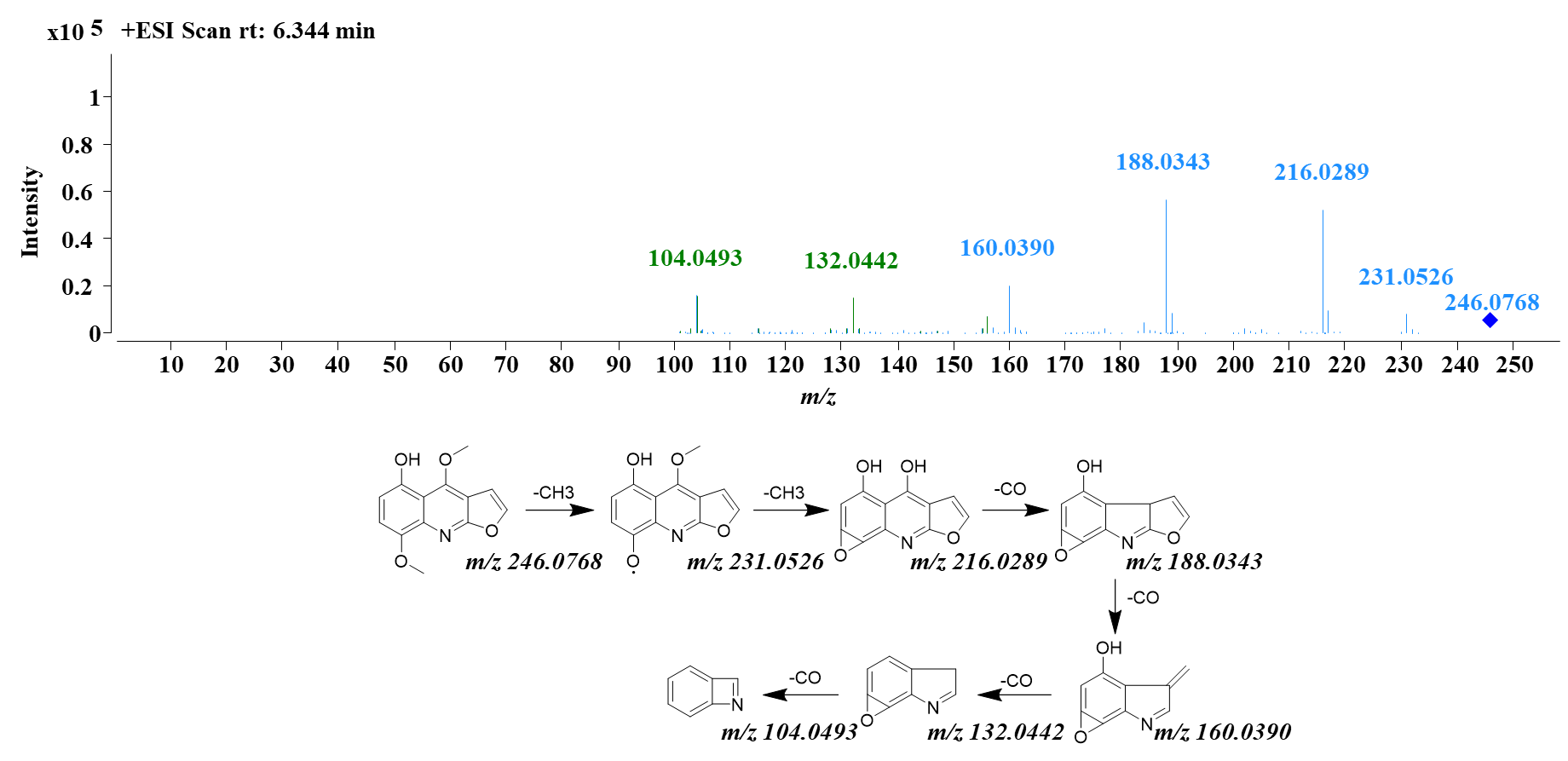
 FIGURE S22 Tentative fragmentation pathway and mass spectrogram of compound 22 (Haplopine).


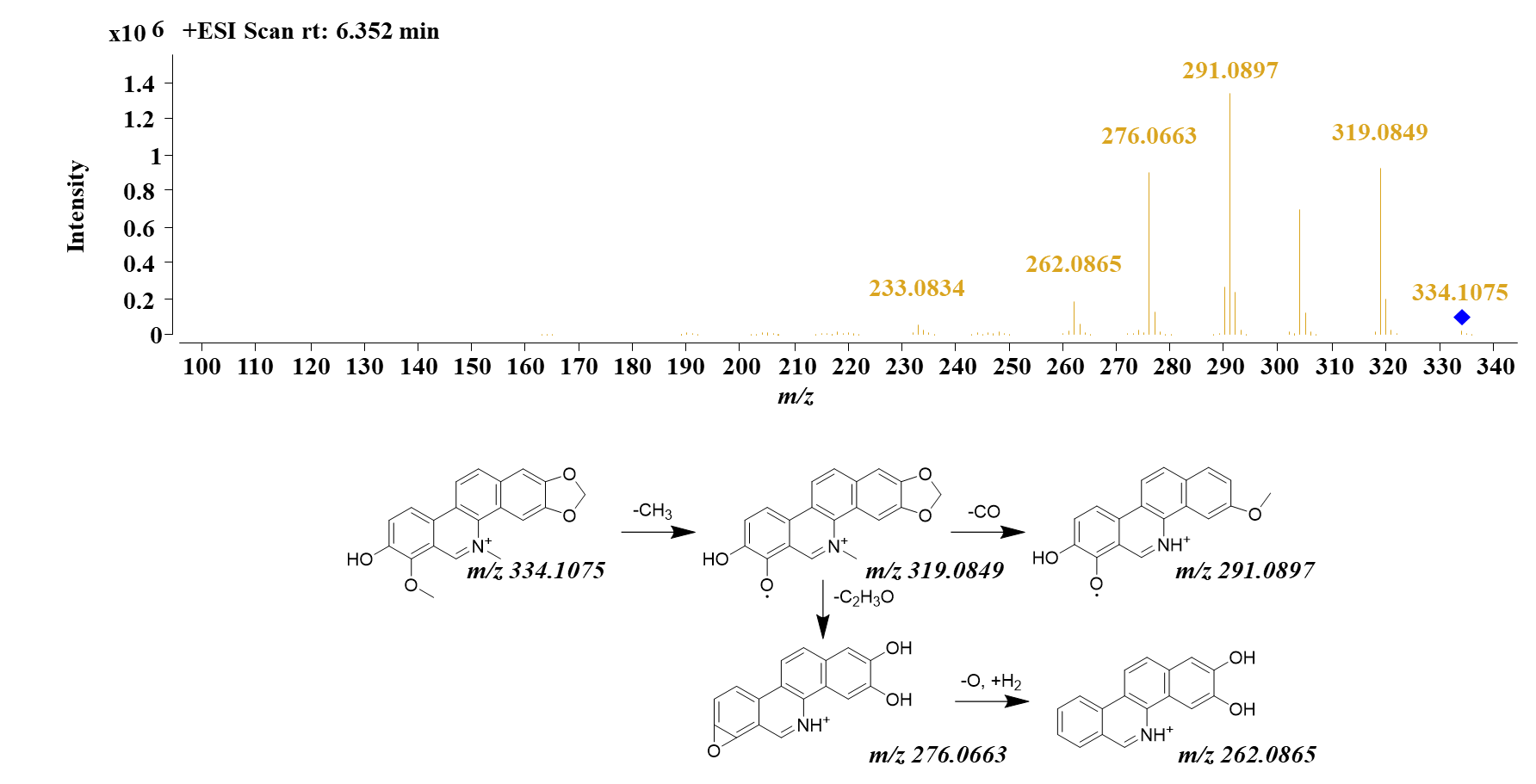
 FIGURE S23 Tentative fragmentation pathway and mass spectrogram of compound 23 (Isofagaridine).


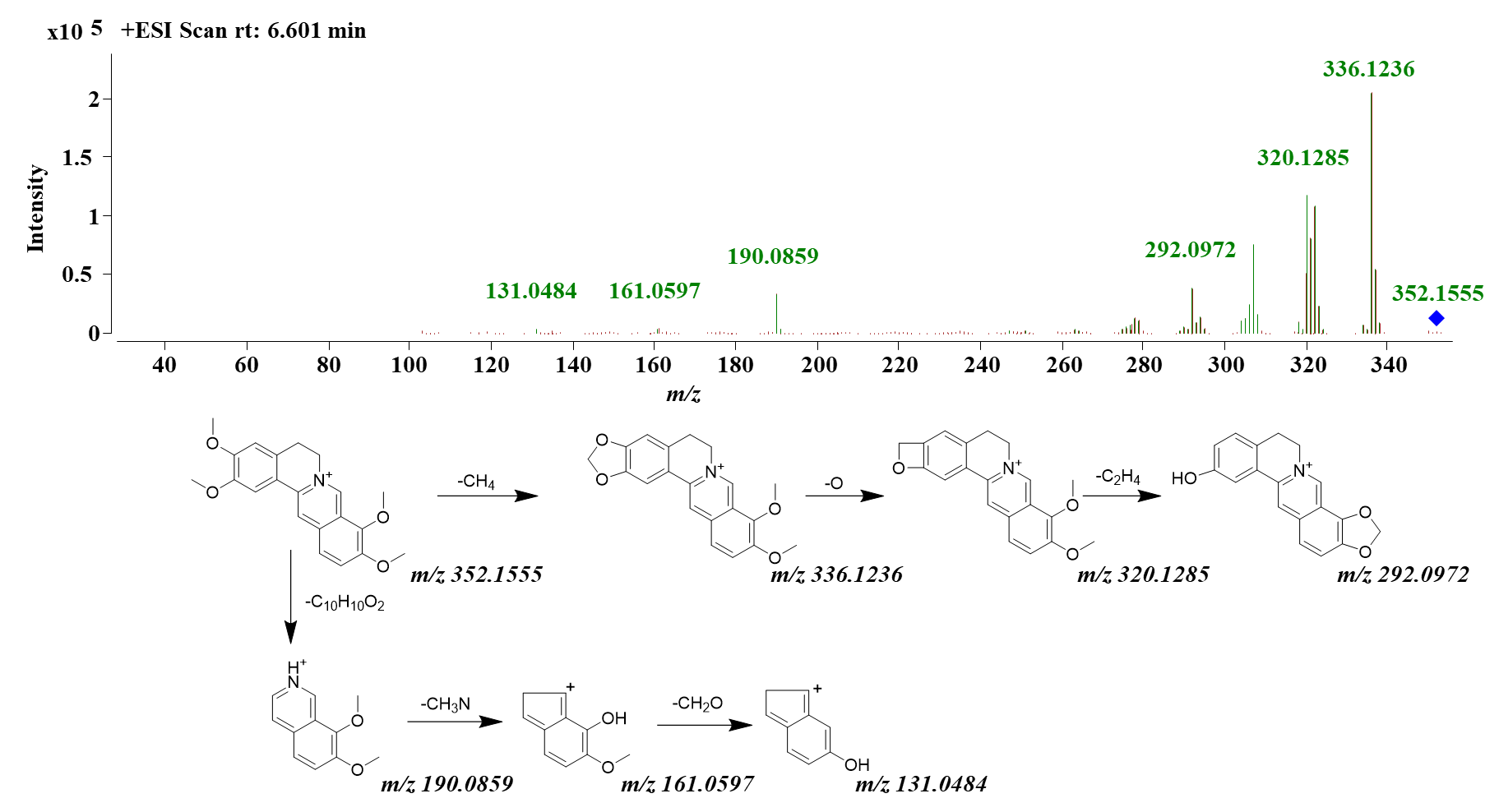
 FIGURE S24 Tentative fragmentation pathway and mass spectrogram of compound 24 (Palmatine).


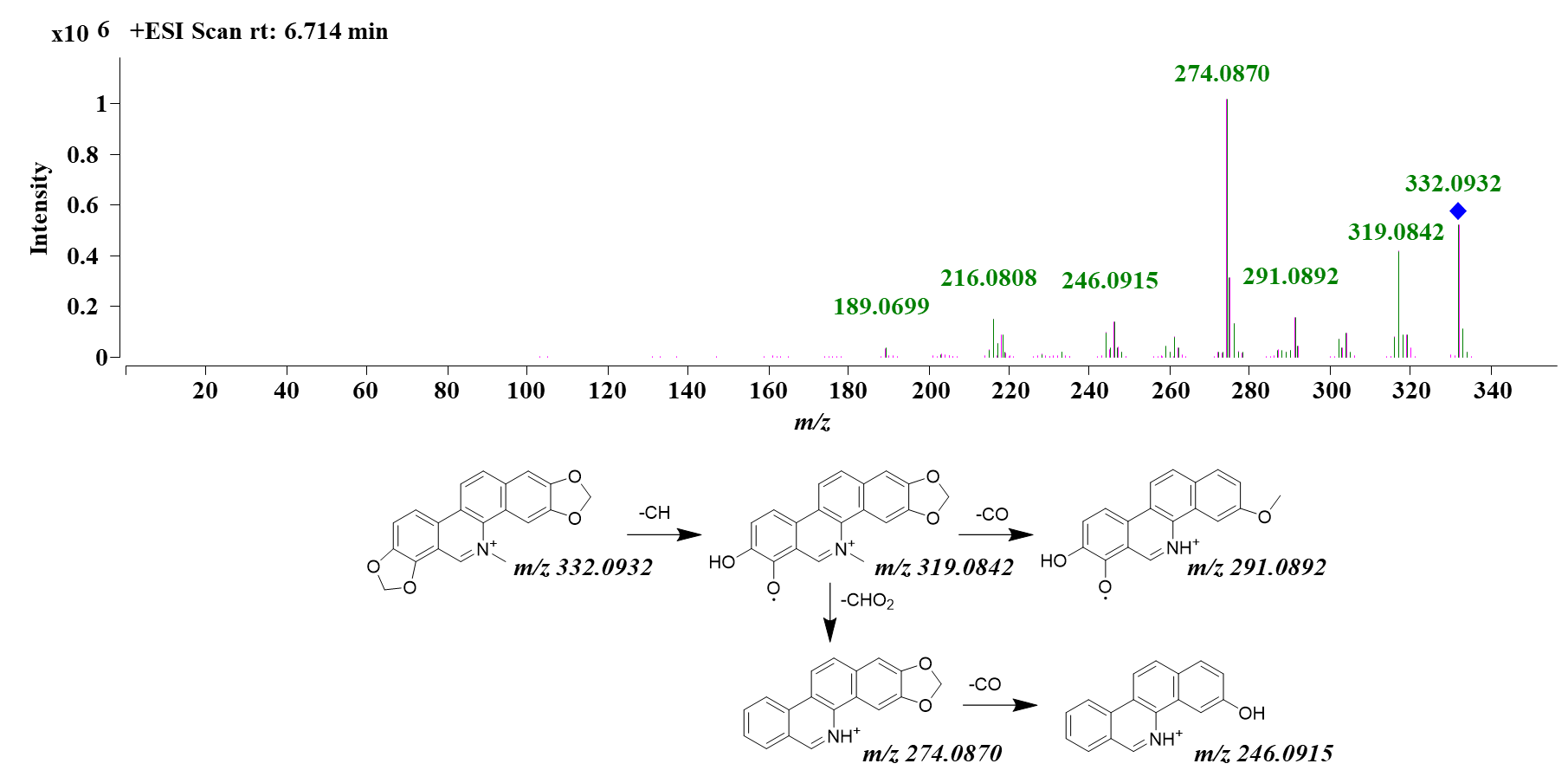
 FIGURE S25 Tentative fragmentation pathway and mass spectrogram of compound 25 (Sanguinarine A).


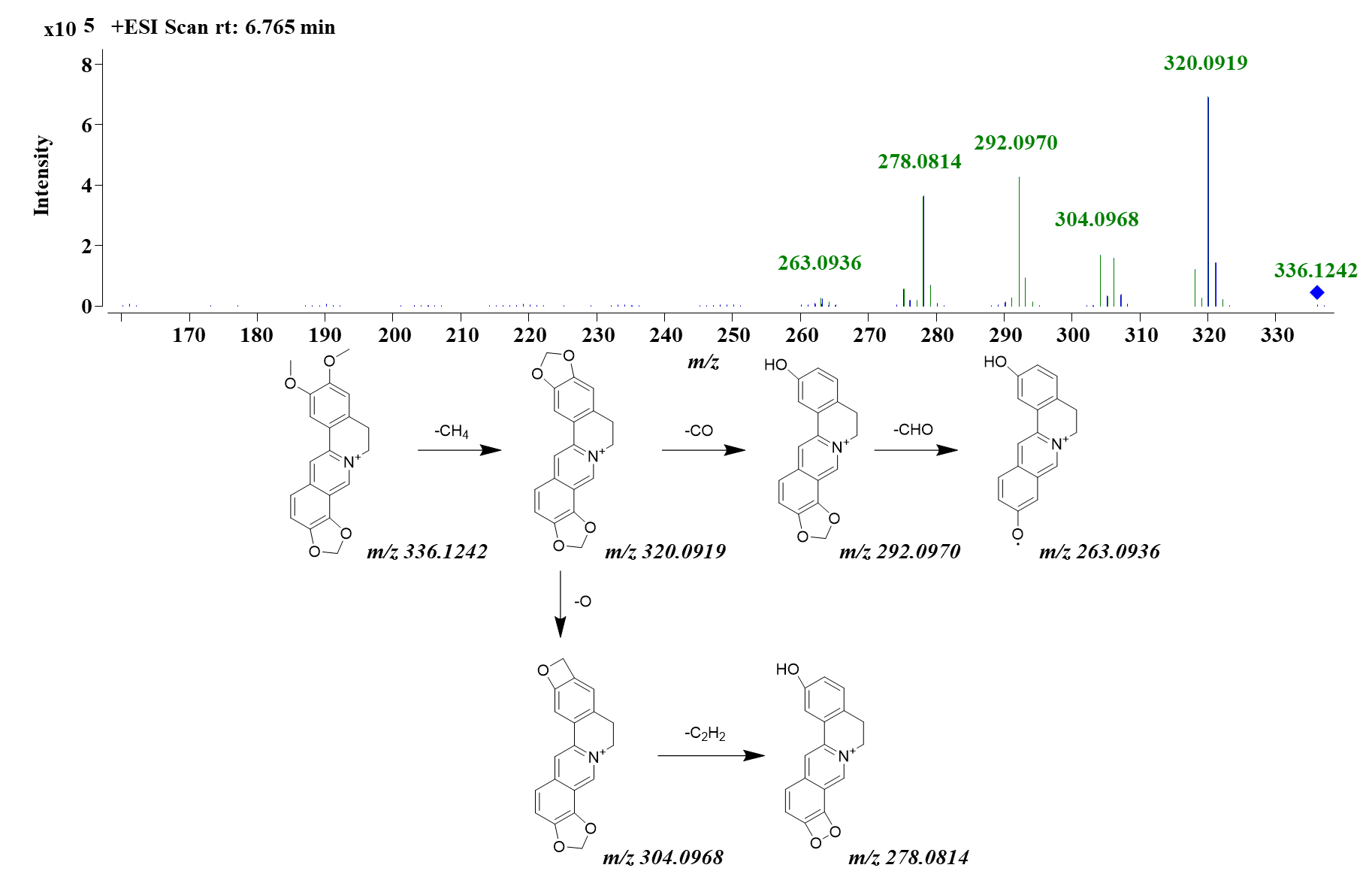
 FIGURE S26 Tentative fragmentation pathway and mass spectrogram of compound 26 (Epiberberine).


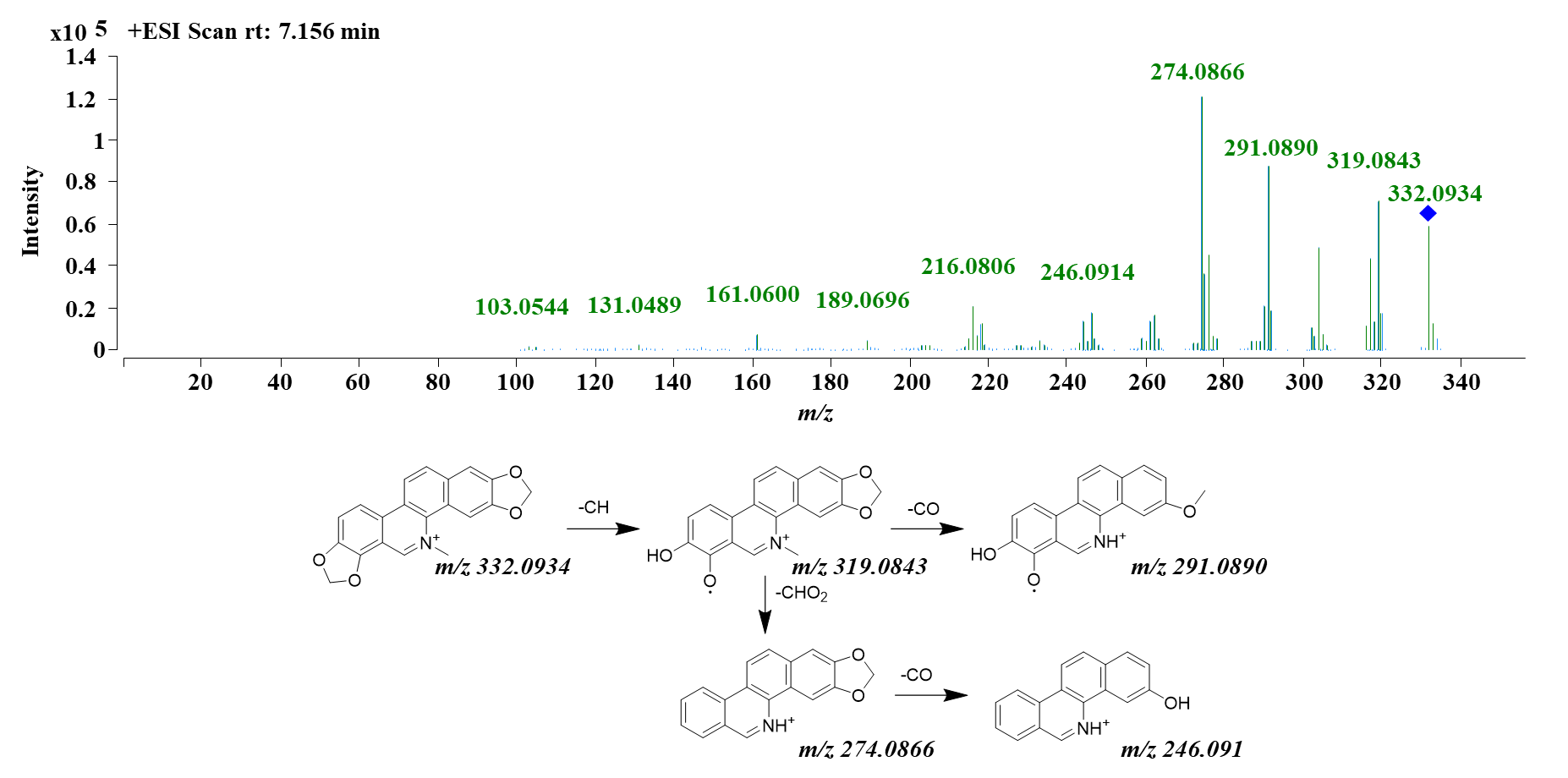
 FIGURE S27 Tentative fragmentation pathway and mass spectrogram of compound 27 (Sanguinarine B).


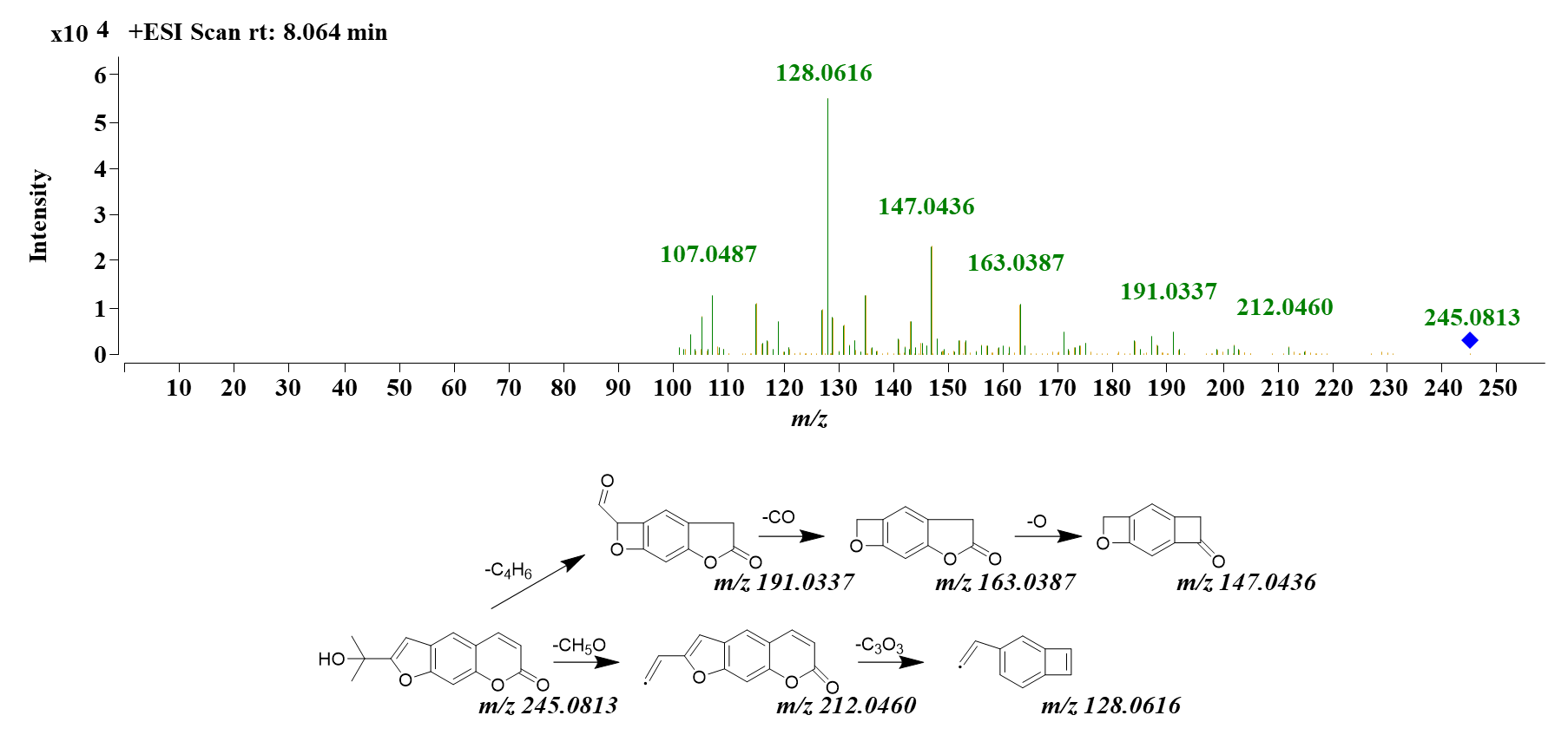
 FIGURE S28 Tentative fragmentation pathway and mass spectrogram of compound 28 (Marmesin).


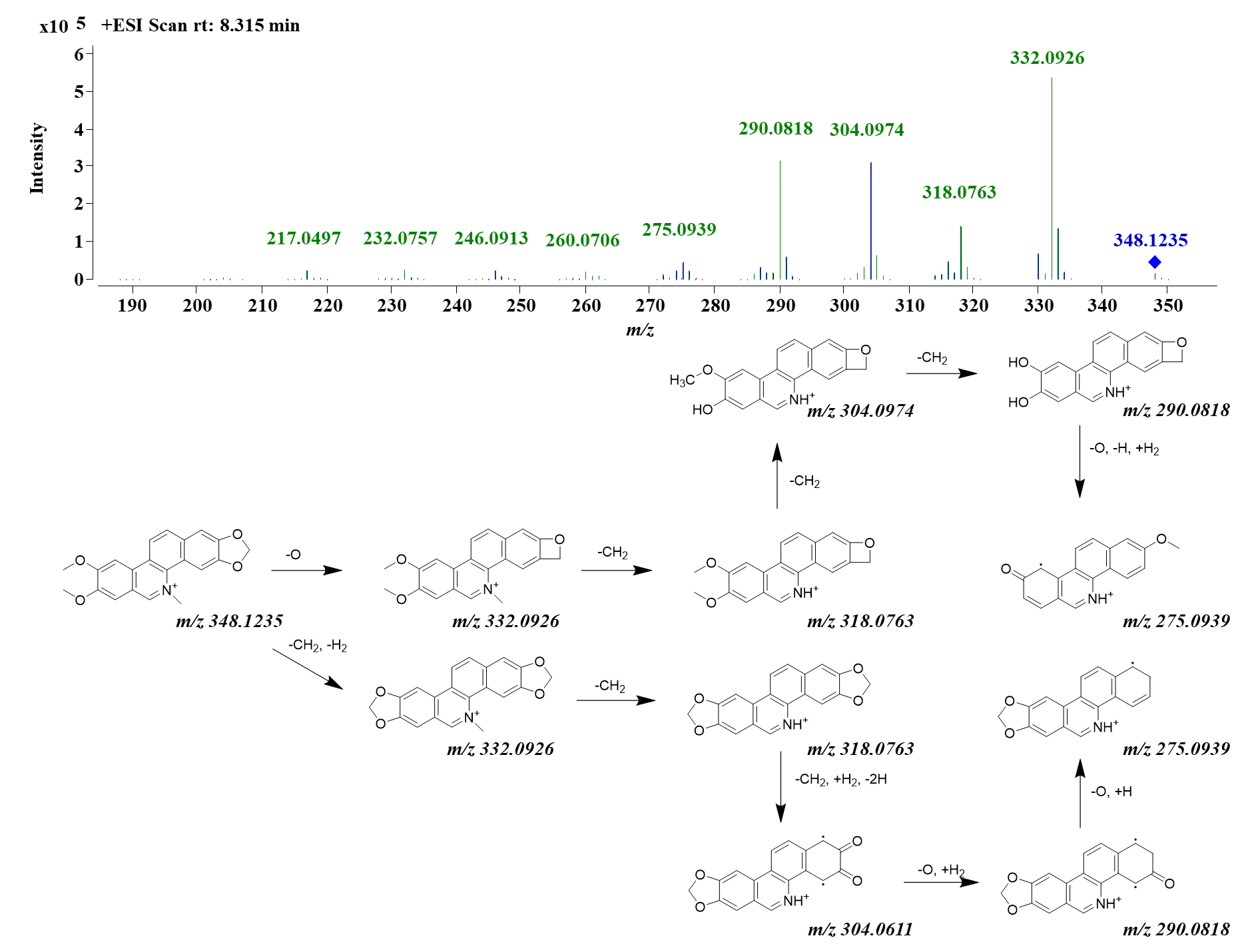
 FIGURE S29 Tentative fragmentation pathway and mass spectrogram of compound 29 (Nitidine A).


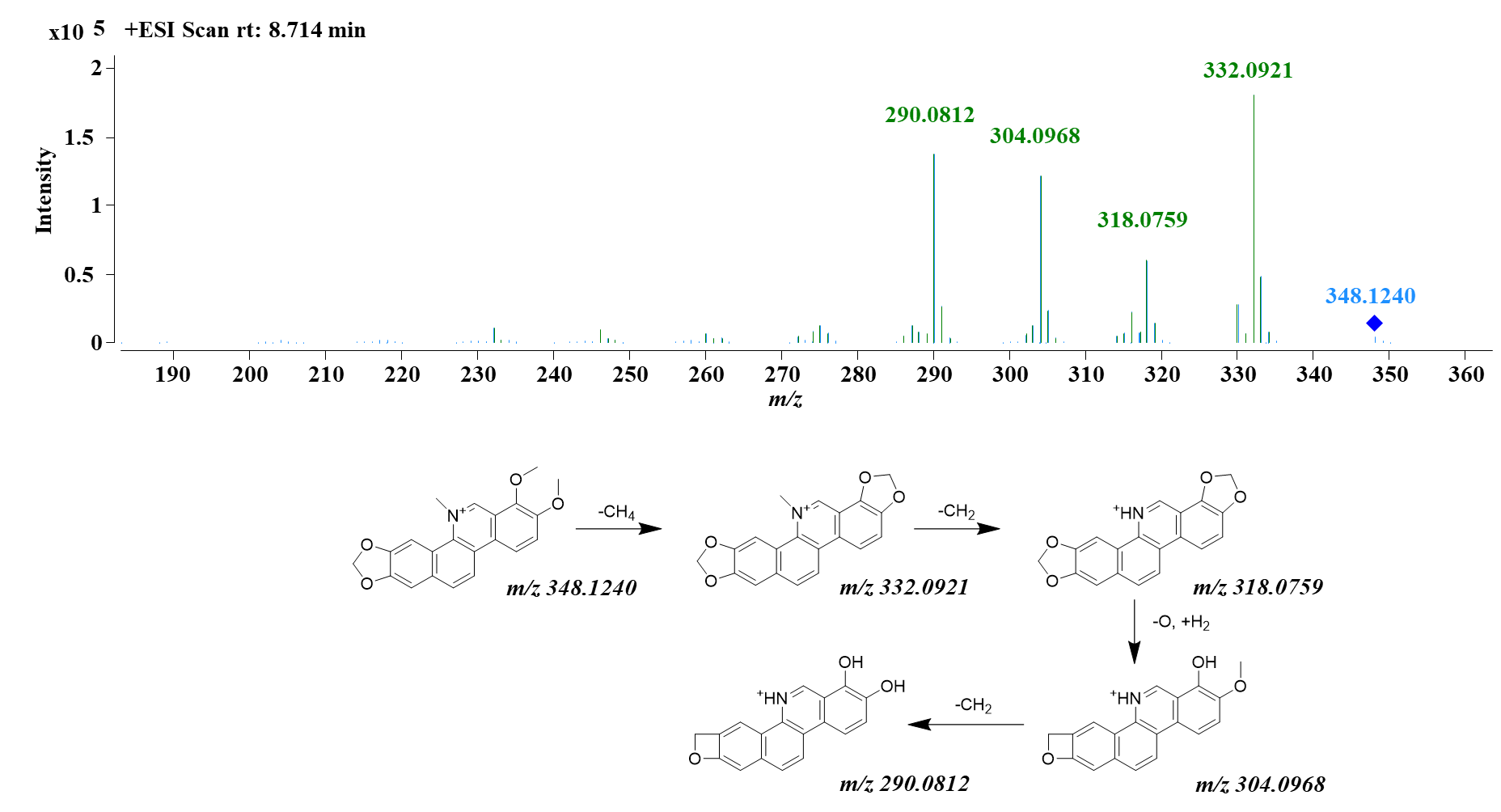
 FIGURE S30 Tentative fragmentation pathway and mass spectrogram of compound 30 (Chelerythrine).


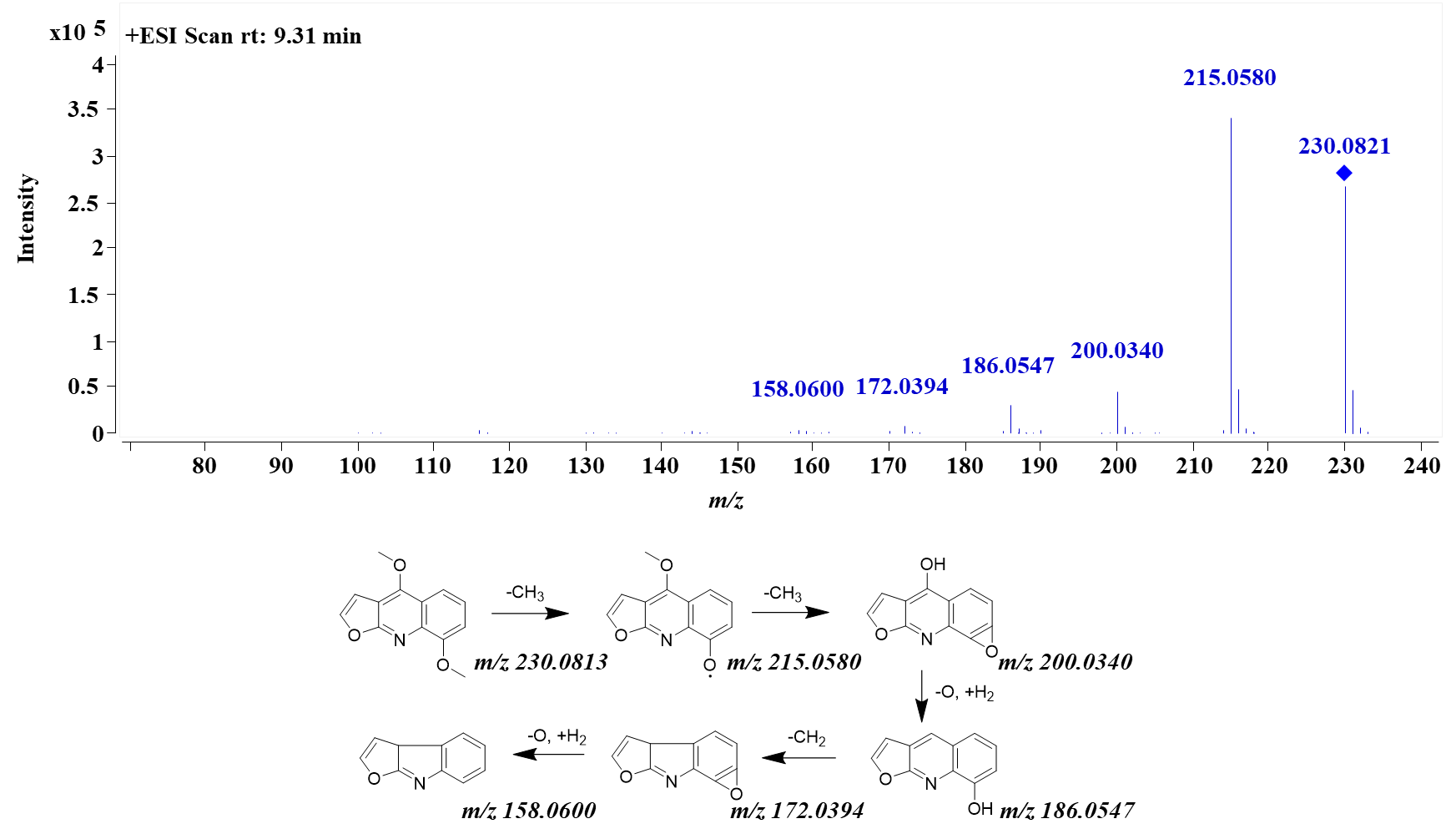
 FIGURE S31 Tentative fragmentation pathway and mass spectrogram of compound 31 (γ-Fagarine).


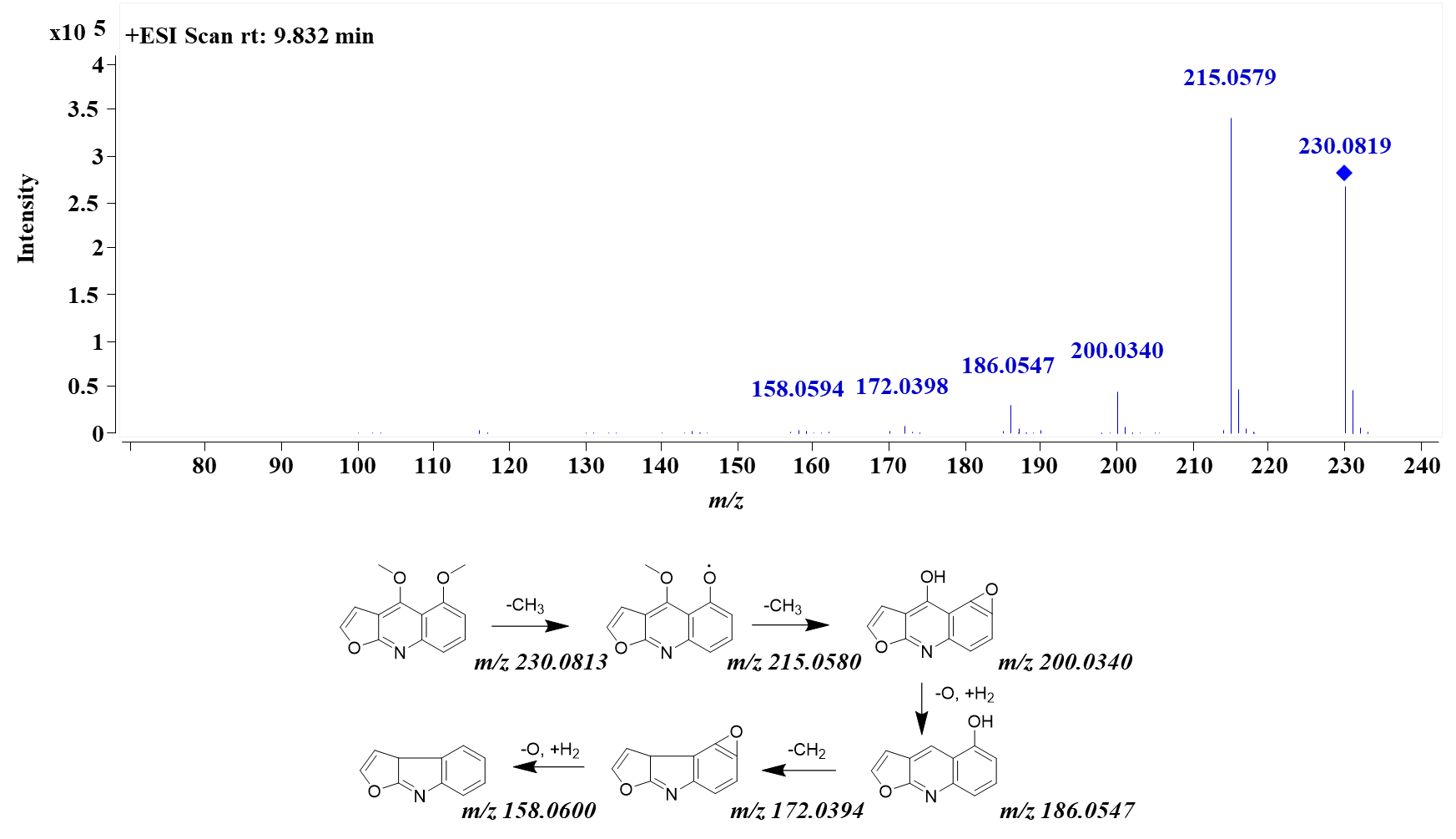
 FIGURE S32 Tentative fragmentation pathway and mass spectrogram of compound 33 (5-methoxydictamnine).


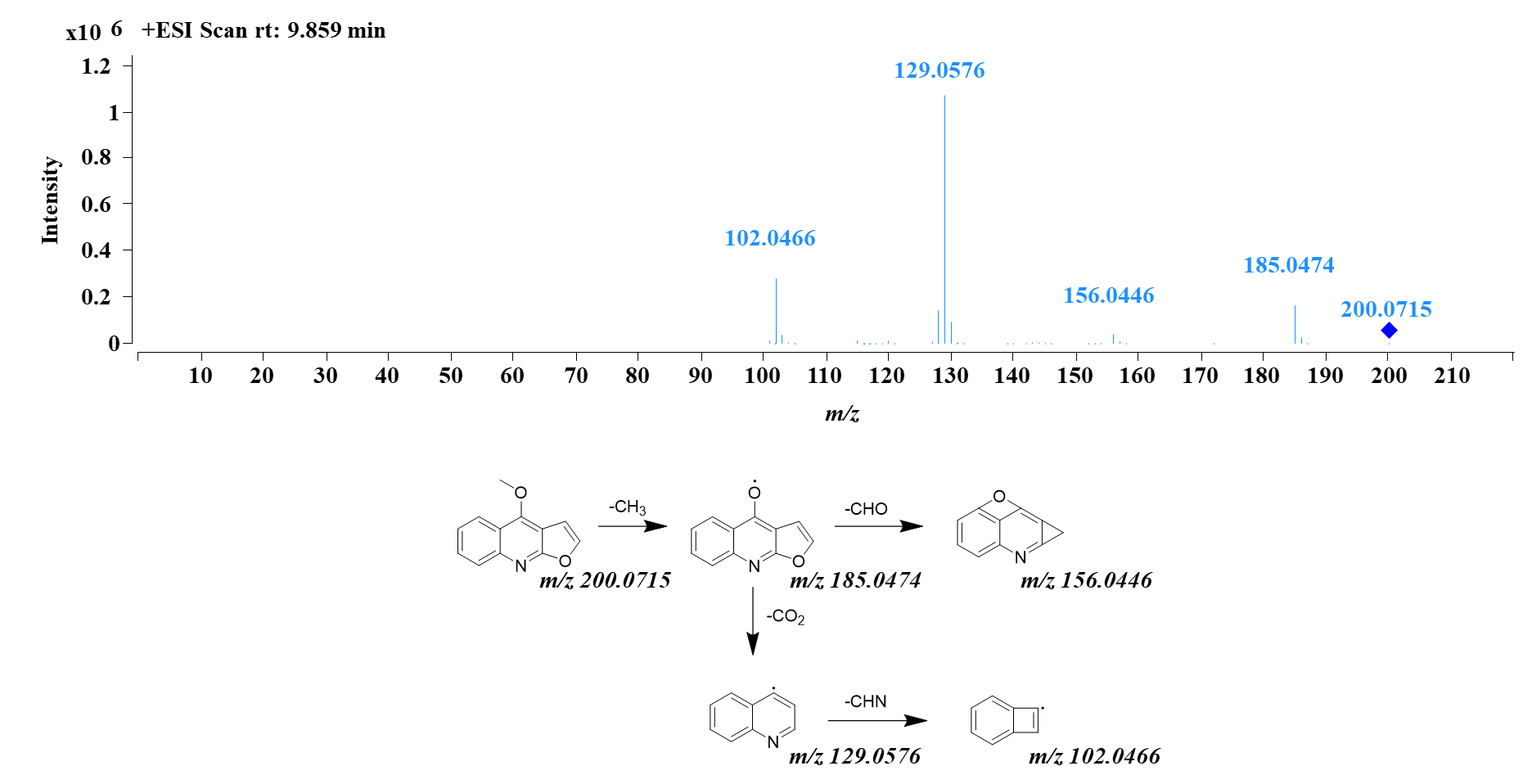
 FIGURE S33 Tentative fragmentation pathway and mass spectrogram of compound 34 (Dictamnine).


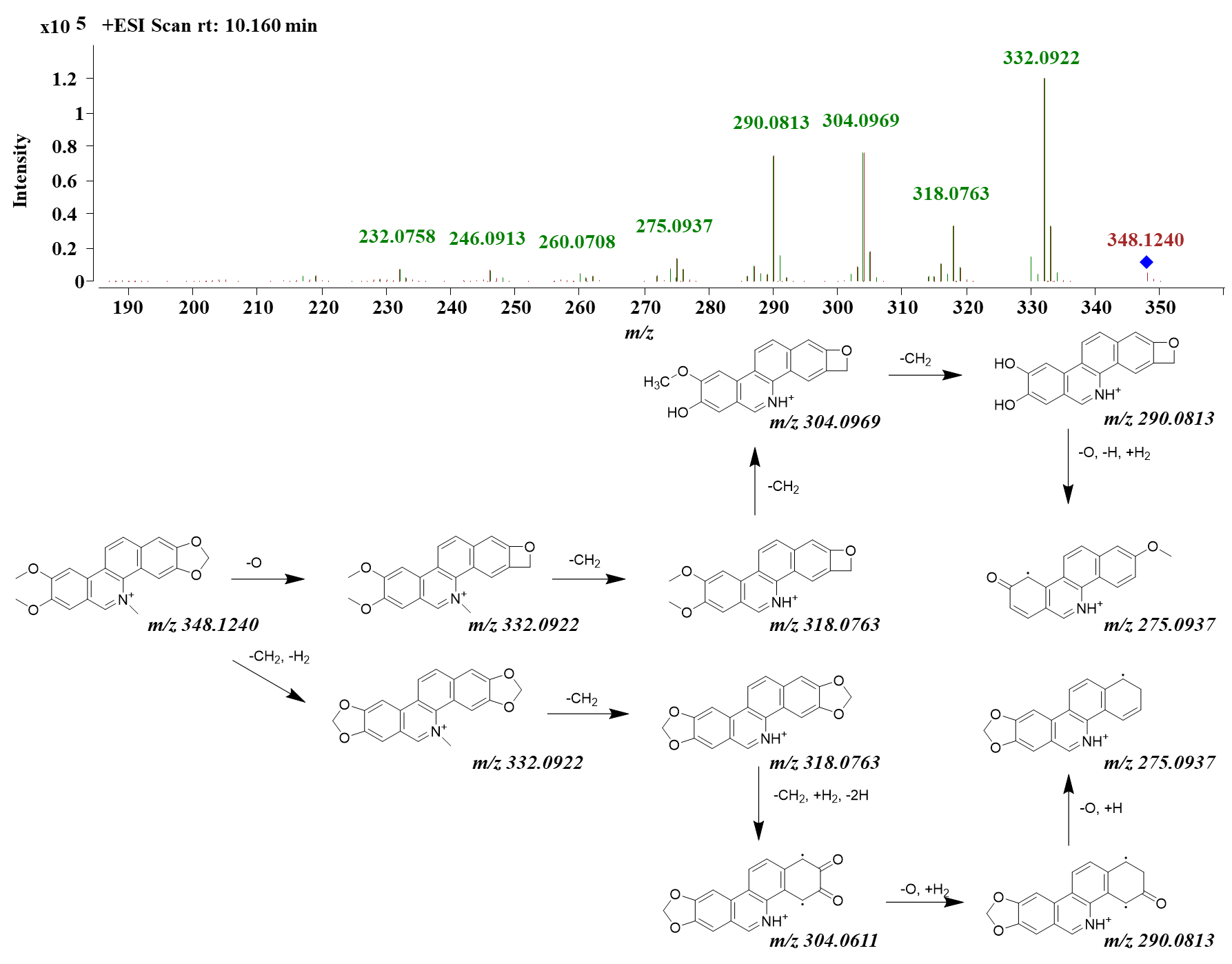
 FIGURE S34 Tentative fragmentation pathway and mass spectrogram of compound 35 (Nitidine B).


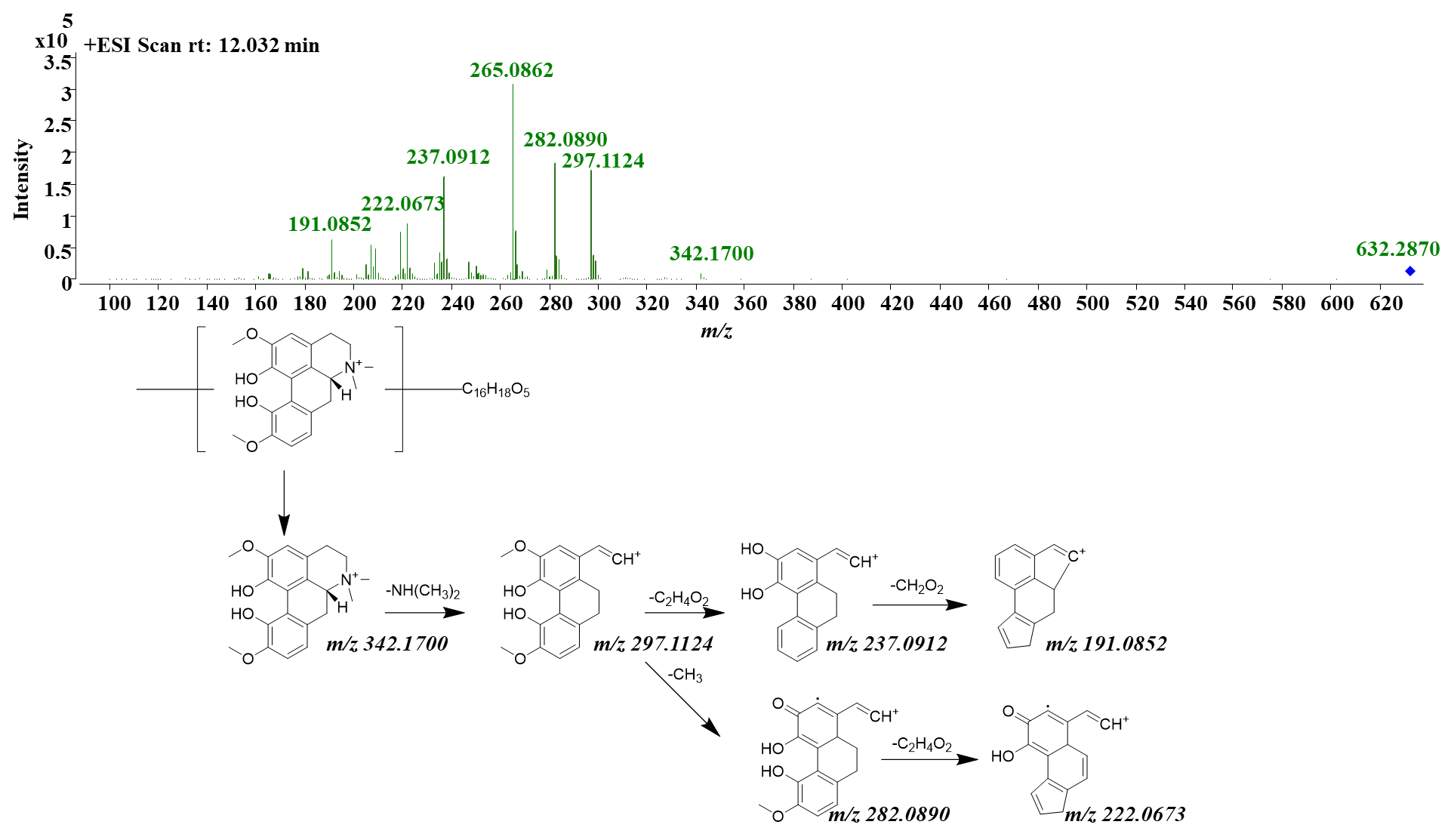
 FIGURE S35 Tentative fragmentation pathway and mass spectrogram of compound 37 (Analogue of Magnoflorine).


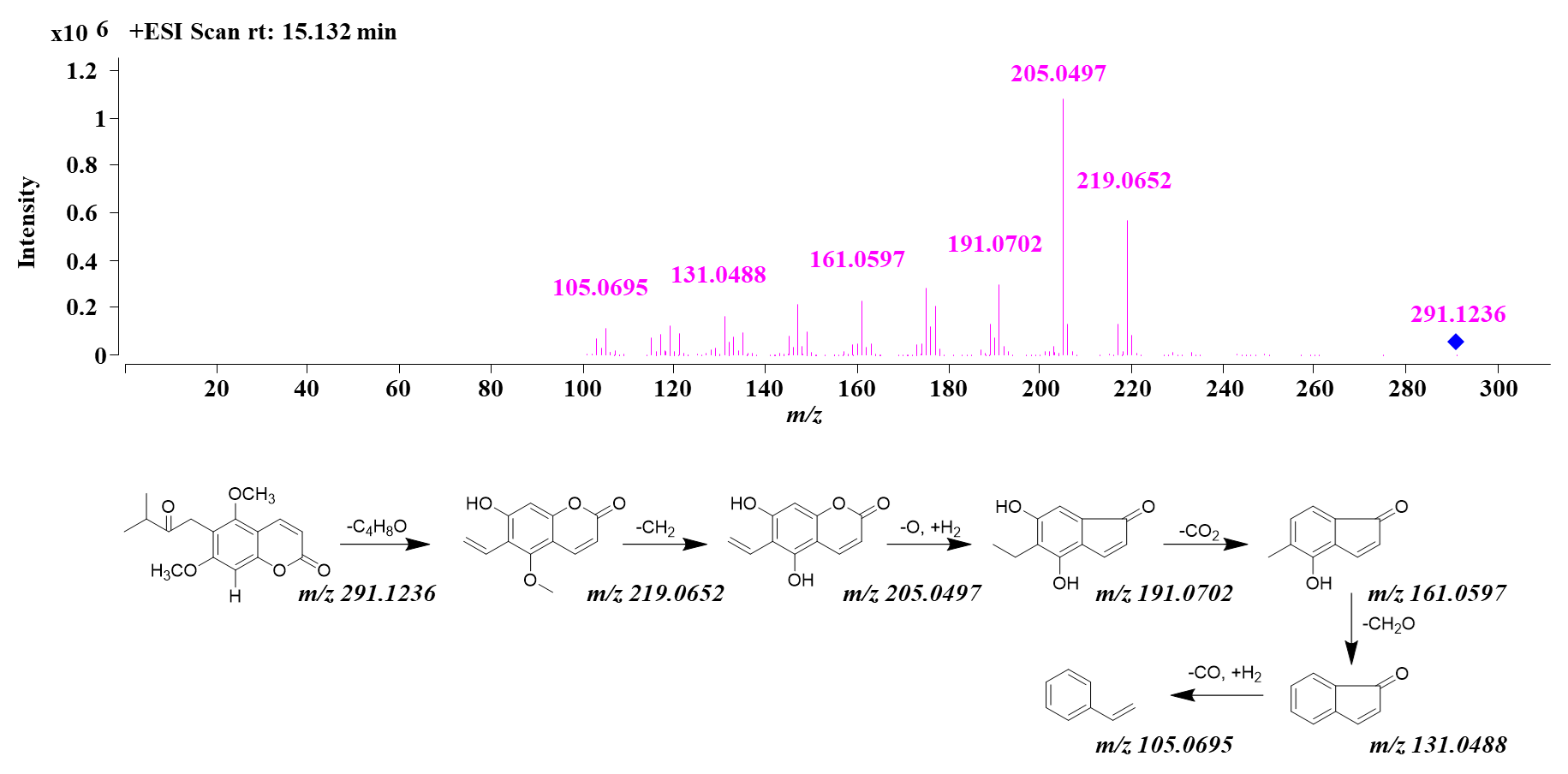
FIGURE S36 Tentative fragmentation pathway and mass spectrogram of compound 38 (Toddanone).


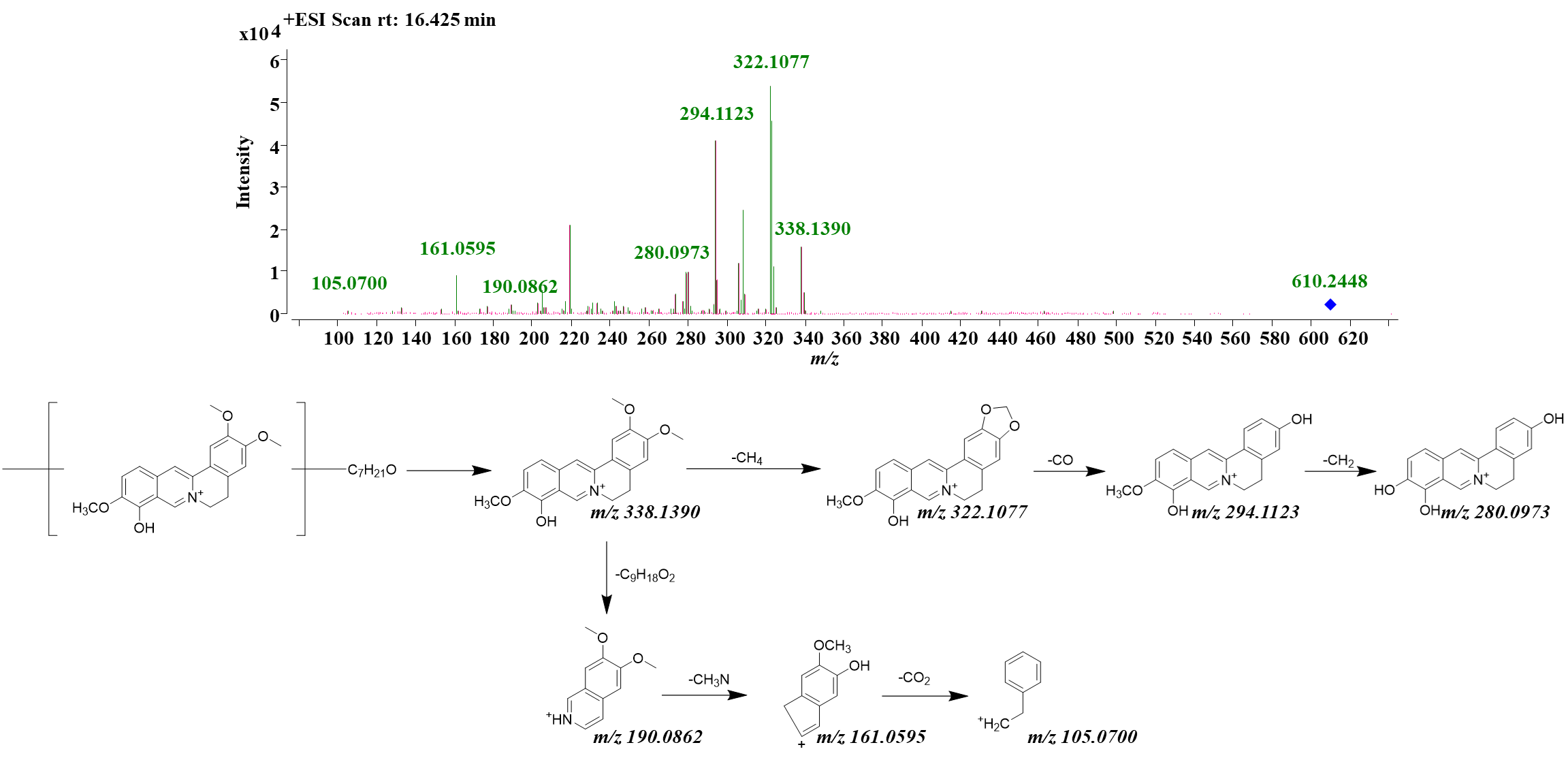


FIGURE S37 Tentative fragmentation pathway and mass spectrogram of compound 39 (Analogue of Jatrorrhizine).


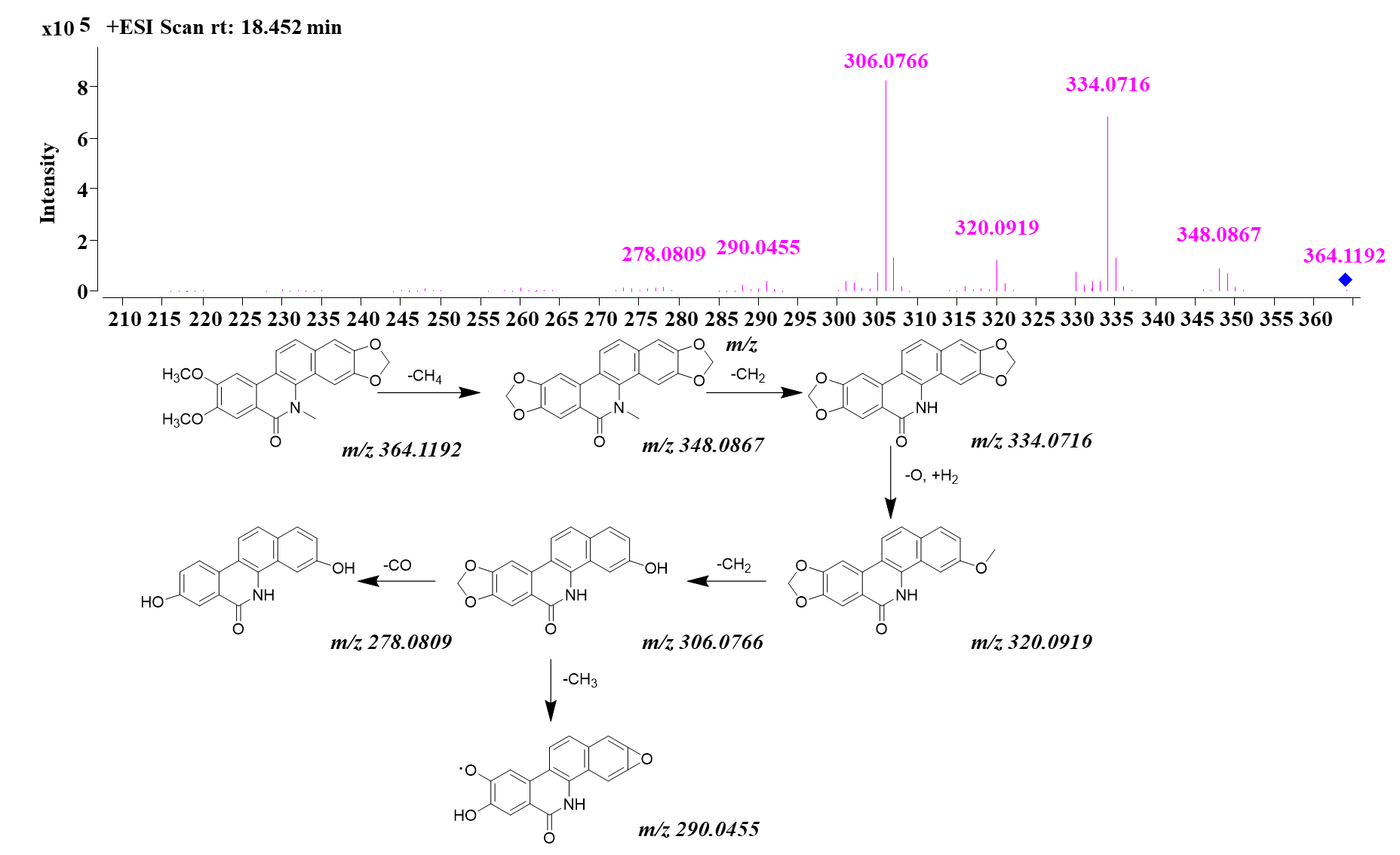
 FIGURE S38 Tentative fragmentation pathway and mass spectrogram of compound 41 (Oxynitidine).


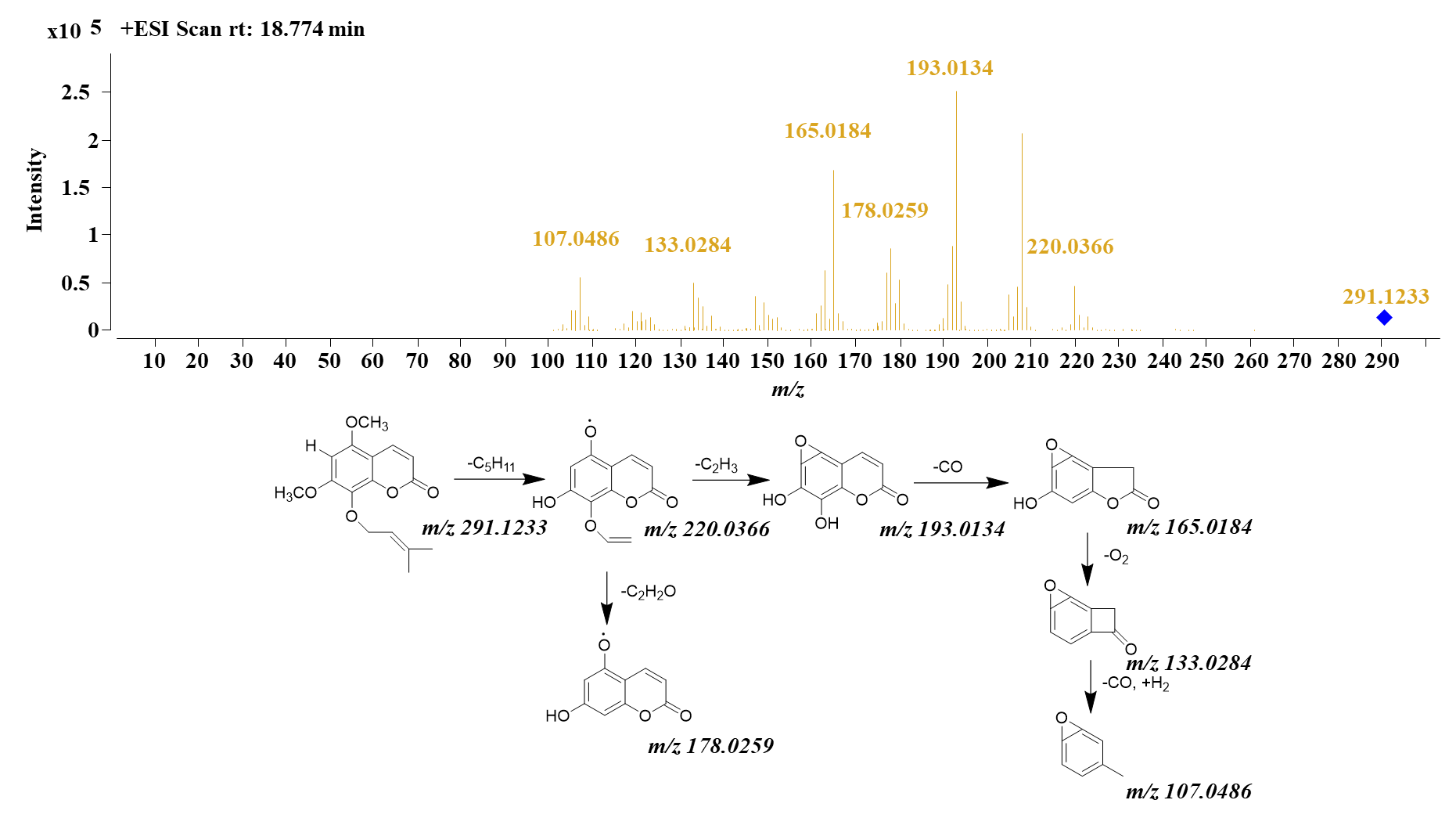
 FIGURE S39 Tentative fragmentation pathway and mass spectrogram of compound 42 (5,7-Dimethoxy-8- (3-methyl-2-butenoxy) coumarin).


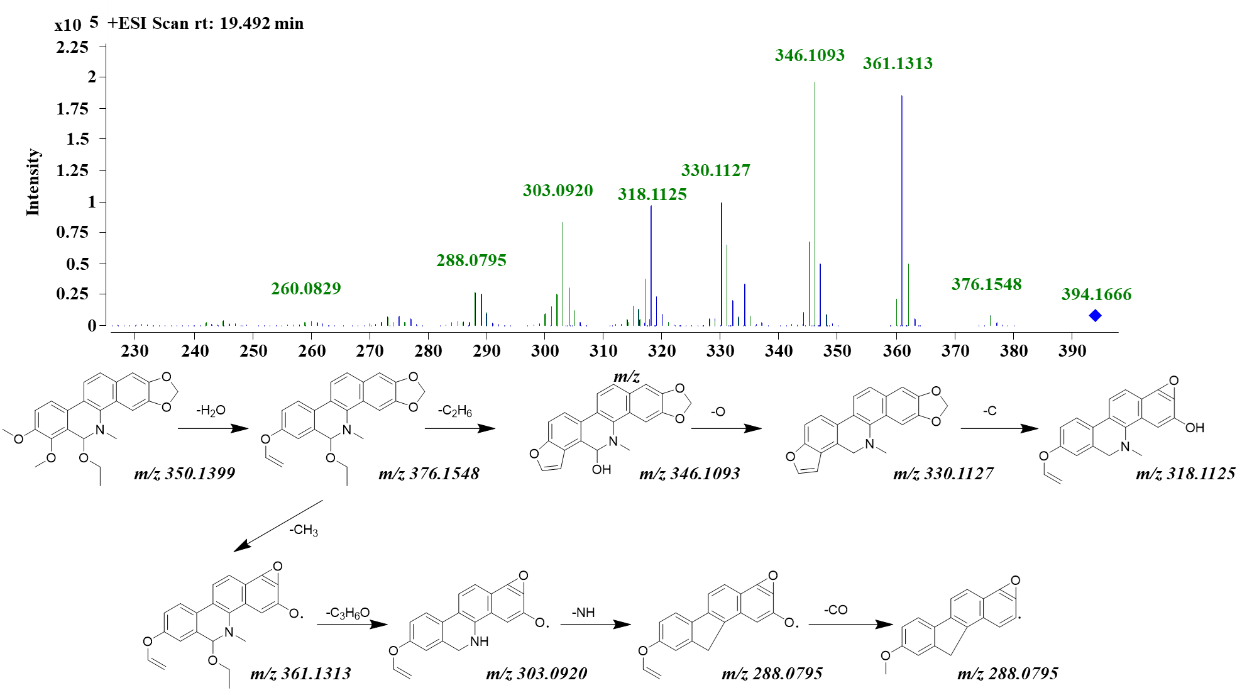
 FIGURE S40 Tentative fragmentation pathway and mass spectrogram of compound 43 (6-ethoxychelerythrine).


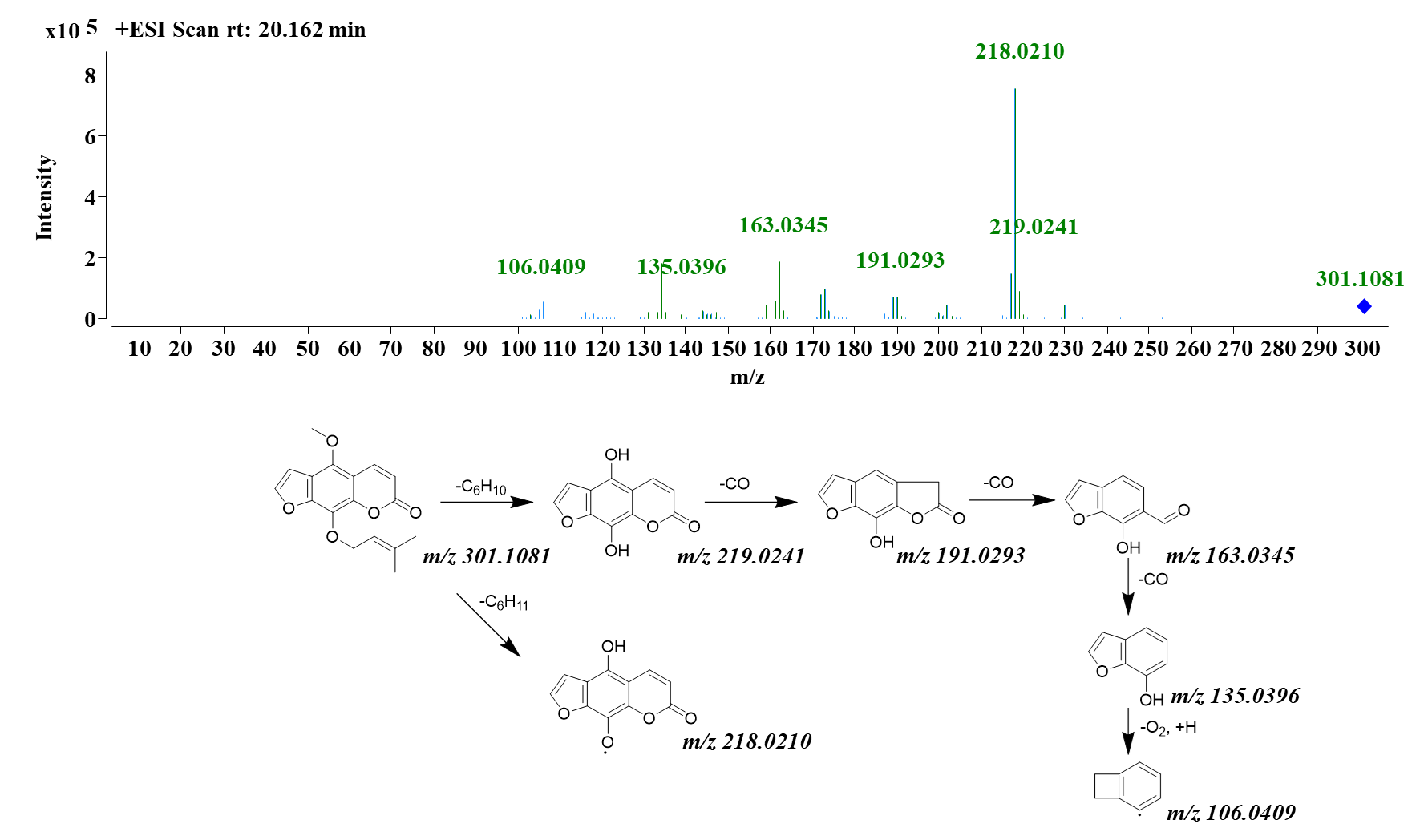
 FIGURE S41 Tentative fragmentation pathway and mass spectrogram of compound 44 (Phellopterin).


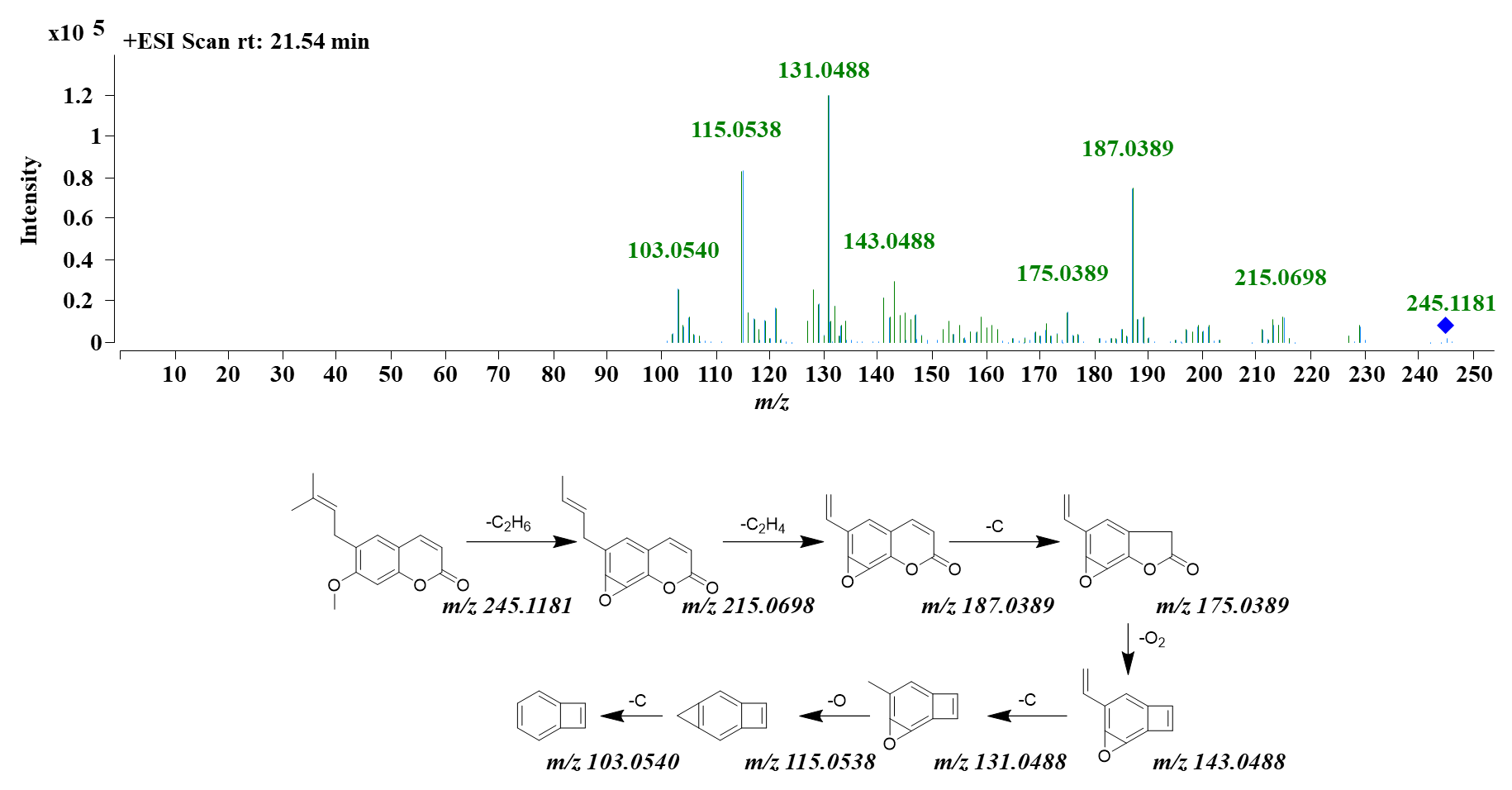
 FIGURE S42 Tentative fragmentation pathway and mass spectrogram of compound 45 (Suberosin).


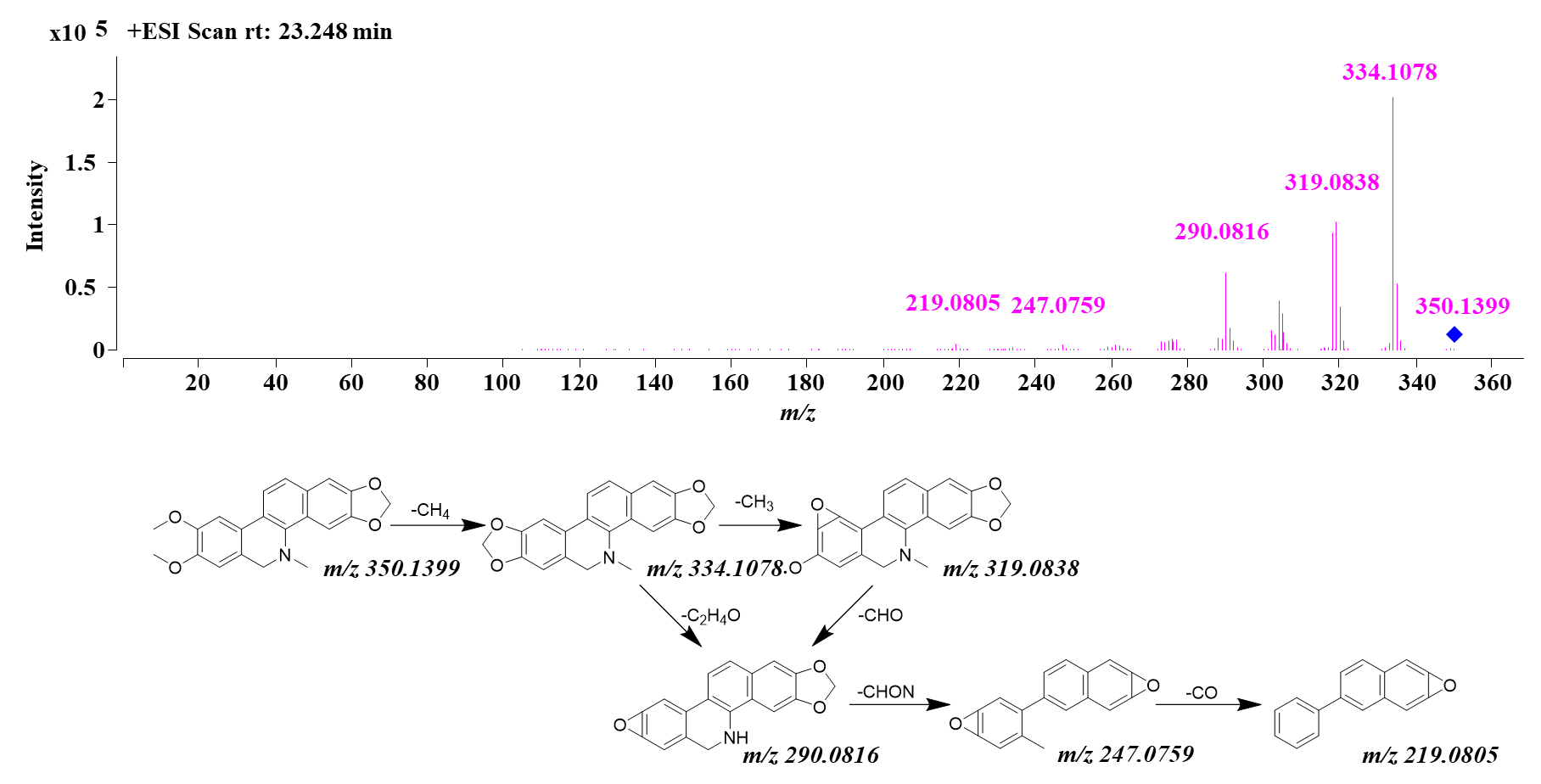
 FIGURE S43 Tentative fragmentation pathway and mass spectrogram of compound 46 (Dihydronitidine).


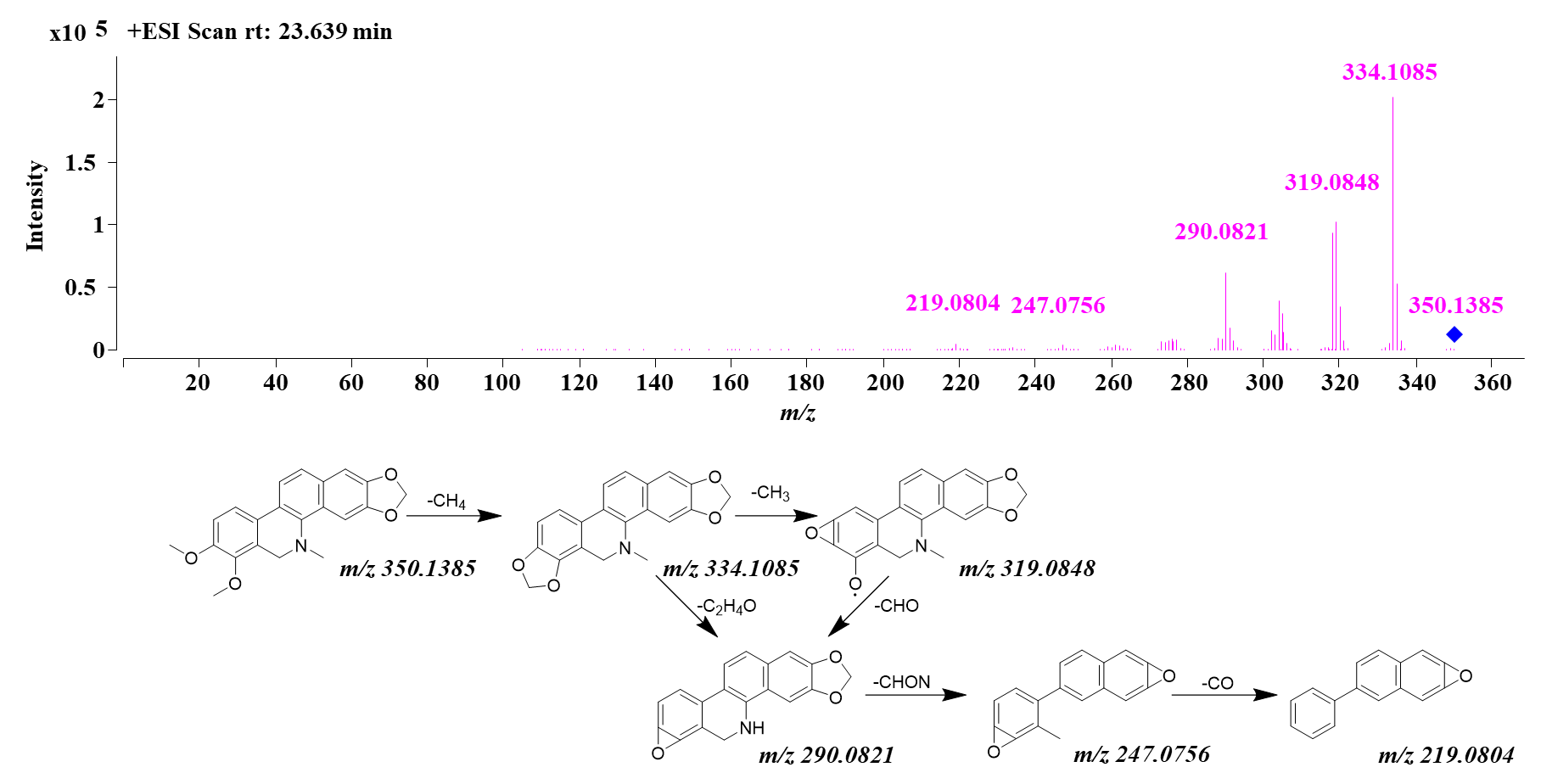
 FIGURE S44 Tentative fragmentation pathway and mass spectrogram of compound 47 (Dihydrochelerythrine).


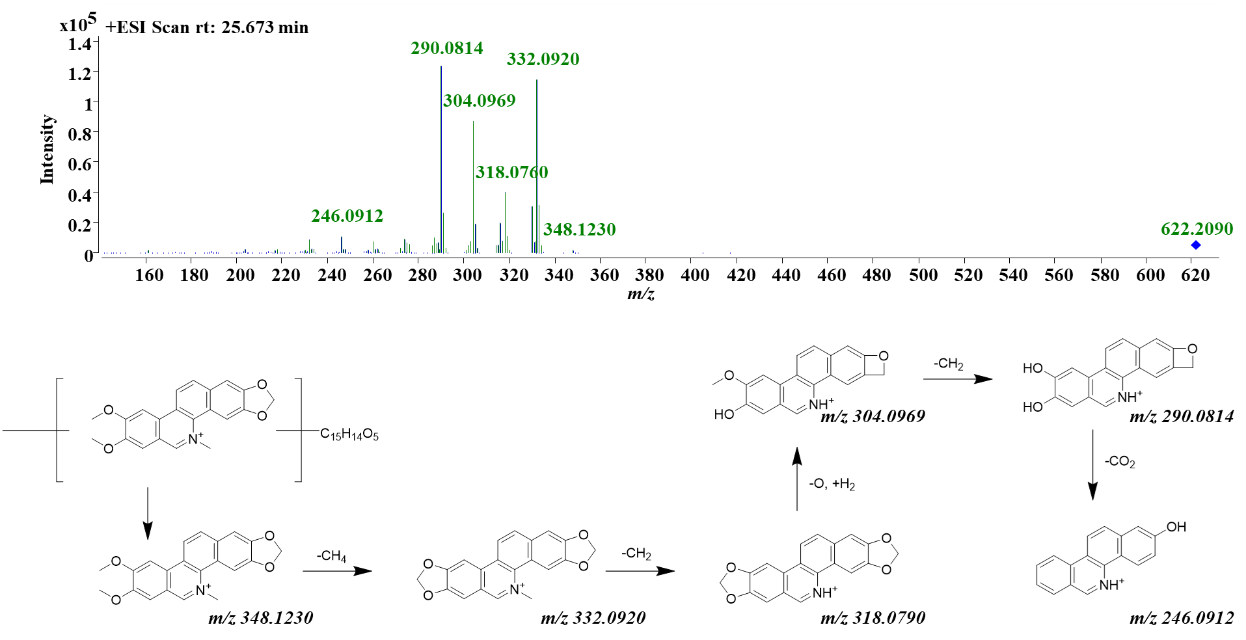
 FIGURE S45 Tentative fragmentation pathway and mass spectrogram of compound 48 (Analogue of Nitidine).


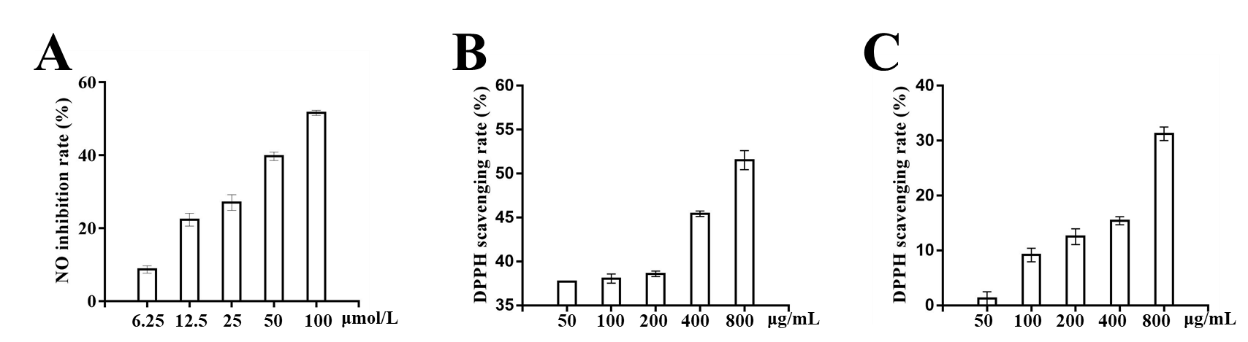


FIGURE S46 The NO inhibitory effects of nitidine (A); The DPPH scavenging effects of chelerythrine (B); The DPPH scavenging effects of hesperidin (C).
